# Eight-year longitudinal study of whole blood gene expression profiles in individuals undergoing long-term medical follow-up

**DOI:** 10.1038/s41598-021-96078-0

**Published:** 2021-08-16

**Authors:** Yoshio Sakai, Alessandro Nasti, Yumie Takeshita, Miki Okumura, Shinji Kitajima, Masao Honda, Takashi Wada, Seiji Nakamura, Toshinari Takamura, Takuro Tamura, Kenichi Matsubara, Shuichi Kaneko

**Affiliations:** 1 Department of Gastroenterology, Kanazawa University Hospital, 13-1 Takara-machi, Kanazawa, Ishikawa 920-8641 Japan; 2 System Biology, Graduate School of Advanced Preventive Medical Sciences, Kanazawa University, Kanazawa, 920-8641 Japan; 3 Department of Endocrinology and Metabolism, Graduate School of Medical Sciences, Kanazawa University, Kanazawa, 920-8640 Japan; 4 Department of Nephrology, Kanazawa University Hospital, Kanazawa, 920-8641 Japan; 5 DNA Chip Research Inc, Tokyo, 105-0022 Japan; 6 Research and Development Center for Precision Medicine, University of Tsukuba, Tsukuba, 305-8550 Japan; 7 iLAC Co., Ltd, Tsukuba, 305-0821 Japan

**Keywords:** Microarray analysis, Medical genomics, Gene expression profiling

## Abstract

Blood circulates throughout the body via the peripheral tissues, contributes to host homeostasis and maintains normal physiological functions, in addition to responding to lesions. Previously, we revealed that gene expression analysis of peripheral blood cells is a useful approach for assessing diseases such as diabetes mellitus and cancer because the altered gene expression profiles of peripheral blood cells can reflect the presence and state of diseases. However, no chronological assessment of whole gene expression profiles has been conducted. In the present study, we collected whole blood RNA from 61 individuals (average age at registration, 50 years) every 4 years for 8 years and analyzed gene expression profiles using a complementary DNA microarray to examine whether these profiles were stable or changed over time. We found that the genes with very stable expression were related mostly to immune system pathways, including antigen cell presentation and interferon-related signaling. Genes whose expression was altered over the 8-year study period were principally involved in cellular machinery pathways, including development, signal transduction, cell cycle, apoptosis, and survival. Thus, this chronological examination study showed that the gene expression profiles of whole blood can reveal unmanifested physiological changes.

Blood circulates continuously throughout the body via the peripheral circulatory system. Peripheral blood contains a wide variety of cells. In particular, leukocytes consist of phenotypically and functionally miscellaneous cells such as granulocytes, which are myeloid-lineage cells, and lymphocytes, which are associated with the lymphatic system^1^. These cells are indispensable for protecting the body from harmful exogenous pathogens as well as endogenous emerging diseases such as cancer; thus, they potentially respond to miscellaneous alterations of the physiological condition of the body.

Gene expression profiles reflect the specific characteristics of cells as well as the physiological condition of the host^2,3^. In this sense, as the blood circulates throughout the body, gene expression analysis of whole blood cells is potentially a novel tool to assess an individual’s biological characteristics as well as the presence of “silent” diseases that do not result in detectable clinical signs and symptoms. Previously, we reported that comprehensive analysis of the gene expression profiles of peripheral blood cells can help us to understand a patient’s condition, including immunological features in diabetes and in various cancers of the digestive system such as hepatocellular carcinoma, colon cancer, and pancreas cancer^4–9^. These studies strongly suggest that certain changes in gene expression reflect alterations in the immune condition of local lesions. However, despite our comprehensive investigations of the gene expression profiles of whole blood cells using complementary DNA (cDNA) microarray analysis at a specific time point, it is not yet known how the gene expression profiles of whole blood cells change over an extended period of time in individuals without a serious disease such as malignancies. It is extremely important to determine whether and how the gene expression profiles of peripheral blood are affected, especially at the early stage of a serious illness. To assess the alterations in gene expression in peripheral blood cells associated with disease, we need to characterize the stable gene expression profiles of healthy individuals over an extended period of time.

Based on these backgrounds, in the present study, we collected blood samples from 61 individuals who were undergoing regular medical health check-ups or medical follow-up examinations over an 8-year period and comprehensively analyzed their gene expression profiles using a cDNA microarray. We found that 1509 genes were stably expressed over this 8-year period, and nearly all of these genes were involved in immune-related pathways. We identified 3251 genes whose expression was changed over the same period and were involved in the development, signal transduction, cell cycle, apoptosis, survival, chemotaxis, and immune response pathways. These results imply that the physiological and structural homeostatic features of the body change over time, whereas the immune system, which involves central immune regulation, antigen-presenting cells, and related immune cells, is maintained in a relatively stable state.

## Methods

### Participants

The participants were individuals who regularly visited the Public Central Hospital of Matto Ishikawa for medical check-ups. Except for some participants who had a pre-existing clinical condition such as hypertension, dyslipidemia, or diabetes (Table 1), all of the other participants were considered to be disease-free. Every 4 years from 2008 to 2016, all participants, who were fully informed of the nature of the study and provided written informed consent to participate, were registered. The study protocol was approved by the internal review boards of the Public Central Hospital of Matto Ishikawa and Kanazawa University, and the study was conducted in accordance with the principles of the Declaration of Helsinki.

**Table 1:** Clinical features of the 61 individuals which were examined for expression of whole blood cells in 2008, 2012 and 2016.

Clinical parameters	Males (n = 34) and Females (n = 27); n = 61					
	2008		2012		2016	
	Average	± SD	Average	± SD	Average	± SD
Age (years)	50.6	10.0	54.6	10.0	58.6	10.1
Height (cm)	164.2	8.2	163.9	8.2	163.7	8.2
Weight (kg)	61.6	9.6	61.2	9.1	61.2	9.3
BMI (kg/m^2^)	22.8	2.6	22.8	2.5	22.8	2.7
Fat ratio (%)	27.0	6.0	26.7	6.1	26.9	6.1
Fat mass (Kg)	n/a	n/a	16.2	4.4	16.5	4.4
Fat free ratio (%)	n/a	n/a	44.6	7.8	45.1	8.2
Muscle ratio (%)	n/a	n/a	42.2	7.4	42.7	7.8
Bone mass (Kg)	n/a	n/a	2.4	0.4	2.4	0.4
Systolic blood pressure (mmHg)	123	16	122	14	121	13
Diastolic blood pressure (mmHg)	78	11	75	9	73	11
HbA1c (JDS) (%)	5.1	0.3	5.1	0.3	5.2	0.4
Total cholesterol (mg/dL)	203	35	199	32	201	36
TG (mg/dL)	130	113	111	71	103	64
HDL-cholesterol (mg/dL)	59	15	56	14	58	15
LDL-cholesterol (mg/dL)	118	28	115	26	117	32
AST (GOT) (IU/L)	22	8	25	14	23	8
ALT (GPT) (IU/L)	22	13	24	16	21	12
Gamma GTP (IU/L)	47	54	54	112	33	26
White blood cell count (× 10^3^/ml)	5.6	1.6	5.1	1.5	5.0	1.3
Hemoglobin count (g/dL)	14.2	1.4	14.1	1.2	13.8	1.6
Hematocrit count (%)	42.6	3.4	42.6	3.1	41.7	4.0
Platelet count (× 10^4^/ml)	23.5	5.1	23.2	5.6	23.7	6.1
Hypertension (n patients out of 61)	11/61	n/a	16/61	n/a	19/61	n/a
Dyslipidemia (n patients out of 61)	9/61	n/a	13/61	n/a	13/61	n/a
Diabetes (n patients out of 61)	10/61	n/a	14/61	n/a	19/61	n/a

### Study design and enrollment

The study was designed to analyze the gene expression features of whole blood of the registered individuals over an 8-year period from 2008. The number of registered participants was 61.

### Whole blood RNA isolation and gene expression analysis using cDNA microarray analysis

Peripheral whole blood was collected periodically from the participants in 2008, 2012, and 2016, placed in PAXgene Blood RNA Tubes (PreAnalytiX GmbH, Hombrechtikon, Switzerland) to stabilize the RNA, and stored as per the manufacturer’s protocol. Total RNA was extracted using a PAXgene Blood RNA Kit (PreAnalytix), amplified, and labeled with Cy3 by using a Low Input Quick Amp Labeling Kit (Agilent Technologies, Santa Clara, CA). cRNA hybridization was performed using an Agilent SurePrint G3 Human Gene Expression 8 × 60 K v2 Microarray (Design ID:039494) and detected with a G2600D DNA Microarray Scanner (Agilent Technologies). The intensity value of each scanned feature was quantified using Agilent Feature Extraction Software (version 11.5.1.1, https://www.agilent.com/en/genomics-software-downloads; Agilent Technologies). To confirm the reproducibility and stability of gene expression by the DNA microarray method, the extracted blood RNA samples were analyzed twice for the transcribed mRNAs (Supplementary Fig. S1); signal intensity was adjusted using the quantile normalization method^10^. After excluding poorly annotated probes and low-signal probes, 10,590 probes were extracted for further statistical analysis.

### Data analysis of gene expression

The 10,590 pre-filtered genes were used to identify differentially and non-differentially expressed genes for the years 2008, 2012, and 2016. BRB-ArrayTools v.4.6.1 (http://linus.nci.nih.gov/BRB-ArrayTools.html^11^ was used for Class Comparison analysis; hence, for the identification of differentially/non-differentially expressed genes and for Geneset Class Comparison analysis for the identification of the involved cell types, multiple genes were reduced to one per gene symbol by using the maximally expressed gene measured by average intensity across arrays. MetaCore software (version 21.2 build 70,500, https://portal.genego.com/; Clarivate Analytics, Philadelphia, PA) was used, as described previously^12^, for enrichment analyses of the pathway maps of the differentially and non-differentially expressed genes.

In the second analysis, we used the 10,590 pre-filtered genes to perform deconvolution analysis with the analytical tool CIBERSORTx (https://cibersortx.stanford.edu/)^13,14^, and obtained an estimation of the abundance of cell types by using the 22 pre-defined immune cell types present as a reference. Quantile normalization was applied, followed by bulk mode batch correction for the removal of technical differences between the reference signature matrix profiles and the investigated set of samples; 100 permutations were performed for significance analysis.

## Results

### Clinical features and overall gene expression profiles of 61 participants over an 8-year period

**Figure 1:**
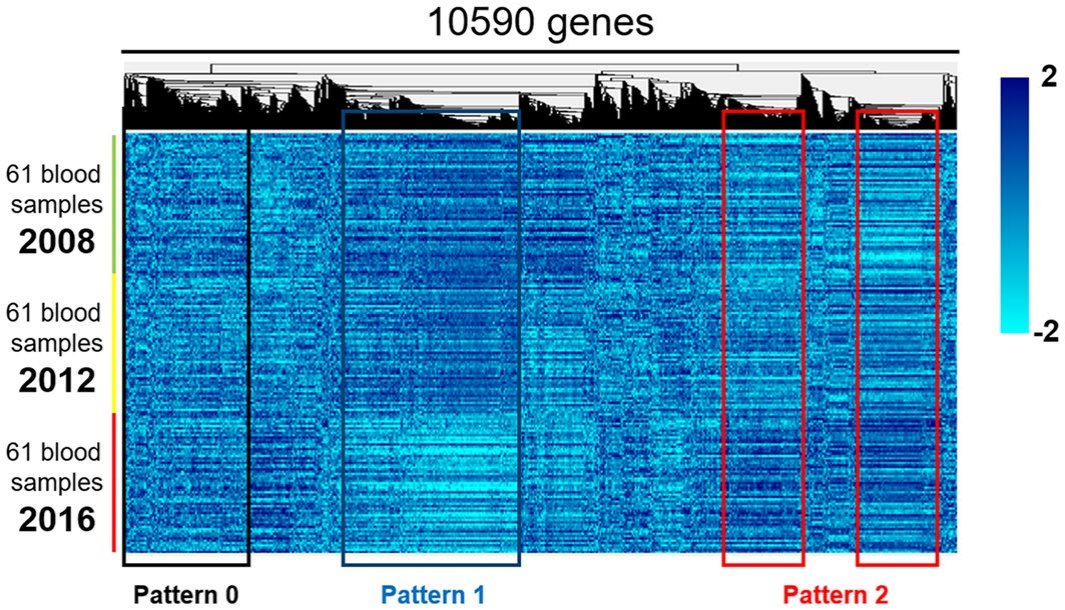
Heatmap of 10,590 genes that passed the preliminary filtering criteria, showing a distinct pattern of expression. The 10,590 genes that passed the filtration quality check suggested a pattern of stable gene expression (circumscribed by the black line; Pattern 0), whereas some genes had altered expression during the 8-year observation period. Specifically, there was a group of genes with downregulated expression in 2016 (circumscribed by the aqua blue line; Pattern 1) and a second group of genes with upregulated expression (circumscribed by the red line; Pattern 2) compared with 2008 and 2012. Blood samples from 61 individuals were used for each time point.

**Figure 2:**
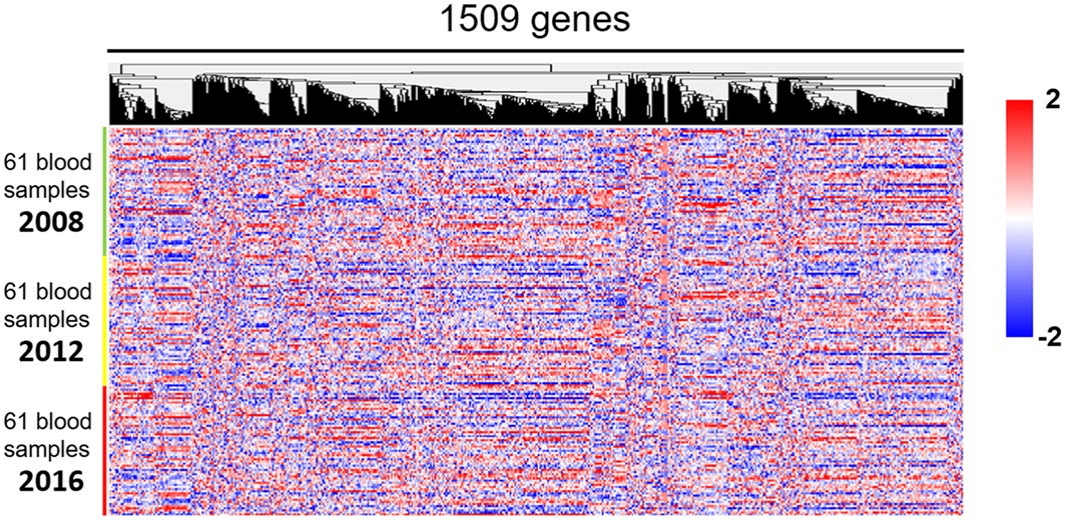
Identification of 1509 non-differentially expressed genes between the years 2008, 2012, and 2016. Among the 10,590 pre-filtered genes, the expression of 1,509 genes did not change over time and they were defined as the most representative stably expressed genes (Pattern 0; F-test parametric *p-*value > 0.2).

**Figure 3:**
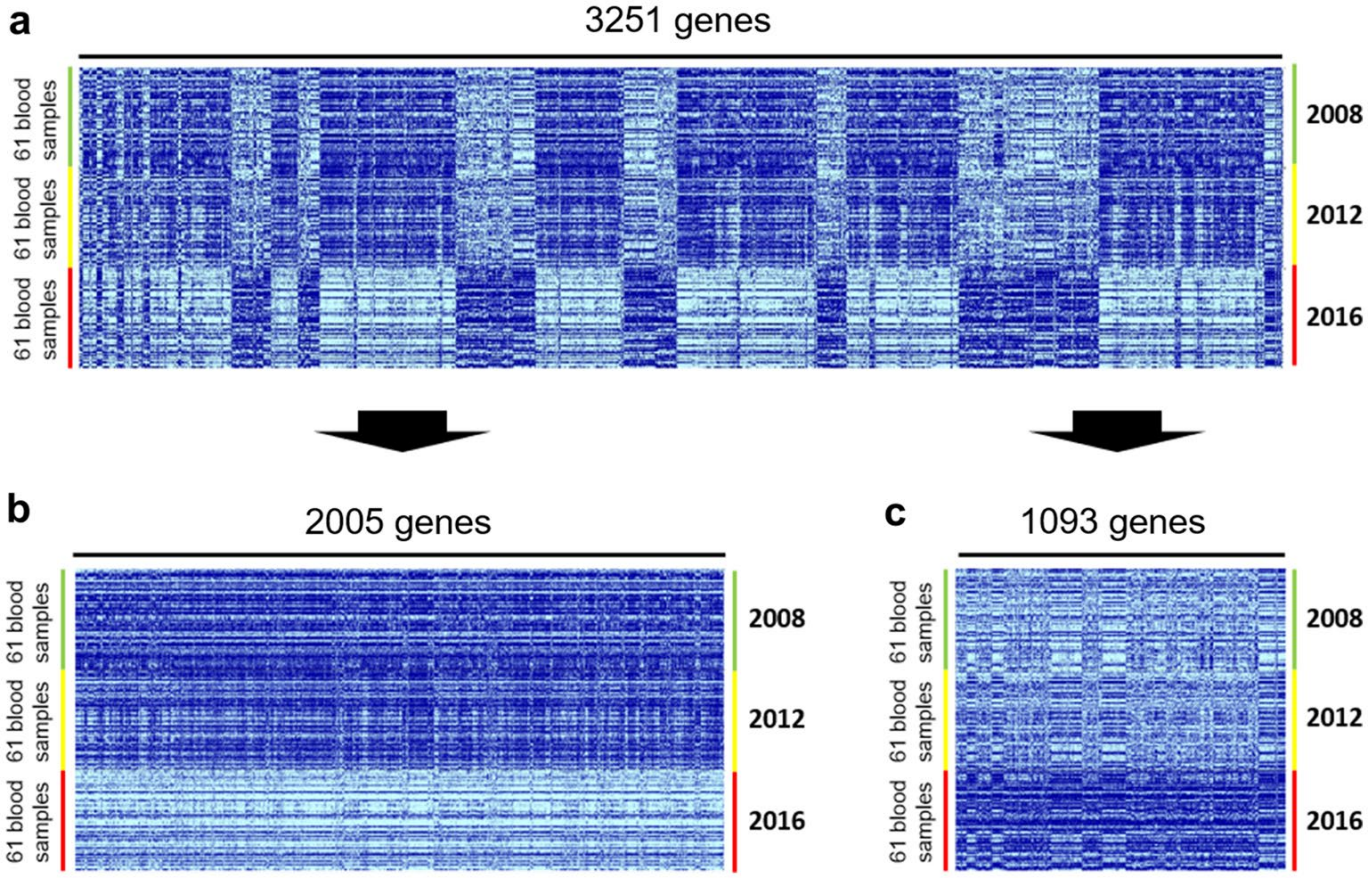
Identification of 3251 differentially expressed genes, followed by stratification of the genes into downregulated (Pattern 1) or upregulated (Pattern 2) genes in the years 2008, 2012, and 2016. (**a**) Heatmap of the 3251 genes with altered expression over the 8-year monitoring period (F-test parametric *p*-value < 1.0 × 10^–4^, false discovery rate < 1.0 × 10^–3^). (**b**) Heatmap of the 2005 genes that were downregulated over time; the expression of each gene in 2016 was lower than its expression in 2008 and 2012 (Pattern 1). (**c**) Heatmap of the 1093 genes that were upregulated over time; the expression of each gene in 2016 was higher than its expression in 2008 and 2012 (Pattern 2).

**Figure 4:**
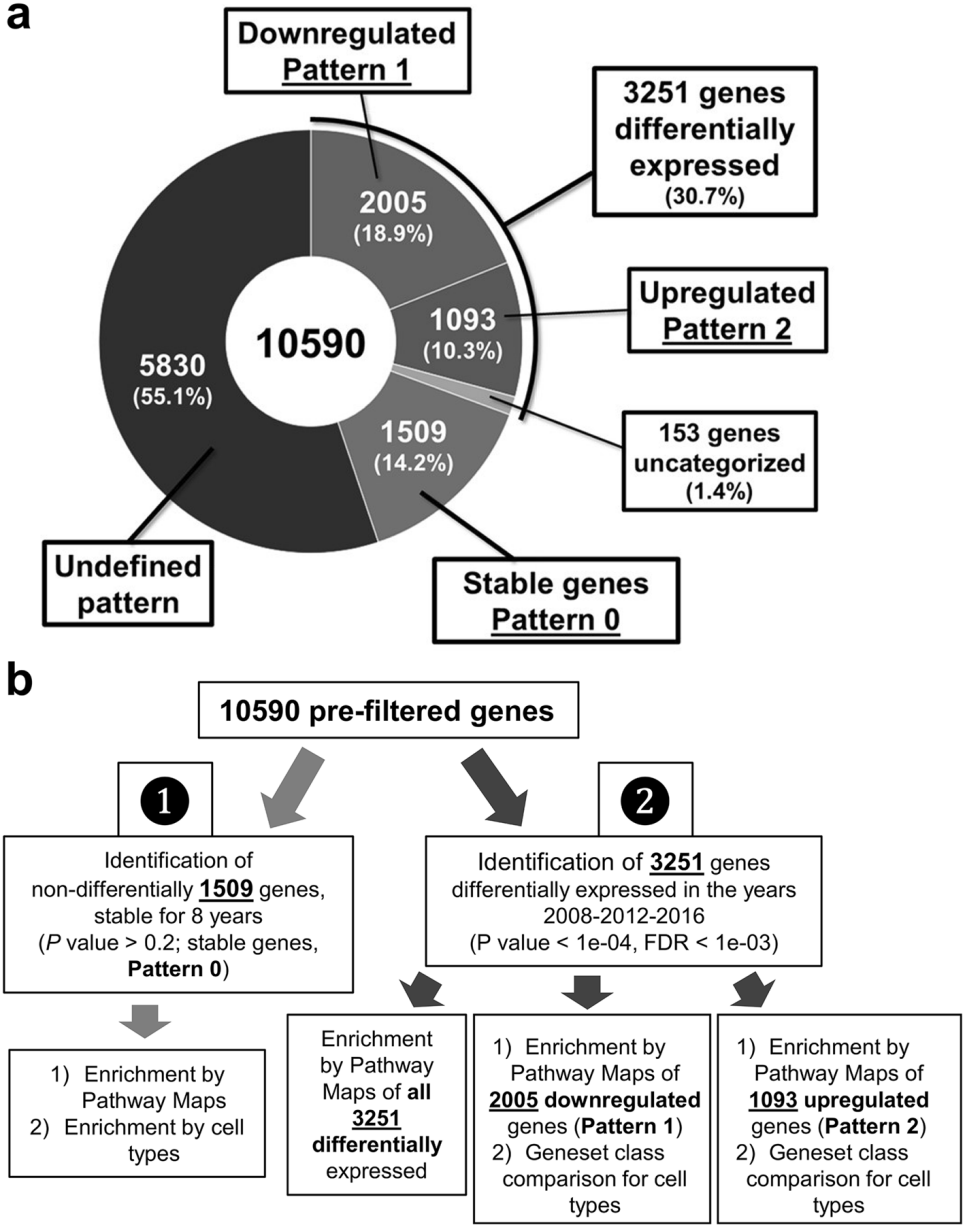
Percentage fraction of stable and altered genes within the 10,590 genes and relative flow analysis chart. (**a**) Pie chart indicating the percentage of stably expressed genes (Pattern 0), downregulated genes (Pattern 1), and upregulated genes (Pattern 2) within all 10,590 pre-filtered genes. (**b**) Analysis flow for enrichment and Geneset Class Comparison analyses.

The clinical features of the 61 participants are shown for 2008–2016 (Table 1); overall, there were no major differences in their clinical features after 8 years except for age. Using the cDNA microarray system, we obtained gene expression data for 2008, 2012, and 2016. The number of genes whose expression data passed the quality check was 10,590 genes; the gene expression heat-map of these genes implied a stable expression pattern (Pattern 0) over the 8-year observation period, whereas some genes demonstrated altered expression patterns. Specifically, the expression of a group of genes was downregulated in 2016 compared with 2008 and 2012 (Pattern 1), whereas the expression of a second group of genes was upregulated in 2016 compared with 2008 and 2012 (Pattern 2; Fig. 1). Among the 10,590 genes, we identified 2010, 1509, and 754 genes whose expression did not change significantly over time (F-test parametric *p*-value > 0.1, 0.2, and 0.5, respectively). For the following analyses, we chose the set of 1,509 genes as the most representative non-differentially expressed (stable) genes (F-test parametric *p*-value > 0.2; Figs. 2 and 4a). In contrast, the expression of 3,251 genes was changed over the 8-year period (F-test parametric *p*-value < 1.0 × 10^–4^, false discovery rate < 1.0 × 10^–3^; Figs. 3a and 4a) with two major patterns of change: the first group consisted of 2,005 genes whose expression was significantly downregulated in 2016 compared with 2008 and 2012 (Pattern 1; Figs. 3b and 4a), and the second group consisted of 1093 genes whose expression was significantly upregulated in 2016 compared with 2008 and 2012 (Pattern 2; Figs. 3c and 4a); 153 genes of the 3251 genes were not categorized (Fig. 4a) into either of these two patterns and were excluded from the following analyses. Finally, we performed Enrichment Analyses and Geneset Class Comparison of the 1509 stably expressed genes, 3251 differentially expressed genes, 2005 downregulated genes, and 1093 upregulated genes (Fig. 4b).

### Biological features of genes whose expression was not altered during the 8-year study period

We next assessed the features of the 1509 stably expressed genes by analyzing which pathways they were involved in by using MetaCore software. Among the top 16 pathways (Table 2, Supplementary Table S1), all of the maps indicated immune system-related pathways. The most distinctive pathways were immune response-related, which were represented by type 1 and type 2 interferon-mediated antigen presentation and T cell regulation (Table 2, Supplementary Table S1). The other immune-related pathways were involved in miscellaneous cell roles as well as the immune cell chemotaxis system via SDF-1/CXCR4. Key genes were *CD3D*, *LCK*, and *ZAP70*, which are related to signal transduction in T cell activation^15,16^, and *CCL2*, which is a chemokine capable of recruiting monocytes, dendritic cells, and memory T cells^17,18^. These pathways suggested that the overall immune system was rather stable over an 8-year period in middle-aged people, at least in the context of conventional immune system categories.

**Table 2:** Summary table of MetaCore enrichment analyses for differentially and non-differentially (stable) genes.

1509 non-differentially genes (*P* value > 0.2; stable genes; Fig. 2)	3251 genes differentially expressed (*P* value < 1e–04, FDR < 1e–03; Fig. 3)		
MetaCore enrichment by pathway maps of stable 1509 genes (Pattern 0; Supplementary Table S1)	MetaCore enrichment of all 3251 genes differentially expressed (Supplementary Table S3)	MetaCore enrichment by pathway maps of 2005 downregulated genes (pattern 1; Supplementary Table S4)	MetaCore enrichment by pathway maps of 1093 upregulated genes (pattern 2; Supplementary Table S5)
*Top 16 pathways related to*: *Immune response*: induction of the antigen presentation machinery by IFN-gamma, IFN-alpha/beta signaling via JAK/STAT, IFN-alpha/beta signaling via MAPKs, antigen presentation by MHC class II, IFN-gamma signaling via MAPK, inhibitory PD-1 signaling in T cells *Immune related pathway (MetaCore noncategorized pathways)*: putative role of Tregs in COPD, role of integrins in eosinophil degranulation in asthma, down-regulation of mast cell functions through ITIM-containing inhibitory receptors in asthma, SLE genetic marker-specific pathways in T cells, breakdown of CD4 + T cell peripheral tolerance in type 1 diabetes mellitus, maturation and migration of dendritic cells in skin sensitization, NETosis in SLE, neutrophil chemotaxis in asthma *Chemotaxis*: SDF-1/CXCR4-induced chemotaxis of immune cells	*Top 16 pathways related to: development*: thromboxane A2 signaling pathway, VEGF signaling via VEGFR2-generic cascades, role of HDAC and calcium/calmodulin-dependent kinase (CaMK) in control of skeletal myogenesis *Oxidative stress*: ROS-induced cellular signalling *Cell cycle*: influence of ras and rho proteins on G1/S *Transition signal transduction*: calcium-mediated signaling, mTORC1 downstream signaling, adenosine A2B receptor signaling pathway, PKA signaling, MIF signaling pathway *Immune response*: platelet activating factor/PTAFR pathway signaling, B cell antigen receptor (BCR) pathway *Chemotaxis*: lysophosphatidic acid signaling via GPCRs *Apoptosis and survival*: NGF/TrkA PI3K-mediated signaling	*Top 16 pathways (DOWNREGULATED) related to*: *Signal transduction*: mTORC1 downstream signaling, calcium-mediated signalling *Apoptosis and survival*: role of PKR in stress-induced apoptosis, TNFR1 signaling pathway, endoplasmic reticulum stress response pathway, NGF/TrkA PI3K-mediated signalling *Chemotaxis*: lysophosphatidic acid signaling via GPCRs *Cell cycle*: influence of ras and rho proteins on G1/S transition *Proteolysis*: Putative SUMO-1 pathway *Development*: thromboxane A2 signaling pathway, the role of GDNF ligand family/RET receptor in cell survival, growth and proliferation *DNA damage*: ATM/ATR regulation of G2/M checkpoint: cytoplasmic signalling *Transport*: RAN regulation pathway *Immune response*: TLR2 and TLR4 signaling pathways	Top 16 pathways (UPREGULATED) related to: *Immune response*: platelet activating factor/PTAFR pathway signalling *Cytoskeleton remodeling*: regulation of actin cytoskeleton organization by the kinase effectors of rho GTPases *Development*: thromboxane A2 signaling pathway, thrombospondin 1 signaling, WNT/Beta-catenin signaling in the cytoplasm *Blood coagulation*: GPCRs in platelet aggregation *Signal transduction*: adenosine A2B receptor signaling pathway, adenosine A3 receptor signaling pathway, MIF signaling pathway, PKA signalling *Neurophysiological process*: constitutive and regulated NMDA receptor trafficking *Apoptosis and survival*: Phosphorylation in TNF-alpha-induced NF-kB signalling Platelet activation as a result of endothelial dysfunction after stenting

To identify which immune-mediating cells were related to the 1,509 genes stably expressed in peripheral blood, we performed enrichment analysis by cell type (Supplementary Table S2). The identified cell types included NK cells, dendritic cells, monocytes, neutrophils, CD8+ T cells, and regulatory T cells, which are compatible with the results of the pathway map analysis described above.

### Biological features of genes with altered expression in peripheral blood cells

We also examined the biological characteristics of genes whose expression was changed over the 8-year study period. Biological pathway map analysis of all genes whose expression was altered indicated that they had roles in the development, oxidative stress, cell cycle, signal transduction, apoptosis and survival, chemotaxis, and immune response pathways (Table 2, Supplementary Table S3), suggesting that cellular homeostatic features were affected. Pathway analysis of the 2005 downregulated genes (Pattern 1) showed that they were related to the signal transduction, apoptosis and survival, cell cycle, proteolysis, development, DNA damage, and transport pathways (Table 2, Supplementary Table S4). Key genes were *AKT2*, which is a putative oncogene^19^, and *CREB1*, which is involved in cell proliferation^20^. Pathway analysis of the 1093 upregulated genes (Pattern 2) indicated that they were related to the cytoskeleton remodeling, development, blood coagulation, signal transduction, neurophysiological process, apoptosis and survival, and immune response pathways (Table 2, Supplementary Table S5). Key genes included *PIK3CB* and *NFKB1*, which are related to cell growth and apoptosis^21,22^. Furthermore, the cell types involved with the downregulated genes were suggested to be B cells and T helper cells (Supplementary Table S6), whereas the upregulated genes were related to platelets and CD34+ megakaryocyte progenitors (Supplementary Table S7).

### Stability and variability of whole blood cell types over time

**Figure 5:**
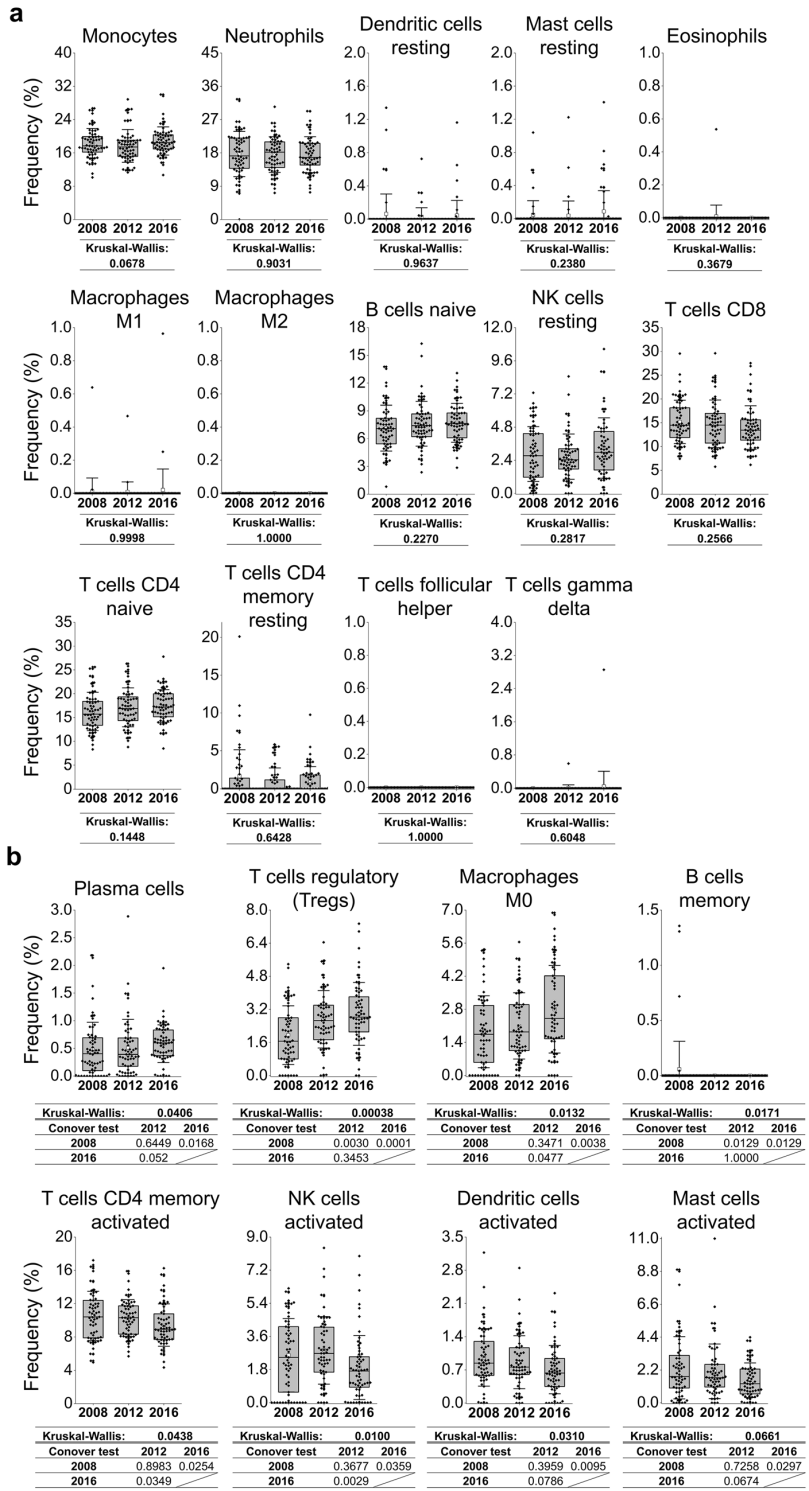
Deconvolution analysis for the estimation of cell types in blood and variability in cell frequency over time. We used 10,590 genes for deconvolution analysis/digital cytometry with the analytical tool CIBERSORTx. Cell frequencies were obtained by using 22 reference immune cell types. Out of the 22 immune cell types investigated, we obtained (**a**) 14 cell types whose frequency remained unaltered and (**b**) 8 cell types whose frequency changed over time; the summation of the frequencies of the 22 immune cells corresponds to the total frequency of 100%. (**a**, **b**) n = 61 for the years 2008, 2012, and 2016. Grey boxes indicate the interquartile range (25–75% percentile range), the median is indicated by a horizontal line within the box, and the mean is indicated by the small white square; whiskers represent ± 1 SD from the mean. The Kruskal–Wallis test and post hoc Conover test were performed as statistical analysis and are shown under each relative graph.

Next, we examined the abundance of specific cell types in blood and their variability over time by deconvolution analysis (CIBERSORTx). Out of the 22 immune cell types investigated, we found 14 cell types whose frequency was unaltered (Fig. 5a) and 8 cell types whose frequency changed over time (Fig. 5b). The unaltered 14 cell types were as follows: NK resting cells, monocytes, neutrophils, CD8+ T cells, naïve CD4+ T cells, resting memory CD4+ T cells, follicular helper T cells, γδ T cells, resting dendritic cells, naïve B cells, M1 and M2 macrophages, resting mast cells, and eosinophils, which were consistent with the identified immune-mediating cells (Supplementary Table S2) related to the analysis of the 1,509 stably expressed genes. Regarding the 8 cell types that changed in frequency over time, 3 cell types increased in frequency (plasma cells, regulatory T cells, and M0 macrophages), whereas 5 cell types decreased in frequency (B memory cells, activated memory CD4+ T cells, activated NK cells, activated dendritic cells, and activated mast cells, although this last population only had a tendency to decrease in frequency over time).

## Discussion

In the present study, we observed the gene expression profiles of whole blood cells over an 8-year period in 61 individuals. The average age at registration was approximately 50 years. We observed that the expression of 1509 genes was stable in all individuals over this 8-year period. Biological pathway map analysis for these stably expressed genes indicated that they had roles in immune response-related pathways, including antigen-presenting cells and interferon signaling; consistently, the involved cells included antigen-presenting cells and lymphocyte-lineage cells. In contrast, we found 3251 genes whose expression was altered over the 8-year study period; within these genes, we observed that there were two groups that showed either downregulated expression in 2016 (Pattern 1) or upregulated expression in 2016 (Pattern 2) compared with 2008 and 2012. As a whole, the 3251 altered genes were involved in the signal transduction, apoptosis and survival, cell cycle, proteolysis, development, DNA damage, transport, cytoskeleton remodeling, blood coagulation, and neurophysiological process pathways, in addition to a minor contribution to immune response pathways, suggesting that the cellular machinery is the major altered biological process. Furthermore, by examining the abundance of specific cell types in blood and their variability over time using deconvolution analysis/digital cytometry, we confirmed that the cell types that did not change over time were related to lymphocyte-lineage cells and monocytic and granulocytic cell types. Cell subpopulations whose frequency increased over time were represented by plasma cells, regulatory T cells, and M0 macrophages, whereas subpopulations whose frequency decreased over time consisted of memory B cells, memory CD4+ T cells, NK cells, dendritic cells, and mast cells. These cells that changed in frequency are interesting targets for future detailed investigations.

Blood is a very useful and convenient sample for medical examination^23^. It consists of white blood cells, red blood cells, and platelets as cellular components; among them, white blood cells contain immune-mediating cells that respond to abnormal endogenous or exogenous pathogens or lesions^24^. Their reaction to lesions is associated with alterations in gene expression^25–30^. Because humans have a biological system to maintain physiologic homeostasis, gene expression in whole blood cells should be generally steady in the absence of disease. However, as humans age, there are substantial changes to the biological function of the body^31^. In this sense, to monitor the features of stable health, it is important to characterize the total stable and altered gene expression profiles of peripheral blood cells over a substantial period of time. Genes with stable expression in peripheral blood have the potential to be used for monitoring; genes whose expression is altered should be studied further to determine whether the involved biological processes are related to changes in the participants’ fundamental health condition over the 8-year study period.

The current study provided precious information suggesting important biological features; the stable expression of genes in whole peripheral blood cells was related mostly to the central immune regulation system, including antigen-presenting cells and interferon signaling. As we mentioned above, the average age of the participants when the study was initiated was 50 years and the observation period was 8 years. To date, we have reported that gene expression analysis of whole blood cells is beneficial for understanding diseases such as cancer and metabolic syndrome, including diabetes mellitus^4,7,12,32^. From 50 to 60 years of age, various common diseases may develop due to increasing age^33^; thus, laboratory medicine using gene expression analysis of whole blood cells might have the potential to be a novel avant-garde tool for understanding and obtaining an early diagnosis of diseases. The high number of 3251 genes whose expression was altered over the 8-year study period indicated the general constituents of tissues or cells. The importance of these biological features should be investigated further in terms of intra-individual health conditions. It follows that future analyses should be focused on a comparison of groups stratified by specific diseases or age. Taking the present study results and our previous studies of changes in gene expression in cancers of the digestive system, the chronological examination of the gene expression profiles of whole blood has the potential to be a novel laboratory-based clinical approach.

In conclusion, we reported the long-term gene expression profiles of whole blood cells in 61 individuals who underwent regular medical health check-ups or outpatient clinic visits. The current findings regarding the existence of stably expressed genes in individuals over time might be useful for detecting unmanifested diseases, specifically, by observing the altered gene expression of these typically stable genes.

## Supplementary Information

Supplementary Information.

## Abbreviation

cDNA: Complementary DNA
