# Supplementary material for: Eight-year longitudinal study of whole blood gene expression profiles in individuals undergoing long-term medical follow-up
Source: Sci Rep. 2021 Aug 16;11:16564. doi: 10.1038/s41598-021-96078-0 (PMC8368195; doi:10.1038/s41598-021-96078-0)
Supplement: Supplementary file 1 — Supplementary Information. [file 41598_2021_96078_MOESM1_ESM.pdf]

Eight-year longitudinal study of whole blood gene expression profiles in individuals undergoing long-term medical follow-up

Yoshio Sakai, Alessandro Nasti, Yumie Takeshita, Miki Okumura, Shinji Kitajima, Masao Honda, Takashi Wada, Seiji Nakamura, Toshinari Takamura, Takuro Tamura, Kenichi Matsubara, Shuichi Kaneko.

Supplementary Information

Table of Contents

Supplementary Tables 2

Supplementary Table S1. MetaCore Enrichment Analysis by Pathway Maps of 1509 stable genes (Pattern 0). 2

Supplementary Table S2. Enrichment analysis by cell type (BrB ArrayTools and referenced curated gene sets) of the stable 1509 genes (Pattern 0). 11

Supplementary Table S3. Enrichment by Pathway Maps of all 3251 genes differentially expressed. 15

Supplementary Table S4. Enrichment by Pathway Maps of 2005 downregulated genes (Pattern 1). 30

Supplementary Table S5. Enrichment by Pathway Maps of 1093 upregulated genes (Pattern 2). 40

Supplementary Table S6. Gene set class comparison analysis by cell type (BrB ArrayTools) of 2005 downregulated genes (Pattern 1). 50

Supplementary Table S7. Gene set class comparison analysis by cell type (BrB ArrayTools) of 1093 upregulated genes (Pattern 2). 51

Supplementary Figures 52

Supplementary Figure S1. Gene expression stability verified by technical replicates. 52

Supplementary Tables

Supplementary Table S1. MetaCore Enrichment Analysis by Pathway Maps of 1509 stable genes (Pattern 0).

#	Maps	Total	p-value	FDR	In Data	Network Objects from Active Data
1	Immune response_Induction of the antigen presentation machinery by IFN-gamma	53	3.2123E-11	4.5518E-08	19	CIITA, HLA-DPA1, TAP1 (PSF1), HLAA, HLA-DOA, HLA-DRB1, MHC class II, HLA-F, HLAC, HLA-DQB1, RFX5, HLA-DQA1, WDR5, Cathepsin S, PSMB10, HLA-DPB1, HLAB, STAT1, HLA-DMA
2	Putative role of Tregs in COPD	30	2.7802E-09	1.9698E-06	13	L-selectin, IL7RA, MCP, STK39, CD45, GITR, ZAP70, CD3, LAT, ROR-gamma, IL-2R alpha chain, CD3 zeta, ERK1/2
3	Role of integrins in eosinophil degranulation in asthma	58	1.2009E-08	5.6723E-06	17	CCL5, Galectin-3, RNS2, FCGR3A, c-Raf-1, ERK2 (MAPK1), CCL13, CD9, Fc gamma RII alpha, ICAM1, CCR3, ECP (RNase 3), CD67, Plastin, G-protein alpha-i family, ERK1/2, FGR
4	Immune response_IFN-alpha/beta signaling via JAK/STAT	62	3.6296E-08	1.2858E-05	17	IRF7, TAP1 (PSF1), MxB, RSAD2, IL1RN, IFI27, STAT6, GBP4, ISG15, IFNAR1, PKR, CCL2, IFI6, ADAR1, XAF1, STAT1, USP18
5	Down-regulation of mast cell functions through ITIM-containing inhibitory receptors in asthma	37	5.7094E-08	1.6181E-05	13	KLRG1, SHPS-1, Fyn, IRp60, Fc epsilon RI beta, CCL2, PIRB, LAIR1, Lyn, SHP-2, DOK2, ERK1/2, Fc epsilon RI alpha
6	SLE genetic marker-specific pathways in T cells	101	1.7718E-07	4.1844E-05	21	Ubiquitin, HLA-DRB1, HLA-DRB3, MHC class II, c-Raf-1, Lck, Slp76, MeCP2, CalDAG-GEFIII, RUNX3, MHC class II beta chain, ZAP70, CD3, LAT, HLA-DRB, TRAF2, T-bet, Aiolos, CD3 zeta, ERK1/2, CD40L(TNFSF5)
7	Breakdown of CD4+ T cell peripheral tolerance in type 1 diabetes mellitus	49	3.3481E-07	6.7774E-05	14	Ku70, HLA-DRB1, MHC class II, HLA-DQB1, Lck, ZAP70, CD3, LAT, HLA-DQA1, T-bet, IL-2R alpha chain, JAK3, PD-L1, CD40L(TNFSF5)
8	Chemotaxis_SDF-1/ CXCR4-induced chemotaxis of immune cells	79	1.5809E-06	0.00024863	17	RASSF5, Pyk2(FAK2), Fyn, Lck, CD45, CALDAG-GEFI, ICAM1, ZAP70, CD3, SFK, CD3 zeta, JAK3, G-protein alpha-i family, F-Actin cytoskeleton, CXCR4, Paxillin, ERK1/2
9	Coronavirus disease-19	134	1.7007E-06	0.00024863	23	CCL5, Cathepsin L, IRF7, HLA-DPA1, HLA-DRB1, MHC class II, IL1RN, sIL2RA, HLA-DRB5, TRIF (TICAM1), TRAF3, ISG15, MyD88, MDA-5, RIG-I, CCL2, MIP-1-alpha, CD3, HLA-DPB1, PIP5KIII, STAT1, ERK1/2, HLA-DMA

Supplementary Table S1. (Continued)

#	Maps	Total	p-value	FDR	In Data	Network Objects from Active Data
10	Maturation and migration of dendritic cells in skin sensitization	41	1.7546E-06	0.00024863	12	MHC class II alpha chain, HLA-DRB1, HLA-DRB3, MHC class II, HLA-DRB4, HLA-DRB5, CCR7, ICAM1, MHC class II beta chain, HLA-DRB, TRAF2, ERK1/2
11	Immune response_IFN-alpha/beta signaling via MAPKs	73	2.4733E-06	0.00031017	16	Ubiquitin, IRF7, TAP1 (PSF1), p130, PRMT1, RSAD2, HDAC1, Lck, CD45, ISG15, IFNAR1, PKR, ZAP70, TCF7L2 (TCF4), STAT1, ERK1/2
12	Immune response_Antigen presentation by MHC class II	118	2.6267E-06	0.00031017	21	MHC class II alpha chain, Cathepsin L, Dectin-1, Fc gamma RII beta, 14-3-3 beta/alpha, Cathepsin F, IP-30, FCGR3A, MHC class II, PIP5K1A, TRIF (TICAM1), MAP1LC3B, MHC class II beta chain, MyD88, HCLS1, Cathepsin S, Cathepsin V, CLEC10A, LAMP2, ERK1/2, Tubulin (in microtubules)
13	Immune response_IFN-gamma signaling via MAPK	51	3.6453E-06	0.00039734	13	Pyk2(FAK2), MNK2(GPRK7), TIRAP (Mal), c-Raf-1, ERK2 (MAPK1), UBCH8, MNK1, MyD88, IFI16, IFI6, C/EBPbeta, STAT1, ERK1/2
14	NETosis in SLE	31	4.8653E-06	0.00049244	10	Leukocyte elastase, p67-phox, Histone H3, Fc gamma RII alpha, Alpha-defensin, Histone H2, Histone H2A, ERK1/2, Histone H1, PERM
15	Neutrophil chemotaxis in asthma	38	5.3583E-06	0.00050618	11	CCL5, FPRL1, CCR1, CCR3, CCL2, MIP-1-alpha, HSP70, G-protein alpha-i family, Tissue kallikreins, ERK1/2, IL8RB
16	Immune response_Inhibitory PD-1 signaling in T cells	53	5.7962E-06	0.00051332	13	MHC class II, Lck, ICAM1, PTEN, Eomesodermin, ZAP70, CD3, Cyclin E, SHP-2, T-bet, CD3 zeta, ERK1/2, PD-L1
17	Cytoskeleton remodeling_Reverse signaling by Ephrin-B	32	6.7196E-06	0.00053824	10	Actin cytoskeletal, c-Raf-1, Tubulin alpha, F-Actin, Ephrin-B receptors, G-protein alpha-i family, CXCR4, Paxillin, PINCH-2, Tubulin (in microtubules)
18	Stellate cells activation and liver fibrosis	70	6.8372E-06	0.00053824	15	c-Raf-1, ERK2 (MAPK1), c-Fos, PTCH1, PDGF receptor, ICAM1, MyD88, CCL2, TRAF2, Tcf(Lef), ERK1 (MAPK3), KLF6, RIPK1, TGF-beta receptor type I, PDGF-R-beta
19	Immune response_TCR alpha/beta signaling pathway	97	7.3652E-06	0.00054929	18	P2X7, Fyn, MHC class II, c-Raf-1, Lck, STK39, CD45, Slp76, TRAF3, c-Fos, ZAP70, CD3, P2X4, LAT, TRAF2, CaMK IV, RIPK1, ERK1/2
20	Immune response_Role of DPP4 (CD26) in immune regulation	47	8.4737E-06	0.00060036	12	CCL5, Ubiquitin, Fyn, MHC class II, Lck, DPP4, CD45, ZAP70, CD3, CD3 zeta, Granzyme B, ADA

Supplementary Table S1. (Continued)

#	Maps	Total	p-value	FDR	In Data	Network Objects from Active Data
21	Immune response_Role of DAP12 receptors in NK cells	55	8.998E-06	0.00060715	13	KIR2DS2, Actin cytoskeletal, Fyn, HLAC, c-Raf-1, DAP12, Lck, HLA-G, ZAP70, LAT, SHP-2, HLAB, ERK1/2
22	Chemokines in inflammation in adipose tissue and liver in obesity, type 2 diabetes and metabolic syndrome X	48	1.0722E-05	0.00069058	12	CCL5, FCGR3A, L-selectin, MHC class II, CCR5, CD45, ICAM1, CCL2, MIP-1-alpha, CMKLR1, CD3, CXCR4
23	Immune response_IL-2 signaling via ERK, PI3K, and PLC-gamma	73	1.1756E-05	0.00072426	15	IL-2R beta chain, Fyn, c-Raf-1, Lck, Cyclin D3, ERK2 (MAPK1), c-Fos, Cyclin E, Lyn, SHP-2, IL-2R alpha chain, Aiolos, JAK3, ERK1 (MAPK3), ERK1/2
24	Immune response_IL-3 signaling via JAK/STAT, p38, JNK and NF-kB	93	1.6177E-05	0.00094712	17	MHC class II, CISH, HDAC1, STAT6, Cyclin D3, Survivin, c-Fos, ICAM1, C/EBPbeta, PUMA, Lyn, IL-2R alpha chain, JAK3, Granzyme B, DPF3, STAT1, SOCS1
25	Aberrant production of IL-2 and IL-17 in SLE T cells	58	1.671E-05	0.00094712	13	MHC class II, c-Raf-1, HDAC1, Lck, DNMT3A, c-Fos, ZAP70, CD3, LAT, CaMK IV, ROR-gamma, CD3 zeta, ERK1/2
26	Role of CD8+ Tc1 cells in COPD	44	2.4986E-05	0.00135205	11	Leukocyte elastase, IL-18R1, CCR5, MICA, Leptin receptor, CCL2, MIP-1-alpha, Cathepsin S, T-bet, CXCR6, Granzyme B
27	Cell adhesion_Integrin inside-out signaling in T cells	52	2.5762E-05	0.00135205	12	RASSF5, Lck, CALDAG-GEFI, CCR7, ICAM1, ZAP70, CD3, PIP5KI, CD3 zeta, JAK3, G-protein alpha-i family, CXCR4
28	Immune response_Oncostatin M signaling via MAPK	37	2.8015E-05	0.00141774	10	gp130, c-Raf-1, ERK2 (MAPK1), c-Fos, CCL2, C/EBPbeta, SHP-2, ERK1 (MAPK3), STAT1, ERK1/2
29	Immune response_Antigen presentation by MHC class I: cross-presentation	99	3.7621E-05	0.00183822	17	SEC22B, Cathepsin L, CLEC12A, Dectin-1, IP-30, FCGR3A, Rac2, DAP12, CLEC4C, p67-phox, TRIF (TICAM1), Fc gamma RII alpha, MyD88, HSP70, Cathepsin S, Calreticulin, HSPA1A
30	Defective macrophage-mediated bacterial phagocytosis in COPD	25	4.7261E-05	0.0022323	8	alpha-TAT1, FCGR3A, Tubulin alpha, SR-BI, NRF2, MARCO, Calreticulin, Tubulin (in microtubules)
31	The role of KEAP1/NRF2 pathway in skin sensitization	32	5.006E-05	0.00223631	9	Ubiquitin, Heme oxygenase 1, CCR7, ERK2 (MAPK1), MyD88, HSP70, NRF2, ERK1 (MAPK3), ERK1/2
32	Mast cell migration in asthma	73	5.1568E-05	0.00223631	14	CCL5, CCR1, CCR7, PDGF receptor, CCR3, CCL2, MIP-1-alpha, Adenosine A3 receptor, CXCR6, G-protein alpha-i family, P2Y2, CXCR4, ERK1/2, TGF-beta receptor type I

Supplementary Table S1. (Continued)

#	Maps	Total	p-value	FDR	In Data	Network Objects from Active Data
33	Immune response_IL-15 signaling via MAPK and PI3K cascades	56	5.6556E-05	0.00223631	12	CCL5, IL-2R beta chain, c-Raf-1, Lck, c-Fos, MNK1, TRAF2, SHP-2, T-bet, JAK3, Granzyme B, ERK1/2
34	Immune response_IL-5 signaling via JAK/STAT	56	5.6556E-05	0.00223631	12	RNS2, CISH, DUSP5, Slp76, Cyclin D3, Survivin, Syntenin 1, LPPL, c-Fos, ECP (RNase 3), STAT1, SOCS1
35	Immune response_IFN-gamma signaling via PI3K and NF-kB	56	5.6556E-05	0.00223631	12	Occludin, Fyn, c-Raf-1, Alpha1-globin, MICA, RUNX3, PKR, SHP-2, HLAB, STAT1, ERK1/2, PD-L1
36	Transcription_Role of heterochromatin protein 1 (HP1) family in transcriptional silencing	40	5.8393E-05	0.00223631	10	CtBP, Mi-2, HDAC1, DNMT3A, MeCP2, Histone H3, HP1 alpha, Cyclin E, HP1, Mi-2 alpha
37	IFN-gamma and Th2 cytokines-induced inflammatory signaling in normal and asthmatic airway epithelium	40	5.8393E-05	0.00223631	10	CCL5, STAT6, IL4RA, ICAM1, CCL2, CXCR6, STAT1, ERK1/2, SOCS1, TSLPR
38	Cell adhesion_PLAU signaling	65	6.0209E-05	0.00224516	13	FPRL1, Nucleolin, gp130, c-Raf-1, Survivin, SHP-2, G-protein alpha-i family, F-Actin cytoskeleton, FPR, Paxillin, STAT1, ERK1/2, PDGF-R-beta
39	Immune response _Sialic-acid receptors (Siglecs) signaling	14	6.642E-05	0.00235293	6	Sialoadhesin, CD45, HP, CD33, Lyn, SHP-2
40	Cell cycle_Nucleocytoplasmic transport of CDK/Cyclins	14	6.642E-05	0.00235293	6	Importin (karyopherin)-alpha, Karyopherin beta 1, Cyclin D3, Cyclin E, ERK1 (MAPK3), Cyclin D
41	Development_G-CSF signaling	49	7.3207E-05	0.00253012	11	G-CSF receptor, c-Raf-1, Cyclin D3, Survivin, C/EBPbeta, Lyn, SHP-2, CXCR4, STAT1, ERK1/2, IL8RB
42	B-regulatory cells and tumor cells intercellular interaction	67	8.3766E-05	0.00278492	13	FPRL1, LTB, gp130, STAT6, IL4RA, CD5, CCL2, JAK3, Granzyme B, STAT1, TGF-beta receptor type I, PD-L1, CD40L(TNFSF5)
43	Development_S1P1 receptor signaling via beta-arrestin	34	8.4511E-05	0.00278492	9	c-Raf-1, ERK2 (MAPK1), ERK1 (MAPK3), G-protein alpha-i family, G-protein alpha-i2, GRK2, G-protein alpha-o, ERK1/2, PDGF-R-beta
44	Putative pathways of MHC class I-dependent postsynaptic long-term depression in major depressive disorder	21	0.00010639	0.00342636	7	MHC Class I alpha chain, TAP1 (PSF1), HLAA, PIRB, SHP-2, CD3 zeta, HLAB

Supplementary Table S1. (Continued)

#	Maps	Total	p-value	FDR	In Data	Network Objects from Active Data
45	Chemotaxis_CXCR3-A signaling	69	0.00011491	0.00361842	13	Occludin, c-Raf-1, Lck, ZAP70, CD3, LAT, ROR-gamma, T-bet, Granzyme B, G-protein alpha-i2, GRK2, STAT1, ERK1/2
46	Immune response_Antiviral actions of Interferons	52	0.0001299	0.00400156	11	CIITA, HLAA, IDO1, MxA, OAS3, MHC class II, WARS, PKR, ADAR1, 2'-5'-oligoadenylate synthetase, STAT1
47	Development_EPO-induced Jak-STAT pathway	36	0.00013695	0.004129	9	c-Raf-1, CISH, ERK2 (MAPK1), Lyn, SHP-2, ERK1 (MAPK3), STAT1, ERK1/2, SOCS1
48	Sorafenib-induced inhibition of cell proliferation and angiogenesis in HCC	16	0.00016058	0.00474035	6	c-Raf-1, ERK2 (MAPK1), c-Fos, ERK1 (MAPK3), ERK1/2, PDGF-R-beta
49	Role of tumor-infiltrating B cells in anti-tumor immunity	91	0.00017119	0.00477806	15	IL-18R1, MHC class II, Kappa chain (Ig light chain), STAT6, IL4RA, CD20, CD38, T-bet, CD19, JAK3, G-protein alpha-i family, Granzyme B, CXCR4, STAT1, CD40L(TNFSF5)
50	IL-6 signaling in colorectal cancer	37	0.00017197	0.00477806	9	Ku70, gp130, HDAC1, Cyclin B, Survivin, ICAM1, HSP70, TFF3, Cyclin E
51	Immune response_Regulation of T cell function by CTLA-4	37	0.00017197	0.00477806	9	Fyn, Lck, c-Fos, ZAP70, CD3, LAT, Lyn, SHP-2, CD3 zeta
52	Eosinophil granule protein release in asthma	54	0.0001856	0.00505753	11	FPRL1, Leukocyte elastase, Lactoferrin, RNS2, CRLF2, Cathepsin G, CCR3, ECP (RNase 3), FPR, ERK1/2, TSLPR
53	Development_G-CSF-induced myeloid differentiation	30	0.00019861	0.0053101	8	Lactoferrin, G-CSF receptor, c-Raf-1, SHP-2, C/EBPepsilon, ERK1/2, C/EBPalpha, PERM
54	Immune response_Role of integrins in NK cells cytotoxicity	38	0.00021413	0.00561904	9	Pyk2(FAK2), c-Raf-1, ERK2 (MAPK1), c-Fos, ICAM1, Lyn, SHP-2, ERK1 (MAPK3), Paxillin
55	CHDI_Correlations from Replication data_Cytoskeleton and adhesion module	64	0.0002192	0.00564745	12	RASSF5, Actin cytoskeletal, Fyn, MHC class II, Slp76, CALDAG-GEFI, ICAM1, ZAP70, CD3, Ephrin-B receptors, CXCR4, Paxillin
56	Immune response_IL-12 signaling pathway	74	0.00023951	0.00606054	13	Fyn, Lck, Cyclin D3, Bcl-3, RUNX3, Eomesodermin, CD3, T-bet, IL-2R alpha chain, CD3 zeta, Granzyme B, STAT1, CD40L(TNFSF5)
57	Renal tubulointerstitial injury in Lupus Nephritis	65	0.0002552	0.00634421	12	CCL5, BCMA(TNFRSF17), MHC class II, CCR5, CCR7, ICAM1, CCL2, CMKLR1, TRAF2, RIPK1, CD40L(TNFSF5), SOCS1

Supplementary Table S1. (Continued)

#	Maps	Total	p-value	FDR	In Data	Network Objects from Active Data
58	Immune response_TSLP signaling	39	0.00026454	0.00635345	9	CRLF2, IL7RA, c-Raf-1, CISH, STAT6, CCL2, STAT1, ERK1/2, TSLPR
59	Cooperative action of IFN-gamma and TNF-alpha on astrocytes in multiple sclerosis	39	0.00026454	0.00635345	9	CIITA, MHC class II, G-protein alpha-s, ICAM1, PKR, CCL2, C/EBPbeta, Adenylate cyclase, STAT1
60	Proteolysis_Role of Parkin in the Ubiquitin-Proteasomal Pathway	24	0.0002727	0.00644036	7	Cullin 1, Tubulin beta, Tubulin alpha, SIAH2, UBCH8, HSP70, Cyclin E
61	Apoptosis and survival_Regulation of apoptosis by mitochondrial proteins	106	0.00030064	0.00698374	16	GZMH, BOK, Cathepsin L, CTMP, PP2C, IFI27, Aif, Bik, Bak, Kv1.3, Fis1, Cathepsin S, PUMA, Granzyme B, JSAP1, ERK1/2
62	CCR7 signaling pathways in dendritic cells in allergic contact dermatitis	57	0.00030631	0.00700059	11	RASSF5, Pyk2(FAK2), Actin cytoskeletal, c-Raf-1, Rac2, CALDAG-GEFI, CCR7, ICAM1, G-protein alpha-i family, Paxillin, ERK1/2
63	Development_Transcription regulation of granulocyte development	32	0.00032268	0.0072577	8	Leukocyte elastase, Lactoferrin, G-CSF receptor, CD45, p67-phox, C/EBPepsilon, C/EBPalpha, PERM
64	Inflammatory mechanisms of pancreatic cancerogenesis	67	0.00034243	0.0075096	12	CCL5, C/EBP, MCP, c-Fos, ICAM1, MyD88, CCL2, G-protein alpha-i family, CXCR4, STAT1, CD40L(TNFSF5), IL8RB
65	Neutrophil-derived granule proteins and cytokines in asthma	49	0.00035626	0.0075096	10	Leukocyte elastase, Lactoferrin, RNS2, Rac2, DAP12, Alpha-defensin, ERK1/2, FGR, PERM, IL8RB
66	Immune response_CCR5 signaling in macrophages and T lymphocytes	58	0.00035895	0.0075096	11	CCL5, Pyk2(FAK2), CCR5, Lck, c-Fos, MIP-1-alpha, JAK3, G-protein alpha-i family, Paxillin, STAT1, ERK1/2
67	Cell adhesion_Integrin inside-out signaling in neutrophils	77	0.0003595	0.0075096	13	L-selectin, DAP12, Slp76, CALDAG-GEFI, ERK2 (MAPK1), ICAM1, PIP5KI, Lyn, G-protein alpha-i family, G-protein alpha-i2, FPR, FGR, IL8RB
68	Immune response_IL-2 signaling via JAK/ STAT	25	0.00036038	0.0075096	7	IL-2R beta chain, IL4RA, c-Fos, IL-2R alpha chain, JAK3, Granzyme B, STAT1
69	Role of Bregs in attenuation of T and NK cells mediated anti-tumor immune responses	41	0.00039499	0.00811169	9	L-selectin, 5'-NTD, Lck, CD3, IL-2R alpha chain, CD3 zeta, Granzyme B, TGF-beta receptor type I, PD-L1
70	G-protein signaling_N-RAS regulation pathway	33	0.00040501	0.00819851	8	MHC class II, c-Raf-1, Lck, CALDAG-GEFI, ZAP70, CD3, LAT, CD3 zeta

Supplementary Table S1. (Continued)

#	Maps	Total	p-value	FDR	In Data	Network Objects from Active Data
71	Signal transduction_CXCR4 signaling via second messengers and JAK/STAT	50	0.00042305	0.00844304	10	Ubiquitin, Pyk2(FAK2), G-protein alpha-s, SK4/IK1, JAK3, G-protein alpha-i family, Adenylate cyclase, CXCR4, Paxillin, SOCS1
72	SLE genetic marker-specific pathways in B cells	99	0.00044114	0.00868196	15	BCMA(TNFRSF17), BLK, Fc gamma RII beta, c-Raf-1, Cyclin D3, p67-phox, CalDAG-GEFIII, Bak, CD38, TRAF2, Lyn, Aiolos, IRF5, STAT1, ERK1/2
73	Immune response_CD16 signaling in NK cells	69	0.00045366	0.00880601	12	Actin cytoskeletal, FCGR3A, PIP5K1A, c-Raf-1, Lck, ERK2 (MAPK1), c-Fos, PLA2, ZAP70, LAT, CD3 zeta, ERK1/2
74	CHDI_Correlations from Replication data_Causal network (positive correlations)	79	0.00046517	0.00886546	13	Pyk2(FAK2), MHC class II, Lck, CD45, Slp76, ICAM1, MyD88, HSP70, ZAP70, CD3, LAT, CaMK IV, CXCR4
75	Development_S1P2 and S1P3 receptors in cell proliferation and differentiation	26	0.00046924	0.00886546	7	c-Raf-1, MKL1, c-Fos, Actin, G-protein alpha-i2, ERK1/2, ARHGEF1 (p115RhoGEF)
76	IGF family signaling in colorectal cancer	60	0.00048723	0.0089204	11	GIPC, MNK2(GPRK7), c-Raf-1, ERK2 (MAPK1), c-Fos, MNK1, PTEN, Rad51, ERK1 (MAPK3), ERK1/2, C/EBPalpha
77	Oxidative stress_ROS-mediated MAPK activation via canonical pathways	60	0.00048723	0.0089204	11	GSTP1, Pyk2(FAK2), Fyn, c-Raf-1, ERK2 (MAPK1), TRAF2, SFK, SHP-2, RIPK1, ERK1/2, PDGF-R-beta
78	Immune response_Histamine signaling in dendritic cells	51	0.00049992	0.0089204	10	CCL5, Histamine H4 receptor, c-Raf-1, G-protein alpha-s, ERK2 (MAPK1), CCL2, MIP-1-alpha, ERK1 (MAPK3), G-protein alpha-i family, Adenylate cyclase
79	Development_FGF2 signaling during embryonic stem cell differentiation	34	0.00050362	0.0089204	8	Noggin, Nestin, c-Raf-1, NCAM1, PTCH1, SHP-2, NEFH, ERK1/2
80	NK cells in allergic contact dermatitis	34	0.00050362	0.0089204	8	CIITA, CCL5, MHC class II, CCR5, NCAM1, ICAM1, CCL2, STAT1
81	Development_c-Kit ligand signaling pathway during hemopoiesis	61	0.00056451	0.00956311	11	Pyk2(FAK2), Fyn, c-Raf-1, Lyn, SHP-2, GRAP, CXCR4, Paxillin, STAT1, ERK1/2, SOCS1
82	Signal transduction_MIF signaling pathway	61	0.00056451	0.00956311	11	Heme oxygenase 1, c-Raf-1, ICAM1, ZAP70, NRF2, SFK, Lyn, G-protein alpha-i family, CXCR4, ERK1/2, IL8RB
83	Immune response_IL-9 signaling pathway	61	0.00056451	0.00956311	11	CCL5, c-Raf-1, CISH, Bcl-3, CCL13, CCL2, JAK3, Granzyme B, STAT1, ERK1/2, Fc epsilon RI alpha
84	Development_ACM2 and ACM4 activation of ERK	43	0.0005743	0.00956311	9	ACM4, Pyk2(FAK2), Fyn, c-Raf-1, c-Fos, G-protein alpha-i family, G-protein alpha-i2, G-protein alpha-o, ERK1/2

Supplementary Table S1. (Continued)

#	Maps	Total	p-value	FDR	In Data	Network Objects from Active Data
85	Immune response_IL-7 signaling in B lymphocytes	43	0.0005743	0.00956311	9	Fyn, IL7RA, EBF, c-Raf-1, E2A, Lyn, JAK3, STAT1, ERK1/2
86	Immune response_T cell subsets: cell surface markers	52	0.00058802	0.00956311	10	L-selectin, IL7RA, CCR5, GITR, CCR7, IL4RA, CCR3, CD3, CCR10, IL-2R alpha chain
87	Development_Stimulation of differentiation of mouse embryonic fibroblasts into adipocytes by extracellular factors	71	0.0005939	0.00956311	12	c-Raf-1, G-protein alpha-s, ERK2 (MAPK1), DRIP130, C/EBPbeta, Resistin, SHP-2, ERK1 (MAPK3), Adenylate cyclase, Factor D, ERK1/2, C/EBPalpha
88	Immune response_IL-6 signaling pathway via JAK/STAT	71	0.0005939	0.00956311	12	gp130, Rac2, CISH, c-Fos, ICAM1, CCL2, C/EBPbeta, ROR-gamma, SHP-2, JAK3, STAT1, SOCS1
89	Th2 cytokine- and TNF-alpha-induced inflammatory response in asthmatic airway fibroblasts	35	0.00062081	0.00966697	8	CCL5, STAT6, IL4RA, c-Fos, ICAM1, CCL2, ERK1/2, CD40L(TNFSF5)
90	Development_Extraembryonic differentiation of embryonic stem cells	35	0.00062081	0.00966697	8	HEY1, Noggin, Nestin, NCAM1, CD9, Transferrin, ID4, HNF3-alpha
91	Immune response_Differentiation of natural regulatory T cells	35	0.00062081	0.00966697	8	Ubiquitin, MHC class II, GITR, IL-2R alpha chain, JAK3, TGF-beta receptor type I, CD40L(TNFSF5), TSLPR
92	Immune response_IL-4-induced regulators of cell growth, survival, differentiation and metabolism	62	0.00065176	0.01003847	11	MCM6, ATP6V1B2, CISH, STAT6, IL4RA, SK4/IK1, Cyclin E, Cathepsin V, STAT1, Cyclin D, SOCS1
93	Chemotaxis_CCR1 signaling	53	0.00068857	0.01037978	10	CCL5, CCL23, c-Raf-1, CCR1, CCL13, MIP-1-alpha, G-protein alpha-i family, Adenylate cyclase, G-protein alpha-o, ERK1/2
94	Signal transduction_CXCR4 signaling via MAPKs cascades	53	0.00068857	0.01037978	10	Ubiquitin, c-Raf-1, c-Fos, ZAP70, CD3, CD3 zeta, G-protein alpha-i family, G-protein alpha-i2, CXCR4, ERK1/2
95	Cell cycle_Regulation of G1/S transition (part 2)	28	0.00076482	0.01128908	7	p130, Cyclin D3, c-Fos, DP1, Cyclin E, ERK1/2, Cyclin D
96	Immune response_Innate immune response to RNA viral infection	28	0.00076482	0.01128908	7	IRF7, TANK, TRAF3, MDA-5, RIG-I, RIPK1, LGP2
97	NRF2 regulation of oxidative stress response	54	0.00080288	0.01172864	10	GSTP1, Ubiquitin, Heme oxygenase 1, Actin cytoskeletal, Fyn, c-Raf-1, ERK2 (MAPK1), NRF2, ERK1 (MAPK3), DJ-1

Supplementary Table S1. (Continued)

#	Maps	Total	p-value	FDR	In Data	Network Objects from Active Data
98	Development_Role of G-CSF in hematopoietic stem cell mobilization	21	0.00085236	0.01232449	6	Leukocyte elastase, Cathepsin G, G-CSF receptor, DPP4, ICAM1, CXCR4
99	A shift in alveolar macrophage phenotype in COPD	37	0.00092102	0.01292158	8	HYAL2, ARG1, Heme oxygenase 1, FCGR3A, MHC class II, Adenosine A3 receptor, NRF2, MARCO
100	Immune response_IL-7 signaling in T lymphocytes	37	0.00092102	0.01292158	8	Pyk2(FAK2), GLUT1, Fyn, Lck, STAT6, JAK3, STAT1, SOCS1
101	G-protein signaling_H-RAS regulation pathway	37	0.00092102	0.01292158	8	Angiopoietin 1, GIPC, c-Raf-1, Lck, Beta-1 adrenergic receptor, CalDAG-GEFIII, DOK2, PDGF-R-beta
102	Basophil migration in asthma	55	0.00093234	0.01295223	10	CCL5, FPRL1, CCR1, CCL13, ICAM1, CCR3, CCL2, MIP-1-alpha, G-protein alpha-i family, IL8RB
103	Dual function of Treg cells in cancer development	46	0.00096347	0.0132547	9	5'-NTD, CD45, GITR, ROR-alpha, IFNAR1, ROR-gamma, IL-2R alpha chain, Granzyme B, STAT1

Supplementary Table S2. Enrichment analysis by cell type (BrB ArrayTools and referenced curated gene sets) of the stable 1509 genes (Pattern 0).

GeneList GeneSets	Number of genes in data	P value	Genes in data	Annotation/ gene set collection/ Reference
HAY_BONE_MARROW_NK_CELLS	87	1.27E-16	ZAP70, LPCAT1, CHST2, GFOD1, RASSF5, PXN, CTBP2, ARHGAP9, LITAF, CD247, HDDC2, SLC9A3R1, YPEL1, PPP2R2B, CAP1, TPST2, LAIR2, PLEKHA1, MRPL10, CCDC102A, MAPK1, PITPNC1, KLHDC4, UBE2L6, CD53, CXCR2, FSD1, TINF2, TNFRSF18, USB1, PSMB10, FBXO6, ABHD17A, GNLY, GZMB, OSBPL5, SYTL1, HLA-A, RAP1GAP2, ST6GALNAC6, RUNX3, PYHIN1, APOBEC3H, RAC2, CTDSP1, HSH2D, CD300A, CCL3, BPGM, SSBP4, IL2RB, HCST, IER2, BIN2, ADAM8, CDK2AP2, RNF126, ARHGDIB, SYTL2, HLA-F, UBE2F, NCAM1, ASCL2, NKG7, TADA3, TBX21, ADGRG1, TAP1, EFHD2, SBK1, TKTL1, NMUR1, NCR3, SLC1A7, ZMAT4, EOMES, CIB1, SHISA5, BOK, MYOM2, SPON2, CHST12, DOK2, HLA-C, PPP1R18, BTN3A2, CDC42SE1	C8: cell type signature gene sets (https://www.gsea-msigdb.org/gsea/msigdb/genesets.jsp?collection=C8; Hay, Stuart B., et al. "The Human Cell Atlas bone marrow single-cell interactive web portal." Experimental hematology 68 (2018): 51-61.)
DC4_CD1C– CD141–_Villani_2017	68	8.38E-10	MAFB, FCGR3A, ARHGEF3, WARS, IRAK3, TMEM134, FGR, ALOX5, ISG15, SSH2, SGPL1, LY6E, FTH1, CD300LF, IFITM3, LILRB1, RAB24, SLC2A6, FCGR2A, IFI6, CHST15, SYTL1, RAP1GAP2, PPM1F, GBP4, GBP5, P2RY13, CD300A, SIRPB1, LCP2, BIN2, CMTM7, TYROBP, FPR1, CLEC7A, CCM2, PSTPIP2, SDCBP, CTSS, PIK3IP1, TMEM176B, ADRBK1, CLEC12A, FCN1, APOBEC3A, NCF2, TMTC1, LYN, NPL, TKT, CFD, GPR155, CPPED1, HMOX1, FCGR2C, RALB, NINJ1, MT2A, DDX58, LILRA6, TBC1D8, DENND3, PYGL, IFI30, CDKN1C, DOK2, PTPRC, SLC7A7	DC4 CD1C–CD141–CD11C+CD16+. DC4 cells involved with type I interferon signaling pathway and response to virus. (https://science.sciencemag.org/content/suppl/2017/04/19/356.6335.eaah4573.DC1. Table S1 in: Villani, Alexandra-Chloé, et al. "Single-cell RNA-seq reveals new types of human blood dendritic cells, monocytes, and progenitors." Science 356.6335 (2017).)

Supplementary Table S2. (Continued)

GeneList GeneSets	Number of genes in data	P value	Genes in data	Annotation/Reference
Resting_monocyte_GNF	48	3.51E-08	MAFB, P2RY2, AQP9, CEBPA, IRAK3, BST1, CEBPB, S100A10, SMARCD3, FCGR2A, QPCT, CCR1, MNDA, SIRPA, PLBD1, ASGR2, RNASE2, F5, BPI, CSF3R, MARVELD1, ASGR1, IL1RN, P2RY13, SIRPB1, FOLR3, VSTM1, TMEM176A, FPR2, FPR1, CTSS, TMEM176B, COTL1, FCN1, APOBEC3A, NCF2, RETN, TKT, CFD, CPPED1, NINJ1, CD33, HAL, AGPAT9, PYGL, IFI30, S100P, SLC7A7	3x higher in CD14 monocytes v all other heme subsets (CD19, CD8, CD4, CD56, CD14, BDCA4, CD71, CD105). SignatureDB_annotation_021418 (https://lymphochip.nih.gov/signaturedb/)
HAY_BONE_MARROW_NEUTROPHIL	76	1.46E-07	OAZ1, FAM102B, PLA2G7, AQP9, ACTB, IRAK3, HEXB, ALOX5, BST1, S100A10, EIF4E3, SMARCD3, PGAM1, IL10RB, LAMP2, QPCT, MNDA, GNAI2, SIRPA, PLBD1, ASGR2, PRR13, CREB5, F5, ARHGEF40, GLT1D1, MSRB1, WDFY3, CSF3R, TDRD9, SRD5A1, FOS, ASGR1, BASP1, IL1RN, WLS, P2RY13, GM2A, GPX1, SIRPB1, GSTP1, FOLR3, VSTM1, ARL8A, TMEM176A, TYROBP, FPR2, ATP6V0B, LPGAT1, FPR1, SDCBP, TMEM176B, SLC2A3, CLEC12A, FCN1, NCF2, TSPO, CES1, CLEC4E, STEAP4, SELL, JDP2, TKT, KLF6, SLC24A4, KYNU, TLR8, CD33, CACNA2D4, PELI2, LCP1, LILRA6, CTSZ, PYGL, ACSL1, S100P	C8: cell type signature gene sets (https://www.gsea-msigdb.org/gsea/msigdb/genesets.jsp?collection=C8; Hay, Stuart B., et al. "The Human Cell Atlas bone marrow single-cell interactive web portal." Experimental hematology 68 (2018): 51-61.)
HAY_BONE_MARROW_CD8_T_CELL	20	4.00E-06	GZMH, CLEC2B, KLRG1, TTC16, A2M-AS1, CXCR6, DUSP2, F2R, CD99, ZNF683, TIGIT, GBP5, MIAT, RASAL3, JAKMIP1, CCL5, FYN, HLA-B, PATL2, PTPRC	C8: cell type signature gene sets (https://www.gsea-msigdb.org/gsea/msigdb/genesets.jsp?collection=C8; Hay, Stuart B., et al. "The Human Cell Atlas bone marrow single-cell interactive web portal." Experimental hematology 68 (2018): 51-61.)

Supplementary Table S2. (Continued)

GeneList GeneSets	Number of genes in data	P value	Genes in data	Annotation/Reference
DC3_CD1C_B+_Villani_2017	17	1.50E-05	BST1, MNDA, PLBD1, CREB5, RNASE2, FCER1A, CSF3R, ASGR1, TMEM173, IL1RN, FPR1, SLC2A3, FCN1, CLEC10A, CES1, RETN, LAT2	DC3 CD1C+_B; CD1C+CD163+. DC2 and DC3 correspond to new subdivisions of the CD1C/BDCA-1+ cDC2. (https://science.sciencemag.org/content/suppl/2017/04/19/356.6335.eaah4573.DC1. Table S1 in: Villani, Alexandra-Chloé, et al. "Single-cell RNA-seq reveals new types of human blood dendritic cells, monocytes, and progenitors." Science 356.6335 (2017).)
DC2_CD1C_A+_Villani_2017	22	1.66E-05	BST1, MNDA, C10orf128, PLBD1, CREB5, RNASE2, FCER1A, CSF3R, ASGR1, TMEM173, IL1RN, ADAM8, FPR1, SLC2A3, FCN1, CLEC10A, CES1, RETN, LAT2, CD33, AGPAT9, CLIC2	DC2 CD1C+_A. CD1C++CD32b+. DC2 and DC3 correspond to new subdivisions of the CD1C/BDCA-1+ cDC2. (https://science.sciencemag.org/content/suppl/2017/04/19/356.6335.eaah4573.DC1. Table S1 in: Villani, Alexandra-Chloé, et al. "Single-cell RNA-seq reveals new types of human blood dendritic cells, monocytes, and progenitors." Science 356.6335 (2017).)
HAY_BONE_MARROW_DENDRITIC_CELL	35	9.59E-05	BCL11A, HLA-DQB1, MILR1, HLA-DPA1, PHACTR1, TMEM8B, IRF7, CIITA, FCER1A, HLA-DRB1, PPP1R14B, CLEC4C, SLC35F3, HLA-DMA, TSPAN13, TCF4, ALDH2, HLA-DRB5, LAMP5, SMPD3, SPINT2, DNASE1L3, UNC93B1, C1orf54, ITM2C, GAPT, LILRB4, CLEC10A, KCNK17, HLA-DQA2, CBX6, DUSP5, HLA-DQA1, HLA-DPB1, CLIC2	C8: cell type signature gene sets (https://www.gsea-msigdb.org/gsea/msigdb/genesets.jsp?collection=C8; Hay, Stuart B., et al. "The Human Cell Atlas bone marrow single-cell interactive web portal." Experimental hematology 68 (2018): 51-61.)
Resting_blood_NK_cell_GNF	19	0.000302	LPCAT1, GZMH, YPEL1, LAIR2, FSD1, GNLY, OSBPL5, RUNX3, CD300A, IL2RB, KIR2DS2, NCAM1, NKG7, TBX21, TKTL1, NMUR1, MPPE1, MYOM2, SPON2	3x higher in CD56+ NK cells v all other heme subsets (CD19, CD8, CD4, CD56, CD14, BDCA4, CD71, CD105). SignatureDB_annotation_021418 (https://lymphochip.nih.gov/signaturedb/)

Supplementary Table S2. (Continued)

GeneList GeneSets	Number of genes in data	P value	Genes in data	Annotation/Reference
Thymic_SP_CD4+Tcell_gt_Thymic_DP_Tcell	26	0.0012262	PTCH1, PMEPA1, IFI44L, CCR7, IL4R, STAT1, CHST15, RSAD2, HLA-A, CISH, IL2RB, ANKRD55, SYTL2, GIMAP8, HLA-F, PIK3IP1, SLC2A3, AMICA1, COTL1, ABLIM1, IL6ST, XAF1, SELL, TKTL1, HLA-B, HLA-C	Thymic single positive CD4+ T cells (ave signal > 7log2) 3x greater than Thymic double positive (CD4+,CD8+) T cells (p<0.01). SignatureDB_annotation_021418 (https://lymphochip.nih.gov/signaturedb/)
Regulatory_T_cell_McHugh_Herman_concensus	7	0.0014793	IL2RA, S100A10, LY6E, TNFRSF18, GZMB, CISH, GBP4	SignatureDB_annotation_021418 (https://lymphochip.nih.gov/signaturedb/)
Treg Radens2020	22	0.0020744	FCRL3, IL2RA, ZC2HC1A, HLA-DQB1, LAIR2, CDCA5, F5, MARCH3, NOG, TIGIT, TK1, HLA-DMA, SLC14A1, SIRPB1, CDCA7L, RGPD1, CD38, UTS2, HACD1, CCNB2, CDCA2, HLA-DQA1	(Radens, Caleb M., et al. "Meta-analysis of transcriptomic variation in T-cell populations reveals both variable and consistent signatures of gene expression and splicing." RNA 26.10 (2020): 1320-1333.)

Supplementary Table S3. Enrichment by Pathway Maps of all 3251 genes differentially expressed.

#	Maps	Total	p-value	FDR	In Data	Network Objects from Active Data
1	Development_Thromboxane A2 signaling pathway	50	3.68E-13	2.68E-10	25	PI3K reg class IA (p85), cPKC (conventional), G-protein alpha-12 family, PI3K cat class IA, p70 S6 kinase1, G-protein alpha-s, PKC, PI3K reg class IA, PLC-beta, Tcf(Lef), RAP-1B, G-protein alpha-i family, Adenylate cyclase, GSK3 alpha/beta, CREB1, Beta-catenin, MSK1/2 (RPS6KA5/4), PKA-reg (cAMP-dependent), AKT(PKB), TBXA2R, c-Src, p38 MAPK, PKA-cat (cAMP-dependent), IP3 receptor, G-protein alpha-13
2	Oxidative stress_ROS-induced cellular signaling	108	3.72E-13	2.68E-10	38	Casein kinase II, alpha chains, TfR1, COX-2 (PTGS2), Thioredoxin, PLK3 (CNK), PKC-beta, GSK3 beta, p70 S6 kinase1, RelA (p65 NF-kB subunit), PKC, KEAP1, Cul3/KEAP1/Rbx1 E3 ligase, PTEN, MEKK1(MAP3K1), PKA-cat alpha, HIF1A, IKK-alpha, IL-8, ATM, DLC1 (Dynein LC8a), IRP2, GRP75, SENP1, GADD45 alpha, NOXA, VEGF-A, PKA-reg (cAMP-dependent), NF-kB, SAE2, AKT(PKB), Glutaredoxin 1, Cytochrome c, NF-kB p50/p65, p300, FASN, c-Src, p38 MAPK, NFKBIA
3	Cell cycle_Influence of Ras and Rho proteins on G1/S Transition	53	1.98E-12	9.49E-10	25	STAT3, MLCP (cat), MLCP (reg), PI3K cat class IA, GSK3 beta, p70 S6 kinase1, Rb protein, RelA (p65 NF-kB subunit), PI3K reg class IA, DIA1, IKK-alpha, Tob1, MRLC, CDC42, AKT(PKB), NF-kB p50/p65, PDK (PDPK1), RalA, p27KIP1, c-Myc, NFKBIA, MLCK, Rac1, MEK4(MAP2K4), CDK6
4	Signal transduction_Calcium-mediated signaling	72	4.73E-12	1.61E-09	29	MLCP (cat), COX-2 (PTGS2), MLCP (reg), I-kB, PKC-beta, ROCK, cPKC (conventional), MARK2, RelA (p65 NF-kB subunit), Calcineurin A (catalytic), Calmodulin, PKC, ASK1 (MAP3K5), CaMKK, 14-3-3, CABIN1, CREB1, HDAC4, MEF2, NF-kB, HDAC5, AKT(PKB), p300, p38 MAPK, CaMKK2, Rac1, IP3 receptor, MEK4(MAP2K4), CARD11
5	Immune response_Platelet activating factor/ PTAFR pathway signaling	55	5.58E-12	1.61E-09	25	STAT3, JAK2, PI3K cat class IA, Calcineurin A (catalytic), PI3K reg class IA, PLC-beta, NF-AT1(NFATC2), IKK-alpha, ASK1 (MAP3K5), G-protein alpha-i family, Adenylate cyclase, G-protein beta/gamma, STAT5, PKA-reg (cAMP-dependent), NF-kB, AKT(PKB), NF-kB p50/p65, Beta-arrestin1, c-Src, Calcineurin B (regulatory), p38 MAPK, PKA-cat (cAMP-dependent), NFKBIA, NF-AT, IP3 receptor
6	Chemotaxis_Lysophosphatidic acid signaling via GPCRs	129	9.25E-12	2.09E-09	40	LPAR2, MLCP (reg), ROCK, PI3K reg class IA (p85), cPKC (conventional), G-protein alpha-12 family, GSK3 beta, Caspase-7, p70 S6 kinase1, PKC, PLC-beta, FKHR, Vinculin, PLEKHG2, DIA1, Tcf(Lef), IL-8, LPAR6, alpha-V/beta-3 integrin, G-protein alpha-i family, Caspase-3, ROCK1, Bcl-2, FasR(CD95), MKL2, PRK1, CREB1, Beta-catenin, G-protein beta/gamma, Rho GTPase, CDC42, N-CoR, HDAC7, AKT(PKB), PDK (PDPK1), c-Src, p38 MAPK, Rac1, IP3 receptor, MEK4(MAP2K4)

Supplementary Table S3. (Continued)

#	Maps	Total	p-value	FDR	In Data	Network Objects from Active Data
7	Development_VEGF signaling via VEGFR2 - generic cascades	93	1.02E-11	2.09E-09	33	COX-2 (PTGS2), I-kB, PKC-beta, COX-1 (PTGS1), PI3K cat class IA, GSK3 beta, Calcineurin A (catalytic), Calmodulin, PKC, NCK1, PI3K reg class IA, MEKK1(MAP3K1), Vinculin, alpha-V/beta-3 integrin, ROCK1, eIF4E, eNOS, CREB1, Beta-catenin, VEGF-A, CDC42, AKT(PKB), HSP90, NF-kB p50/p65, PDK (PDPK1), c-Src, Calcineurin B (regulatory), p38 MAPK, p90Rsk, PLAU (UPA), MLCK, Rac1, IP3 receptor
8	CHDI_Correlations from Replication data_Causal network (positive correlations)	79	1.21E-11	2.18E-09	30	I-kB, ROCK, PI3K reg class IA (p85), PI3K cat class IA, Calcineurin A (catalytic), Calmodulin, NF-AT1(NFATC2), CD44, IKK-alpha, HIP1, NR2, Caspase-3, PSMC2, CREB1, CD28, IP3R1, G-protein beta/gamma, MSK1/2 (RPS6KA5/4), IRAK1/2, HDAC7, MEF2, NF-kB, AKT(PKB), HSP70, Calcineurin B (regulatory), NR2A, p38 MAPK, CXCR4, IP3 receptor, MEK4(MAP2K4)
9	Signal transduction_mTORC1 downstream signaling	60	5.84E-11	9.34E-09	25	STAT3, PDK1, Rictor, p70 S6 kinases, GSK3 beta, p70 S6 kinase1, LIPIN1, HIF1A, MTHFD2, PDCD4, RPS6, eIF4A, ACSL3, eIF4E, ATF-4, SGK1, VEGF-A, Cytochrome c, CLIP170, ULK2, p27KIP1, TFEB, YY1, SIN1, ULK1
10	Signal transduction_Adenosine A2B receptor signaling pathway	71	1.25E-10	1.79E-08	27	PDK1, RAP-1A, PI3K reg class IA (p85), PI3K cat class IA, GSK3 beta, Calcineurin A (catalytic), G-protein alpha-s, PKC, NF-kB1 (p105), PLC-beta, HIF1A, RAP-1B, Adenylate cyclase, eNOS, PER2, CREB1, JAB1, G-protein beta/gamma, VEGF-A, PKA-reg (cAMP-dependent), AKT(PKB), NF-kB p50/p65, PDK (PDPK1), Calcineurin B (regulatory), p38 MAPK, PKA-cat (cAMP-dependent), IP3 receptor
11	Immune response_B cell antigen receptor (BCR) pathway	110	3.68E-10	4.43E-08	34	PKC-beta, PI3K reg class IA (p85), GSK3 beta, p70 S6 kinase1, Rb protein, RelA (p65 NF-kB subunit), Calcineurin A (catalytic), Calmodulin, NCK1, CD79A, NF-kB1 (p50), K-RAS, MEKK1(MAP3K1), FKHR, IKK-alpha, CalDAG-GEFII, PIP5KIII, GSK3 alpha/beta, CDC42, PP2A catalytic, NF-kB, AKT(PKB), DAPP1, NF-kB p50/p65, PDK (PDPK1), Calcineurin B (regulatory), p38 MAPK, PKC-beta2, NFKBIA, PI3K cat class IA (p110-delta), Rac1, IP3 receptor, CARD11, CDK6
12	Signal transduction_PKA signaling	51	3.7E-10	4.43E-08	22	LBC, G-protein alpha-12 family, G-protein alpha-s, AKAP11, PDE4D, PDE3B, PKA-cat alpha, G-protein alpha-i family, Adenylate cyclase, GSK3 alpha/beta, CREB1, PP2A regulatory, PKA-reg type II (cAMP-dependent), PKA-reg (cAMP-dependent), p90RSK1, PDK (PDPK1), AKAP7 gamma, PKI, PKA-cat (cAMP-dependent), AKAP8, NFKBIA, G-protein alpha-13

Supplementary Table S3. (Continued)

#	Maps	Total	p-value	FDR	In Data	Network Objects from Active Data
13	Signal transduction_MIF signaling pathway	61	5.87E-10	6.5E-08	24	COX-2 (PTGS2), PI3K cat class IA, GSK3 beta, RelA (p65 NF-kB subunit), CD74, PI3K reg class IA, PLC-beta, SPPL2a, CD44, IL-8, G-protein alpha-i family, TLR4, ACKR3, Bcl-2, G-protein beta/gamma, NF-kB, AKT(PKB), Beta-arrestin1, PDK (PDPK1), PRDX1, CD74-ICD, SFK, c-Src, CXCR4
14	K-RAS signaling in pancreatic cancer	44	7.5E-10	7.71E-08	20	RelA (p65 NF-kB subunit), K-RAS, PTEN, MEKK1(MAP3K1), IKK-alpha, IL-8, Bcl-2, GSK3 alpha/beta, MIRK, VEGF-A, AKT(PKB), NF-kB p50/p65, ATR, Thrombospondin 1, PDK (PDPK1), RalA, c-Myc, NFKBIA, PLAU (UPA), Rac1
15	Development_Role of HDAC and calcium/calmodulin-dependent kinase (CaMK) in control of skeletal myogenesis	53	9.06E-10	8.7E-08	22	CARM1, PI3K cat class IA, p70 S6 kinase1, Calcineurin A (catalytic), Calmodulin, p38beta (MAPK11), PI3K reg class IA, NF-AT1(NFATC2), PCAF, CaMKK, MEF2C, 14-3-3, MEF2A, HDAC4, HDAC7, MEF2, HDAC5, AKT(PKB), p300, IGF-1, PDK (PDPK1), Calcineurin B (regulatory)
16	Apoptosis and survival_NGF/ TrkA PI3K-mediated signaling	77	1.05E-09	8.91E-08	27	MLCP (cat), TRIO, RAP-1A, MLCP (reg), ROCK, PI3K reg class IA (p85), PI3K cat class IA, GSK3 beta, PARD3, FOXO4, p70 S6 kinase1, Calcineurin A (catalytic), Calmodulin, FKHR, N-WASP, Destrin, MRLC, Bcl-2, CREB1, CDC42, VAV-3, AKT(PKB), PDK (PDPK1), c-Src, Calcineurin B (regulatory), Rac1, Tubulin (in microtubules)
17	Glucocorticoids-mediated inhibition of pro-constrictory and pro-inflammatory signaling in airway smooth muscle cells	49	1.08E-09	8.91E-08	21	MLCP (cat), COX-2 (PTGS2), MLCP (reg), RelA (p65 NF-kB subunit), GCR Beta, G-protein alpha-s, PDE4D, GCR, MRLC, IFN-gamma, MKP-1, GCR Alpha, Beta-2 adrenergic receptor, NF-kB, NF-kB p50/p65, PLA2, p300, p38 MAPK, NFKBIA, Neurokinin-2 receptor, Histone H4
18	Cytoskeleton remodeling_Regulation of actin cytoskeleton organization by the kinase effectors of Rho GTPases	58	1.11E-09	8.91E-08	23	MLCP (cat), MLCP (reg), ROCK, WRCH-1, DMPK, Rac1-related, Cdc42 subfamily, Vinculin, ERM proteins, Destrin, Spectrin, MRLC, PRK1, ARPC1B, Cortactin, CDC42, Alpha-actinin, TC10, MyHC, Rac3, MLCK, Rac1, MRCK

Supplementary Table S3. (Continued)

#	Maps	Total	p-value	FDR	In Data	Network Objects from Active Data
19	Development_GM-CSF signaling	50	1.69E-09	1.22E-07	21	STAT3, I-kB, PKC-beta, STAT5A, JAK2, PI3K reg class IA (p85), PI3K cat class IA, Hck, PI3K reg class IA (p85-alpha), GM-CSF receptor, IKK-alpha, CSF2RB, CSF2RA, Caspase-3, Bcl-2, CREB1, STAT5, NF-kB, AKT(PKB), c-Myc, Mcl-1
20	Apoptosis and survival_HTR1A signaling	50	1.69E-09	1.22E-07	21	STAT3, I-kB, JAK2, Calmodulin, IKK-alpha, G-protein alpha-i family, Adenylate cyclase, XIAP, Caspase-3, Bcl-2, G-protein beta/gamma, PP2A regulatory, PKA-reg (cAMP-dependent), PP2A catalytic, NF-kB, AKT(PKB), Cytochrome c, NF-kB p50/p65, PDK (PDPK1), c-Src, PKA-cat (cAMP-dependent)
21	Immune response_TLR2 and TLR4 signaling pathways	69	1.99E-09	1.36E-07	25	COX-2 (PTGS2), I-kB, PI3K reg class IA (p85), PI3K cat class IA, Hck, Pellino 1, IRAK4, NF-kB1 (p105), IKK-alpha, IL-8, TLR4, IRAK1, MD-2, IRAK2, CREB1, MSK1/2 (RPS6KA5/4), E2N(UBC13), NF-kB, TAB2, AKT(PKB), PDK (PDPK1), p38 MAPK, p90Rsk, Rac1, MEK4(MAP2K4)
22	PGE2 pathways in cancer	55	2.11E-09	1.38E-07	22	COX-2 (PTGS2), PGE2R4, COX-1 (PTGS1), GSK3 beta, G-protein alpha-s, HIF1A, Tcf(Lef), G-protein alpha-i family, Lef-1, Adenylate cyclase, CREB1, Beta-catenin, G-protein beta/gamma, VEGF-A, PKA-reg (cAMP-dependent), AKT(PKB), Beta-arrestin1, PDK (PDPK1), c-Src, c-Myc, Axin, PKA-cat (cAMP-dependent)
23	Apoptosis and survival_Endoplasmic reticulum stress response pathway	56	3.16E-09	1.98E-07	22	I-kB, Caspase-7, XBP1, PP1-cat, ASK1 (MAP3K5), tBid, PP1-cat alpha, Bcl-2, ATF-4, IP3R1, GADD34, ATF-6 alpha (90kDa), eIF2S1, Cytochrome c, NF-kB p50/p65, Calpain 2(m), S1P, ATF-6 alpha (50kDa), Derlin-2, HERP, MEK4(MAP2K4), Bid
24	Apoptosis and survival_TNFR1 signaling pathway	43	3.61E-09	2.16E-07	19	I-kB, c-IAP2, Caspase-10, Caspase-7, MEKK1(MAP3K1), c-IAP1, tBid, XIAP, RAIDD, Caspase-3, Bcl-2, jBid, NF-kB, Cytochrome c, Caspase-8, MKK7 (MAP2K7), ERAP1, MEK4(MAP2K4), Bid
25	Signal transduction_Adenosine A3 receptor signaling pathway	48	4.89E-09	2.82E-07	20	STAT3, PI3K reg class IA (p85), PI3K cat class IA, GSK3 beta, RelA (p65 NF-kB subunit), PKC, PLC-beta, HIF1A, G-protein alpha-i family, Adenylate cyclase, Bcl-2, CREB1, G-protein beta/gamma, Rho GTPase, VEGF-A, AKT(PKB), PDK (PDPK1), p38 MAPK, NFKBIA, IP3 receptor
26	Apoptosis and survival_Role of PKR in stress-induced apoptosis	53	6.01E-09	3.21E-07	21	PACT, I-kB, Caspase-7, IKK-alpha, TLR4, Caspase-3, IFN-gamma, FasR(CD95), eIF4E, PPP2R5A, ATF-4, PP2A regulatory, PP2A catalytic, eIF2S1, NF-kB, MSK2, TAB2, NF-kB p50/p65, Caspase-8, c-Myc, NFKBIA

Supplementary Table S3. (Continued)

#	Maps	Total	p-value	FDR	In Data	Network Objects from Active Data
27	Immune response_HMGB1/RAGE signaling pathway	53	6.01E-09	3.21E-07	21	I-kB, PI3K reg class IA (p85), PI3K cat class IA, PI3K reg class IA (p85-alpha), K-RAS, DIA1, IL-8, TLR4, MEF2C, CREB1, MEF2A, CDC42, p90RSK2(RPS6KA3), p90RSK1, NF-kB, AKT(PKB), c-Src, p38 MAPK, NFKBIA, Rac1, RAGE
28	Development_Negative regulation of WNT/Beta-catenin signaling in the nucleus	89	8.44E-09	4.34E-07	28	NF-AT5, TBL1X, Casein kinase I delta, GSK3 beta, Calcineurin A (catalytic), NARF, BACH1, Oct-3/4, Tcf(Lef), WNT, Lef-1, eNOS, 14-3-3, Jade-1, SENP1, Beta-catenin, P15RS, VEGF-A, TRIM33, TBLR1, CHD8, TAB2, TLE, PJA2, HIC5, Kaiso, Axin, Histone H1
29	MAPK-independent proliferation of normal and asthmatic smooth muscle cells	64	1.05E-08	5.23E-07	23	STAT3, I-kB, JAK2, PI3K reg class IA (p85), PI3K cat class IA, p70 S6 kinase1, Rb protein, PI3K reg class IA (p85-alpha), PI3K reg class IA, Endothelin-1, IKK-alpha, G-protein alpha-i family, PDGF-A, PDGF-C, G-protein beta/gamma, NF-kB, AKT(PKB), TBXA2R, PDK (PDPK1), c-Src, p27KIP1, c-Myc, Rac1
30	Role of red blood cell adhesion to endothelium in vaso-occlusion in Sickle cell disease	37	1.17E-08	5.56E-07	17	TfR1, RAP-1A, ITGA4, alpha-IIb/beta-3 integrin, CD44, IL-8, alpha-V/beta-3 integrin, RAP-1B, G-protein alpha-i family, PKA-reg (cAMP-dependent), von Willebrand factor, Beta-2 adrenergic receptor, Thrombospondin 1, c-Src, P-selectin, PKA-cat (cAMP-dependent), CD47
31	IL-2 as a growth factor for T cells in multiple sclerosis	33	1.2E-08	5.56E-07	16	PCNA, STAT5A, PI3K reg class IA (p85), PI3K cat class IA, GSK3 beta, p70 S6 kinase1, Rb protein, FKHR, Bcl-2, c-Myb, CREB1, NF-kB, AKT(PKB), PDK (PDPK1), p27KIP1, c-Myc
32	Signal transduction_CXCR4 signaling via PI3K cascade	46	1.41E-08	6.28E-07	19	PI3K reg class IA (p85), PI3K cat class IA, GSK3 beta, p70 S6 kinase1, RelA (p65 NF-kB subunit), NF-kB1 (p50), FKHR, G-protein alpha-i family, Caspase-3, eNOS, Beta-catenin, G-protein beta/gamma, SGK1, VEGF-A, AKT(PKB), PDK (PDPK1), NFKBIA, CXCR4, Rac1
33	Signal transduction_PDGF signaling via PI3K/AKT and NFkB pathways	70	1.48E-08	6.28E-07	24	PDK1, PI3K reg class IA (p85), GSK3 beta, RelA (p65 NF-kB subunit), Calmodulin, K-RAS, PTEN, FKHR, HXK2, HIF1A, PDGF-A, PDGF-C, Beta-catenin, SGK1, NF-kB, AKT(PKB), NF-kB p50/p65, PDK (PDPK1), DDX5, c-Src, p27KIP1, c-Myc, NFKBIA, Rac1

Supplementary Table S3. (Continued)

#	Maps	Total	p-value	FDR	In Data	Network Objects from Active Data
34	Ovarian cancer (main signaling cascades)	65	1.48E-08	6.28E-07	23	LPAR2, I-kB, PI3K cat class IA, GSK3 beta, NCOA4 (ARA70), PI3K reg class IA, K-RAS, Endothelin-1, PTEN, MEKK1(MAP3K1), IKK-alpha, Tcf(Lef), G-protein alpha-i family, CREB1, Beta-catenin, G-protein beta/gamma, PKA-reg (cAMP-dependent), NF-kB, AKT(PKB), c-Src, c-Myc, PKA-cat (cAMP-dependent), PLAU (UPA)
35	Regulation of Beta-catenin activity in colorectal cancer	56	1.92E-08	7.69E-07	21	LPAR2, PGE2R4, PKC-beta, PI3K reg class IA (p85), PI3K cat class IA, GSK3 beta, G-protein alpha-s, Calmodulin, PLC-beta, PTEN, IKK-alpha, PDCD4, MAML1, Adenylate cyclase, Beta-catenin, PKA-reg (cAMP-dependent), AKT(PKB), p300, Axin, PKA-cat (cAMP-dependent), IP3 receptor
36	Development_Positive regulation of WNT/Beta-catenin signaling in the cytoplasm	76	1.93E-08	7.69E-07	25	Casein kinase II, alpha chains, COX-2 (PTGS2), GSKIP, TBL1X, BIG1, BIG2, HIPK2, USP25, Tcf(Lef), UBE2B, WNT, PP1-cat, 14-3-3, GSK3 alpha/beta, Beta-catenin, HSP105, PKA-reg type II (cAMP-dependent), TBLR1, PP2A catalytic, AKT(PKB), Axin, PKA-cat (cAMP-dependent), ZBED3, Rac1, DOCK4
37	Role of CNTF and LIF in regulation of oligodendrocyte development in multiple sclerosis	30	2.02E-08	7.69E-07	15	STAT3, JAK2, c-IAP2, PI3K cat class IA, RelA (p65 NF-kB subunit), IMPA1, PI3K reg class IA, IKK-alpha, Caspase-3, IFN-gamma, CLIC4, 14-3-3, AKT(PKB), PDK (PDPK1), NFKBIA
38	Blood coagulation_GPCRs in platelet aggregation	71	2.03E-08	7.69E-07	24	MLCP (cat), RAP-1A, MLCP (reg), NRIF3, ROCK, G-protein alpha-s, Prostacyclin receptor, alpha-IIb/beta-3 integrin, ITGA2B, ITGB3, RAP-1B, G-protein alpha-i family, Adenylate cyclase, MRLC, P2Y1, IP3R1, G-protein beta/gamma, PKA-reg (cAMP-dependent), G-protein alpha-z, TBXA2R, PKA-cat (cAMP-dependent), IP3 receptor, G-protein alpha-13, PLC-beta2
39	Signal transduction_Additional pathways of NF-kB activation (in the cytoplasm)	52	2.52E-08	9.09E-07	20	I-kB, PKC-beta, PI3K cat class IA, RelA (p65 NF-kB subunit), PI3K reg class IA, NF-kB1 (p50), MEKK1(MAP3K1), PKC-lambda/iota, PKA-cat alpha, IKK-alpha, Adenylate cyclase, Casein kinase II, alpha chain (CSNK2A1), PKA-reg (cAMP-dependent), p90RSK1, AKT(PKB), NF-kB p50/p65, PDK (PDPK1), c-Src, NFKBIA, CDK6
40	G-protein signaling_Proinsulin C-peptide signaling	52	2.52E-08	9.09E-07	20	COX-2 (PTGS2), I-kB, PI3K reg class IA (p85), PI3K cat class IA, ATP1A1, ATP1B1, PI3K reg class IA (p85-alpha), PI3K reg class IA, PLC-beta, IKK-alpha, G-protein alpha-i family, Bcl-2, eNOS, G-protein beta/gamma, NF-kB, AKT(PKB), NF-kB p50/p65, PDK (PDPK1), c-Src, IP3 receptor

Supplementary Table S3. (Continued)

#	Maps	Total	p-value	FDR	In Data	Network Objects from Active Data
41	Development_Thrombopoietin signaling via ERK1/2 and PI3K	67	2.87E-08	9.85E-07	23	PDK1, RAP-1A, JAK2, PI3K reg class IA (p85), PI3K cat class IA, GSK3 beta, p70 S6 kinase1, RelA (p65 NF-kB subunit), ITGA2B, HIF1A, PP1-cat, Glycoprotein VI, c-MPL, CREB1, VEGF-A, AKT(PKB), FLI1, PDK (PDPK1), TAL1, p27KIP1, c-Myc, GP-IX, p38 MAPK
42	Signal transduction_Adenosine A1 receptor signaling pathway	62	2.87E-08	9.85E-07	22	STAT3, I-kB, PI3K reg class IA (p85), Calmodulin, PKC, PLC-beta, ASK1 (MAP3K5), G-protein alpha-i family, Adenylate cyclase, CREB1, G-protein beta/gamma, PKA-reg (cAMP-dependent), PP2A catalytic, NF-kB, AKT(PKB), PDK (PDPK1), SFK, c-Src, p38 MAPK, PKA-cat (cAMP-dependent), IP3 receptor, PLC-beta2
43	Immune response_IL-4 signaling pathway	94	3.22E-08	1.08E-06	28	STAT3, JAK2, p70 S6 kinase1, PI3K reg class IA (p85-alpha), Tubulin alpha, p38beta (MAPK11), PKC, MEKK1(MAP3K1), FKHR, PDE3B, IKK-alpha, Bcl-2, GSK3 alpha/beta, CREB1, STAT5, CDC42, p90RSK1, c-Fes, AKT(PKB), NF-kB p50/p65, PDK (PDPK1), PDE4, Fra-2, p38 MAPK, NFKBIA, PI3K cat class IA (p110-delta), Rac1, IP3 receptor
44	Regulation and signaling of HGF receptor (Met) and MSP receptor (RON) in lung cancer	73	3.75E-08	1.23E-06	24	STAT3, COX-2 (PTGS2), PI3K reg class IA (p85), PI3K cat class IA, GSK3 beta, p70 S6 kinase1, Rb protein, NCK1, K-RAS, MEKK1(MAP3K1), HIF1A, PDGF-A, CREB1, Beta-catenin, VEGF-A, CDC42, AKT(PKB), Thrombospondin 1, PDK (PDPK1), c-Src, PKC-beta2, p90Rsk, Rac1, Gamma adducin
45	Signal transduction_IGF-1 receptor signaling pathway	58	3.94E-08	1.25E-06	21	STAT3, I-kB, JAK2, PI3K reg class IA (p85), PI3K cat class IA, GSK3 beta, p70 S6 kinase1, RelA (p65 NF-kB subunit), FKHR, RPS6, HMDH, ASK1 (MAP3K5), Bcl-2, eIF4E, AKT(PKB), IGF-1, MKK7 (MAP2K7), PDK (PDPK1), FASN, ACSA, MEK4(MAP2K4)
46	Neuroprotective action of lithium	63	4.02E-08	1.25E-06	22	Thioredoxin, GSK3 beta, Calcineurin A (catalytic), IPP-2, WNT, NR2, PP1-cat, ASK1 (MAP3K5), Caspase-3, Bcl-2, CREB1, Beta-catenin, VEGF-A, p300, HSP70, MKK7 (MAP2K7), c-Src, Calcineurin B (regulatory), NR2A, p38 MAPK, Axin, MEK4(MAP2K4)
47	Leptin signaling in colorectal cancer	44	4.07E-08	1.25E-06	18	STAT3, JAK2, PI3K cat class IA, GSK3 beta, p70 S6 kinase1, RelA (p65 NF-kB subunit), PI3K reg class IA, PTEN, IKK-alpha, IL-8, Beta-catenin, VEGF-A, CDC42, AKT(PKB), c-Src, c-Myc, NFKBIA, Rac1

Supplementary Table S3. (Continued)

#	Maps	Total	p-value	FDR	In Data	Network Objects from Active Data
48	Inhibition of apoptosis in pancreatic cancer	59	5.57E-08	1.67E-06	21	STAT3, COX-2 (PTGS2), JAK2, PI3K reg class IA (p85), PI3K cat class IA, RelA (p65 NF-kB subunit), ALOX12, K-RAS, PTEN, IKK-alpha, G-protein alpha-i family, Casein kinase II, alpha chain (CSNK2A1), Bcl-2, 14-3-3, STAT5, VEGF-A, AKT(PKB), Cytochrome c, IGF-1, Mcl-1, NFKBIA
49	Immune response_IL-3 signaling via ERK and PI3K	102	5.77E-08	1.7E-06	29	RAP-1A, JAK2, p70 S6 kinases, PI3K reg class IA (p85), cPKC (conventional), PI3K cat class IA, GSK3 beta, Calcineurin A (catalytic), Calmodulin, CSF2RB, RPS6, Bcl-2, GSK3 alpha/beta, CREB1, STAT5, CDC42, AKT(PKB), PDK (PDPK1), GATA-2, PDE4, p27KIP1, Mcl-1, Calcineurin B (regulatory), PKA-cat (cAMP-dependent), p90Rsk, PI3K cat class IA (p110-delta), LPCAT2, Rac1, IP3 receptor
50	Development_Role of CNTF and LIF in regulation of oligodendrocyte development	28	6.06E-08	1.75E-06	14	STAT3, JAK2, c-IAP2, PI3K cat class IA, RelA (p65 NF-kB subunit), IMPA1, PI3K reg class IA, IKK-alpha, Caspase-3, CLIC4, 14-3-3, AKT(PKB), PDK (PDPK1), NFKBIA
51	Immune response_Function of MEF2 in T lymphocytes	50	7.09E-08	2E-06	19	CARM1, Calcineurin A (catalytic), Calmodulin, p38beta (MAPK11), Sin3A, NF-AT1(NFATC2), PCAF, CaMKK, MEF2C, 14-3-3, CABIN1, MEF2A, HDAC4, HDAC7, MEF2, HDAC5, Calcineurin A (beta), p300, IP3 receptor
52	Tau pathology in Alzheimer disease	55	7.63E-08	2.04E-06	20	Casein kinase I delta, GSK3 beta, MARK2, Caspase-7, Calcineurin A (catalytic), SYVN1, p38beta (MAPK11), PKC, NR2, Caspase-3, DYRK1a, GSK3 alpha/beta, OGT (GlcNAc transferase), PP2C, PP2A catalytic, Caspase-8, Calpain 2(m), p38 MAPK, MAP1LC3A, Tubulin (in microtubules)
53	Main growth factor signaling cascades in multiple myeloma cells	41	7.65E-08	2.04E-06	17	STAT3, I-kB, PI3K reg class IA (p85), PI3K cat class IA, PI3K reg class IA (p85-alpha), PI3K reg class IA, K-RAS, PTEN, FKHR, HIF1A, IKK-alpha, GSK3 alpha/beta, VEGF-A, NF-kB, AKT(PKB), IGF-1, PDK (PDPK1)

Supplementary Table S3. (Continued)

#	Maps	Total	p-value	FDR	In Data	Network Objects from Active Data
54	Cell adhesion_PLAU signaling	65	7.66E-08	2.04E-06	22	Casein kinase II, alpha chains, STAT3, Casein kinase II, beta chain (Phosvitin), c-IAP2, ROCK, PI3K cat class IA, PI3K reg class IA (p85-alpha), IKK-alpha, c-IAP1, alpha-V/beta-3 integrin, G-protein alpha-i family, XIAP, MRLC, CDC42, NF-kB, AKT(PKB), MYLK1, c-Src, alpha-V/beta-5 integrin, PLAU (UPA), MLCK, Rac1
55	DNA damage_p53 activation by DNA damage	60	7.8E-08	2.04E-06	21	PLK3 (CNK), RelA (p65 NF-kB subunit), MEKK1(MAP3K1), HIPK2, PCAF, ATM, Bcl-2, 14-3-3, CABIN1, GADD45 alpha, NOXA, PP2A regulatory, PP2A catalytic, ATR, p300, P53DINP1a, 14-3-3 theta, p38 MAPK, DBC1, DYRK2, MEK4(MAP2K4)
56	Development_The role of GDNF ligand family/ RET receptor in cell survival, growth and proliferation	92	7.98E-08	2.05E-06	27	STAT3, RAP-1A, ROCK, PI3K reg class IA (p85), PI3K cat class IA, Calmodulin, CREM (activators), NCK1, MEKK1(MAP3K1), HIF1A, IKK-alpha, alpha-V/beta-3 integrin, XIAP, ATF-1, Bcl-2, CREB1, VEGF-A, CDC42, p90RSK2(RPS6KA3), NF-kB, AKT(PKB), PDK (PDPK1), c-Src, NFKBIA, Rac1, IP3 receptor, MEK4(MAP2K4)
57	G protein-coupled receptors signaling in lung cancer	76	8.98E-08	2.27E-06	24	STAT3, PGE2R4, I-kB, G-protein alpha-12 family, p70 S6 kinase1, RelA (p65 NF-kB subunit), G-protein alpha-s, Calmodulin, Endothelin-1, CD44, IKK-alpha, IL-8, alpha-V/beta-3 integrin, G-protein alpha-i family, G-protein beta/gamma, Galpha(i)-specific peptide GPCRs, VEGF-A, PKA-reg (cAMP-dependent), Galpha(q)-specific peptide GPCRs, AKT(PKB), PDK (PDPK1), c-Src, PKA-cat (cAMP-dependent), CXCR4
58	Immune response_IL-3 signaling via JAK/STAT, p38, JNK and NF-kB	93	1.02E-07	2.54E-06	27	CD69, Ephrin-B1, STAT3, I-kB, STAT5A, JAK2, PI3K cat class IA, SRP9, IKK-alpha, CSF2RB, XBP1, Bcl-2, MKP-1, 14-3-3 gamma, STAT5, NF-kB, AKT(PKB), NF-kB p50/p65, MKK7 (MAP2K7), c-Src, c-Myc, Mcl-1, P-selectin, p38 MAPK, SPECC1, Rac1, MEK4(MAP2K4)
59	Signal transduction_Non-canonical WNT5A signaling	82	1.06E-07	2.56E-06	25	MLCP (cat), MLCP (reg), ROCK, cPKC (conventional), Casein kinase I delta, Calcineurin A (catalytic), Calmodulin, CCDC88C, PLC-beta, WNT3A, PKC-lambda/iota, IL-8, G-protein alpha-i family, Lef-1, Beta-catenin, G-protein beta/gamma, CDC42, TAB2, Villin 1, RYK, Calcineurin B (regulatory), p38 MAPK, NF-AT, Rac1, IP3 receptor
60	Proteolysis_Putative SUMO-1 pathway	29	1.07E-07	2.56E-06	14	GCR, SUMO-1, c-Myb, FasR(CD95), SP3, SENP1, UBE2E3, HSF2, NF-kB, SAE2, TOP2, SP100, RanBP2, NFKBIA

Supplementary Table S3. (Continued)

#	Maps	Total	p-value	FDR	In Data	Network Objects from Active Data
61	Development_PEDF signaling	99	1.1E-07	2.6E-06	28	STAT3, NF-kB p50/p50, c-IAP2, PI3K cat class IA, GSK3 beta, RelA (p65 NF-kB subunit), PI3K reg class IA, NF-kB1 (p50), IKK-alpha, c-IAP1, tBid, RAIDD, Caspase-3, Bcl-2, CREB1, Beta-catenin, p90RSK2(RPS6KA3), N-CoR, NF-kB, AKT(PKB), NF-kB p50/p65, PDK (PDPK1), Fra-2, p38 MAPK, PKA-cat (cAMP-dependent), NFKBIA, Cathepsin D, Bid
62	Apoptosis and survival_BAD phosphorylation	42	1.17E-07	2.72E-06	17	PI3K cat class IA, p70 S6 kinase1, Calcineurin A (catalytic), G-protein alpha-s, PI3K reg class IA, PP1-cat alpha, Bcl-2, 14-3-3, G-protein beta/gamma, PKA-reg (cAMP-dependent), PP2C, PP2A catalytic, AKT(PKB), Cytochrome c, PDK (PDPK1), PKA-cat (cAMP-dependent), p90Rsk
63	Signal transduction_Activation of PKC via G-Protein coupled receptor	52	1.48E-07	3.38E-06	19	MLCP (cat), MLCP (reg), I-kB, PKC-beta, GSK3 beta, PKC-eta, Calcineurin A (catalytic), Calmodulin, PLC-beta, NF-AT1(NFATC2), PKC-lambda/iota, G-protein beta/gamma, HDAC7, MEF2, NF-kB, c-Src, Calcineurin B (regulatory), MLCK, IP3 receptor
64	Signal transduction_Angiotensin II signaling via Beta-arrestin	57	1.51E-07	3.41E-06	20	MLCP (cat), Casein kinase II, beta chain (Phosvitin), MLCP (reg), ROCK, p70 S6 kinases, GSK3 beta, AP-2 alpha subunits, ASK1 (MAP3K5), MRLC, eIF4E, 14-3-3, PP2A catalytic, AKT(PKB), Beta-arrestin1, MYLK1, c-Src, p27KIP1, p90Rsk, MLCK, MEK4(MAP2K4)
65	Development_PTHR1 in bone and cartilage development	78	1.56E-07	3.46E-06	24	G-protein alpha-12 family, GSK3 beta, PKC-beta1, G-protein alpha-s, PKC, PLC-beta, PP1-cat, Adenylate cyclase, Bcl-2, MKP-1, MEF2C, ATF-4, CREB1, Beta-catenin, MEF2A, PP2A regulatory, PKA-reg (cAMP-dependent), HDAC4, PP2A catalytic, QSK, p300, IGF-1, PKA-cat (cAMP-dependent), G-protein alpha-13
66	GLP-1 in beta cell apoptosis in type 2 diabetes	48	1.99E-07	4.33E-06	18	Thioredoxin, I-kB, c-IAP2, GSK3 beta, FKHR, IKK-alpha, Caspase-3, Bcl-2, CREB1, PKA-reg (cAMP-dependent), p90RSK1, NF-kB, Cytochrome c, Beta-arrestin1, p300, IGF-1, MKK7 (MAP2K7), PKA-cat (cAMP-dependent)
67	Signal transduction_CXCR4 signaling via MAPKs cascades	53	2.1E-07	4.44E-06	19	CD69, ROCK, RelA (p65 NF-kB subunit), NF-kB1 (p50), K-RAS, MEKK1(MAP3K1), CalDAG-GEFII, G-protein alpha-i family, ACKR3, CREB1, Cortactin, G-protein beta/gamma, p90RSK2(RPS6KA3), c-Src, p38 MAPK, NFKBIA, CXCR4, Rac1, G-protein alpha-13
68	EGFR signaling pathway in lung cancer	53	2.1E-07	4.44E-06	19	STAT3, COX-2 (PTGS2), JAK2, PI3K reg class IA (p85), PI3K cat class IA, p70 S6 kinase1, PTEN, HIF1A, IKK-alpha, Bcl-2, STAT5, VEGF-A, NF-kB, AKT(PKB), PDK (PDPK1), c-Src, Mcl-1, NFKBIA, CXCR4

Supplementary Table S3. (Continued)

#	Maps	Total	p-value	FDR	In Data	Network Objects from Active Data
69	IL-6 signaling pathway in lung cancer	35	2.66E-07	5.56E-06	15	STAT3, COX-2 (PTGS2), I-kB, JAK2, PI3K cat class IA, PI3K reg class IA, HIF1A, IKK-alpha, VEGF-A, NF-kB, AKT(PKB), PDK (PDPK1), c-Src, p38 MAPK, IL6RA
70	PDE4 regulation of cyto/chemokine expression in arthritis	49	2.86E-07	5.79E-06	18	NF-kB p50/p50, RAP-1A, PGE2R4, I-kB, PI3K cat class IA, RelA (p65 NF-kB subunit), NF-kB1 (p50), IL-8, IFN-gamma, GSK3 alpha/beta, CREB1, CD28, PKA-reg (cAMP-dependent), AKT(PKB), NF-kB p50/p65, PDE4, PKA-cat (cAMP-dependent), CARD8
71	Signal transduction_Soluble CXCL16 signaling	49	2.86E-07	5.79E-06	18	STAT3, I-kB, JAK2, ROCK, p70 S6 kinase1, alpha-IIb/beta-3 integrin, CD44, HIF1A, IKK-alpha, IL-8, G-protein alpha-i family, G-protein beta/gamma, VEGF-A, NF-kB, AKT(PKB), PDK (PDPK1), P-selectin, p38 MAPK
72	Immune response_IFN-gamma signaling via JAK/STAT and PLC-gamma	54	2.94E-07	5.79E-06	19	STAT3, IRF8, IFNGR1, JAK2, GSK3 beta, Calmodulin, IFN-gamma, CREB1, NOXA, MEF2A, PKA-reg (cAMP-dependent), IBRDC3, p300, c-Src, PKA-cat (cAMP-dependent), MEF2B, Rac1, IP3 receptor, C/EBPdelta
73	NRF2 regulation of oxidative stress response	54	2.94E-07	5.79E-06	19	Casein kinase II, alpha chains, Casein kinase II, beta chain (Phosvitin), Thioredoxin, PI3K cat class IA, GSK3 beta, CRM1, PKC, SOD1, PI3K reg class IA, KEAP1, Cul3/KEAP1/Rbx1 E3 ligase, BACH1, SMRT, DJ-1, AKT(PKB), GCL cat, PDK (PDPK1), PRDX1, MEK4(MAP2K4)
74	Apoptosis and survival_Beta-2 adrenergic receptor anti-apoptotic action	23	2.99E-07	5.79E-06	12	FOXO4, PI3K reg class IA (p85-alpha), G-protein alpha-s, FKHR, G-protein alpha-i family, G-protein beta/gamma, PKA-reg (cAMP-dependent), Beta-2 adrenergic receptor, AKT(PKB), PDK (PDPK1), PKA-cat (cAMP-dependent), Adenylate cyclase type VII
75	EGFR family signaling in pancreatic cancer	75	3.02E-07	5.79E-06	23	STAT3, COX-2 (PTGS2), I-kB, JAK2, PI3K reg class IA (p85), PI3K cat class IA, p70 S6 kinase1, Rb protein, IKK-alpha, Bcl-2, gp91-phox, VEGF-A, NF-kB, AKT(PKB), MKK7 (MAP2K7), PDK (PDPK1), c-Src, p27KIP1, c-Myc, p38 MAPK, PLAU (UPA), Rac1, MEK4(MAP2K4)
76	Cytoskeleton remodeling_Substance P mediated membrane blebbing	16	3.1E-07	5.87E-06	10	MLCP (cat), MLCP (reg), Dynamin-1, G-protein alpha-12 family, Tubulin alpha, MRLC, ROCK1, Dynamin, MLCK, Tubulin (in microtubules)

Supplementary Table S3. (Continued)

#	Maps	Total	p-value	FDR	In Data	Network Objects from Active Data
77	Development_Role of IL-8 in angiogenesis	65	3.7E-07	6.81E-06	21	STAT3, I-kB, JAK2, PI3K reg class IA (p85), PI3K cat class IA, PI3K reg class IA (p85-alpha), IL-8, HMDH, G-protein alpha-i family, SCAP, G-protein beta/gamma, VEGF-A, E2N(UBC13), NF-kB, AKT(PKB), PDK (PDPK1), FASN, c-Src, S1P, Rac1, CARD11
78	Immune response_TNF-R2 signaling pathways	45	3.83E-07	6.81E-06	17	I-kB, TNF-R2, c-IAP2, PI3K reg class IA (p85), PI3K cat class IA, RelA (p65 NF-kB subunit), PI3K reg class IA, IKK-alpha, c-IAP1, ASK1 (MAP3K5), Bcl-2, NF-kB, AKT(PKB), NF-kB p50/p65, PDK (PDPK1), p38 MAPK, MEK4(MAP2K4)
79	IGF family signaling in colorectal cancer	60	3.96E-07	6.81E-06	20	COX-2 (PTGS2), I-kB, GSK3 beta, p70 S6 kinase1, RelA (p65 NF-kB subunit), PI3K reg class IA (p85-alpha), PTEN, HIF1A, IKK-alpha, IL-8, c-Myb, eIF4E, Clusterin, GSK3 alpha/beta, Beta-catenin, VEGF-A, NF-kB, AKT(PKB), IGF-1, MAT2A
80	Immune response_LTBR1 signaling	60	3.96E-07	6.81E-06	20	COX-2 (PTGS2), RAP-1A, I-kB, PI3K cat class IA, AUF1, Calmodulin, CRM1, PKC, PLC-beta, IKK-alpha, G-protein alpha-i family, Adenylate cyclase, eNOS, G-protein beta/gamma, AKT(PKB), NF-kB p50/p65, PDK (PDPK1), Rac1, IP3 receptor, PLC-beta2
81	Signal transduction_S1P4 receptor and S1P5 receptor signaling	50	4.07E-07	6.81E-06	18	COX-2 (PTGS2), ROCK, G-protein alpha-12 family, PI3K cat class IA, RelA (p65 NF-kB subunit), PI3K reg class IA, PLC-beta, PTEN, G-protein alpha-i family, Adenylate cyclase, G-protein beta/gamma, CDC42, NF-kB, AKT(PKB), PDK (PDPK1), p38 MAPK, NFKBIA, IP3 receptor
82	PI3K signaling in gastric cancer	50	4.07E-07	6.81E-06	18	I-kB, PI3K reg class IA (p85), PI3K cat class IA, GSK3 beta, RelA (p65 NF-kB subunit), PI3K reg class IA (p85-alpha), PI3K reg class IA, PTEN, HIF1A, IKK-alpha, IL-8, alpha-V/beta-3 integrin, Beta-catenin, PRNP, AKT(PKB), NF-kB p50/p65, PDK (PDPK1), c-Src
83	IGF-1 signaling in multiple myeloma	50	4.07E-07	6.81E-06	18	I-kB, c-IAP2, PI3K cat class IA, FOXO4, p70 S6 kinase1, PI3K reg class IA, PTEN, FKHR, HIF1A, IKK-alpha, XIAP, GSK3 alpha/beta, VEGF-A, CDC42, NF-kB, AKT(PKB), IGF-1, PDK (PDPK1)
84	The role of PTEN and PI3K signaling in melanoma	50	4.07E-07	6.81E-06	18	STAT3, Epo receptor, JAK2, PI3K reg class IA (p85), PI3K cat class IA, GSK3 beta, p70 S6 kinase1, PTEN, alpha-V/beta-3 integrin, XIAP, AKT3, Caspase-3, Beta-catenin, AKT(PKB), Caspase-8, PDK (PDPK1), Mcl-1, Rac1

Supplementary Table S3. (Continued)

#	Maps	Total	p-value	FDR	In Data	Network Objects from Active Data
85	HBV-dependent NF-kB and PI3K/AKT pathways leading to HCC	50	4.07E-07	6.81E-06	18	I-kB, PI3K reg class IA (p85), PI3K cat class IA, GSK3 beta, RelA (p65 NF-kB subunit), PI3K reg class IA (p85-alpha), NF-kB1 (p105), PTEN, IKK-alpha, NF-kB, AKT(PKB), NF-kB p50/p65, PDK (PDPK1), VBP-1, c-Src, p27KIP1, NFKBIA, PLAU (UPA)
86	PDE4 regulation of cyto/chemokine expression in inflammatory skin diseases	50	4.07E-07	6.81E-06	18	NF-kB p50/p50, RelA (p65 NF-kB subunit), NF-kB1 (p50), NF-AT1(NFATC2), IL23A, PKA-cat alpha, IL-8, G-protein alpha-i family, Adenylate cyclase, IFN-gamma, 14-3-3, CREB1, PKA-reg (cAMP-dependent), NF-kB p50/p65, PDE4, p38 MAPK, PKA-cat (cAMP-dependent), NFKBIA
87	Development_Thrombospondin 1 signaling	36	4.16E-07	6.88E-06	15	MLCP (cat), MLCP (reg), ITGB3, G-protein alpha-i family, Adenylate cyclase, Caspase-3, eNOS, G-protein beta/gamma, VEGF-A, PKA-reg (cAMP-dependent), Caspase-8, Thrombospondin 1, p38 MAPK, PKA-cat (cAMP-dependent), CD47
88	Development_Positive regulation of STK3/4 (Hippo) pathway and negative regulation of YAP/TAZ function	71	4.46E-07	7.29E-06	22	MOBKL1A, WW45, Casein kinase I delta, SCRIB, MPP5, G-protein alpha-s, PKA-cat alpha, STK3, AMPK beta subunit, Adenylate cyclase, FasR(CD95), 14-3-3, Mol1b, AMPK gamma subunit, Beta-catenin, Skp2/TrCP/FBXW, PKA-reg (cAMP-dependent), Beta-2 adrenergic receptor, PP2A cat (alpha), LRR-1, Axin, PKA-cat (cAMP-dependent)
89	Apoptosis and survival_Phosphorylation in TNF-alpha-induced NF-kB signaling	41	5.01E-07	8.11E-06	16	PI3K reg class IA (p85), PI3K cat class IA, RelA (p65 NF-kB subunit), PKA-cat alpha, IKK-alpha, Adenylate cyclase, Casein kinase II, alpha chain (CSNK2A1), PKA-reg (cAMP-dependent), AZI2, TAB2, NF-kB p50/p65, PDK (PDPK1), c-Src, NFKBIA, NF-kB p65/p65, CDK6
90	Signal transduction_Angiotensin II/ AGTR1 signaling via p38, ERK and PI3K	100	5.18E-07	8.28E-06	27	COX-2 (PTGS2), JAK2, p70 S6 kinases, PI3K cat class IA, RECK, PI3K reg class IA (p85-alpha), Calmodulin, ALOX12, FKHR, CalDAG-GEFII, TLR4, eIF4E, MEF2C, SP3, PDGF-A, PDGF-C, CREB1, G-protein beta/gamma, MEF2A, MSK1/2 (RPS6KA5/4), HDAC4, AKT(PKB), PDK (PDPK1), c-Src, p38 MAPK, PKA-cat (cAMP-dependent), p90Rsk

Supplementary Table S3. (Continued)

#	Maps	Total	p-value	FDR	In Data	Network Objects from Active Data
91	Immune response_IL-15 signaling via MAPK and PI3K cascades	56	5.64E-07	8.93E-06	19	IL-15, p70 S6 kinases, PI3K reg class IA (p85), PI3K cat class IA, IL-15RA, RelA (p65 NF-kB subunit), FKHR, IL-8, XBP1, Bcl-2, eIF4E, NOXA, NF-kB, AKT(PKB), NF-kB p50/p65, PDK (PDPK1), Mcl-1, p38 MAPK, Bid
92	DNA damage_ATM/ATR regulation of G2/M checkpoint: cytoplasmic signaling	51	5.72E-07	8.96E-06	18	MLCP (cat), MLCP (reg), TAO2, BORA, IPP-2, MEKK1(MAP3K1), PP1-cat, ATM, 14-3-3, JAB1, 14-3-3 gamma, GADD45 alpha, PP2A regulatory, hnRNP A0, PP2A catalytic, ATR, p38 MAPK, MEK4(MAP2K4)
93	Development_Growth factors in regulation of oligodendrocyte precursor cell survival	37	6.37E-07	9.87E-06	15	PI3K reg class IA (p85), PI3K cat class IA, GSK3 beta, p70 S6 kinase1, RelA (p65 NF-kB subunit), PI3K reg class IA, IKK-alpha, Caspase-3, Bcl-2, PDGF-A, AKT(PKB), IGF-1, PDK (PDPK1), p90Rsk, NFKBIA
94	Immune response_IL-11 signaling pathway via MEK/ERK and PI3K/AKT cascades	67	6.55E-07	1E-05	21	I-kB, JAK2, p70 S6 kinases, PI3K reg class IA (p85), GSK3 beta, Rb protein, RelA (p65 NF-kB subunit), IKK-alpha, IL-8, RPS6, ATF-1, Caspase-3, IFN-gamma, CREB1, AKT(PKB), PDK (PDPK1), SFK, c-Src, p27KIP1, p90Rsk, NFKBIA
95	Signal transduction_Angiotensin II/ AGTR1 signaling via RhoA and JNK	78	6.59E-07	1E-05	23	MLCP (cat), MLCP (reg), JAK2, ROCK, LBC, G-protein alpha-12 family, RECK, Calmodulin, NCK1, Endothelin-1, MEKK1(MAP3K1), Vinculin, TLR4, MRLC, eNOS, G-protein beta/gamma, VEGF-A, MKK7 (MAP2K7), c-Src, MLCK, Rac1, IP3 receptor, MEK4(MAP2K4)
96	Inhibition of apoptosis in gastric cancer	42	7.38E-07	1.1E-05	16	c-IAP2, ROCK, Caspase-10, Caspase-7, c-IAP1, TGF-beta receptor type II, tBid, XIAP, Caspase-3, Bcl-2, NF-kB, AKT(PKB), Cytochrome c, Caspase-8, Mcl-1, Bid
97	Apoptosis and survival_Lymphotoxin-beta receptor signaling	42	7.38E-07	1.1E-05	16	I-kB, LTBR(TNFRSF3), Caspase-7, RelA (p65 NF-kB subunit), NF-kB1 (p50), MEKK1(MAP3K1), IKK-alpha, c-IAP1, IL-8, ASK1 (MAP3K5), Caspase-3, Cytochrome c, NF-kB p50/p65, MKK7 (MAP2K7), TRAF5, MEK4(MAP2K4)
98	CHDI_Correlations from Discovery data_Causal network	73	7.61E-07	1.11E-05	22	PI3K reg class IA (p85), PI3K cat class IA, GSK3 beta, Calmodulin, Sin3A, PLC-beta, PTEN, WNT, G-protein alpha-i family, PDGF-A, CREB1, Beta-catenin, G-protein beta/gamma, MEF2, HDAC5, AKT(PKB), c-Src, c-Myc, YY1, Axin, p90Rsk, IP3 receptor
99	Development_Non-genomic action of Retinoic acid in cell differentiation	57	7.71E-07	1.11E-05	19	COX-2 (PTGS2), PI3K reg class IA (p85), Rb protein, PI3K reg class IA (p85-alpha), PRKAR2B, PKA-cat alpha, HIF1A, CREB1, VEGF-A, PKA-reg type II (cAMP-dependent), p90RSK2(RPS6KA3), p90RSK1, AKT(PKB), PDK (PDPK1), c-Src, PKA-cat (cAMP-dependent), p90Rsk, Rac1, IP3 receptor

Supplementary Table S3. (Continued)

#	Maps	Total	p-value	FDR	In Data	Network Objects from Active Data
100	Development_Cytokine-mediated regulation of megakaryopoiesis	57	7.71E-07	1.11E-05	19	STAT3, JAK2, PI3K reg class IA (p85), PI3K cat class IA, GSK3 beta, p70 S6 kinase1, PI3K reg class IA, alpha-IIb/beta-3 integrin, ITGA2B, PP1-cat, IFN-gamma, c-MPL, STAT5, NF-kB, AKT(PKB), sIL6-RA, p27KIP1, c-Myc, CXCR4
101	Development_PIP3 signaling in cardiac myocytes	47	7.9E-07	1.11E-05	17	G-protein alpha-12 family, PI3K cat class IA, PARD3, p70 S6 kinase1, PI3K reg class IA, PTEN, RPS6, 14-3-3, GSK3 alpha/beta, CREB1, G-protein beta/gamma, CDC42, AKT(PKB), IGF-1, PDK (PDPK1), c-Myc, p90Rsk
102	Canonical Leptin pathways in breast cancer	47	7.9E-07	1.11E-05	17	STAT3, I-kB, JAK2, PI3K cat class IA, GSK3 beta, RelA (p65 NF-kB subunit), PI3K reg class IA, HIF1A, Tcf(Lef), Beta-catenin, VEGF-A, NF-kB, AKT(PKB), NF-kB p50/p65, PDK (PDPK1), c-Myc, Axin
103	Development_MAG, Reticulon 4 and OMgp in inhibition of neurite outgrowth	79	8.46E-07	1.18E-05	23	MLCP (cat), PDK1, RAP-1A, MLCP (reg), SH3RF, ROCK, GSK3 beta, MARK2, Tubulin alpha, Calmodulin, PTEN, alpha-V/beta-3 integrin, MRLC, PP2A catalytic, AKT(PKB), WNK1, ITGA5, Reticulon 4, c-Src, S1P2 receptor, LRP1, G-protein alpha-13, Tubulin (in microtubules)
104	Signal transduction_mTORC2 downstream signaling	68	8.63E-07	1.2E-05	21	Rictor, cPKC (conventional), GSK3 beta, PKC, FKHR, TLR4, Rab-10, GSK3 alpha/beta, PRAS40, Beta-catenin, SGK1, CDC42, PKA-reg (cAMP-dependent), AKT(PKB), FASN, p27KIP1, c-Myc, Mcl-1, PKA-cat (cAMP-dependent), PKC-beta2, Rac1
105	Development_Glucocorticoid receptor signaling	25	9.59E-07	1.31E-05	12	GCR Beta, GCR, SUMO-1, Oct-1, STAT5, GCR Alpha, NCOA1 (SRC1), NF-kB, HSP90, p300, HSP70, NFKBIA
106	Immune response_IL-6 signaling pathway via MEK/ERK and PI3K/AKT cascades	74	9.85E-07	1.34E-05	22	STAT3, JAK2, PI3K reg class IA (p85), PI3K cat class IA, GSK3 beta, Hck, p70 S6 kinase1, PI3K reg class IA, K-RAS, FKHR, RPS6, XIAP, eIF4E, CREB1, p90RSK1, AKT(PKB), sIL6-RA, PDK (PDPK1), p27KIP1, Mcl-1, IP3 receptor, IL6RA
107	Signal transduction_AKT signaling	43	1.07E-06	1.43E-05	16	PCNA, I-kB, PI3K cat class IA, p70 S6 kinase1, PI3K reg class IA, PTEN, IKK-alpha, RPS6, GSK3 alpha/beta, PP2A catalytic, NF-kB, AKT(PKB), HSP90, PDK (PDPK1), p27KIP1, c-Myc
108	Development_S1P3 receptor signaling pathway	43	1.07E-06	1.43E-05	16	PI3K cat class IA, Calmodulin, PI3K reg class IA, PLC-beta, CD44, G-protein alpha-i family, Adenylate cyclase, ROCK1, eNOS, G-protein beta/gamma, AKT(PKB), PDK (PDPK1), c-Src, Rac1, IP3 receptor, G-protein alpha-13

Supplementary Table S4. Enrichment by Pathway Maps of 2005 downregulated genes (Pattern 1).

#	Maps	Total	p-value	FDR	In Data	Network Objects from Active Data
1	Signal transduction_mTORC1 downstream signaling	60	3.3E-10	4.5E-07	19	PDK1, Rictor, p70 S6 kinases, p70 S6 kinase1, LIPIN1, HIF1A, MTHFD2, PDCD4, RPS6, eIF4A, ACSL3, eIF4E, ATF-4, SGK1, Cytochrome c, CLIP170, ULK2, p27KIP1, YY1
2	Apoptosis and survival_Role of PKR in stress-induced apoptosis	53	2.3E-09	1.6E-06	17	PACT, I-kB, Caspase-7, IKK-alpha, TLR4, IFN-gamma, Caspase-3, FasR(CD95), eIF4E, PPP2R5A, ATF-4, PP2A regulatory, PP2A catalytic, eIF2S1, TAB2, c-Myc, NFKBIA
3	Apoptosis and survival_TNFR1 signaling pathway	43	5.4E-09	2E-06	15	I-kB, c-IAP2, Caspase-7, MEKK1(MAP3K1), c-IAP1, tBid, XIAP, RAIDD, Caspase-3, Bcl-2, jBid, Cytochrome c, ERAP1, MEK4(MAP2K4), Bid
4	Apoptosis and survival_Endoplasmic reticulum stress response pathway	56	5.8E-09	2E-06	17	I-kB, Caspase-7, XBP1, PP1-cat, ASK1 (MAP3K5), tBid, Bcl-2, ATF-4, IP3R1, eIF2S1, Cytochrome c, Calpain 2(m), S1P, Derlin-2, HERP, MEK4(MAP2K4), Bid
5	Signal transduction_Calcium-mediated signaling	72	9.9E-09	2.4E-06	19	MLCP (cat), COX-2 (PTGS2), MLCP (reg), I-kB, PKC-beta, ROCK, cPKC (conventional), Calcineurin A (catalytic), Calmodulin, PKC, ASK1 (MAP3K5), 14-3-3, CREB1, HDAC4, MEF2, AKT(PKB), Rac1, IP3 receptor, MEK4(MAP2K4)
6	Chemotaxis_Lysophosphatidic acid signaling via GPCRs	129	1E-08	2.4E-06	26	MLCP (reg), ROCK, PI3K reg class IA (p85), cPKC (conventional), G-protein alpha-12 family, Caspase-7, p70 S6 kinase1, PKC, FKHR, Tcf(Lef), IL-8, LPAR6, Caspase-3, ROCK1, Bcl-2, FasR(CD95), MKL2, CREB1, Beta-catenin, G-protein beta/gamma, Rho GTPase, CDC42, AKT(PKB), Rac1, IP3 receptor, MEK4(MAP2K4)
7	Cell cycle_Influence of Ras and Rho proteins on G1/S Transition	53	1.8E-08	3.4E-06	16	MLCP (cat), MLCP (reg), p70 S6 kinase1, Rb protein, PI3K reg class IA, IKK-alpha, MRLC, CDC42, AKT(PKB), RalA, p27KIP1, c-Myc, NFKBIA, Rac1, MEK4(MAP2K4), CDK6
8	Proteolysis_Putative SUMO-1 pathway	29	2E-08	3.4E-06	12	GCR, SUMO-1, c-Myb, FasR(CD95), SP3, SENP1, HSF2, SAE2, TOP2, SP100, RanBP2, NFKBIA
9	CHDI_Correlations from Replication data_Causal network (positive correlations)	79	5.1E-08	7.6E-06	19	I-kB, ROCK, PI3K reg class IA (p85), Calcineurin A (catalytic), Calmodulin, CD44, IKK-alpha, Caspase-3, CREB1, CD28, IP3R1, G-protein beta/gamma, IRAK1/2, MEF2, AKT(PKB), HSP70, CXCR4, IP3 receptor, MEK4(MAP2K4)
10	Development_Thromboxane A2 signaling pathway	50	5.5E-08	7.6E-06	15	PI3K reg class IA (p85), cPKC (conventional), G-protein alpha-12 family, p70 S6 kinase1, PKC, PI3K reg class IA, Tcf(Lef), RAP-1B, Adenylate cyclase, CREB1, Beta-catenin, AKT(PKB), PKA-cat (cAMP-dependent), IP3 receptor, G-protein alpha-13

Supplementary Table S4. (Continued)

#	Maps	Total	p-value	FDR	In Data	Network Objects from Active Data
11	DNA damage_ATM/ATR regulation of G2/M checkpoint: cytoplasmic signaling	51	7.4E-08	9.3E-06	15	MLCP (cat), MLCP (reg), BORA, IPP-2, MEKK1(MAP3K1), PP1-cat, ATM, 14-3-3, JAB1, 14-3-3 gamma, PP2A regulatory, hnRNP A0, PP2A catalytic, ATR, MEK4(MAP2K4)
12	Development_The role of GDNF ligand family/ RET receptor in cell survival, growth and proliferation	92	1.4E-07	1.6E-05	20	RAP-1A, ROCK, PI3K reg class IA (p85), Calmodulin, CREM (activators), NCK1, MEKK1(MAP3K1), HIF1A, IKK-alpha, XIAP, ATF-1, Bcl-2, CREB1, CDC42, p90RSK2(RPS6KA3), AKT(PKB), NFKBIA, Rac1, IP3 receptor, MEK4(MAP2K4)
13	Transport_RAN regulation pathway	18	1.7E-07	1.7E-05	9	CHC1L, CRM1, SUMO-1, NUP58, NUP153, NUP54, Importin (karyopherin)-alpha, RanBP2, Ran
14	Immune response_TLR2 and TLR4 signaling pathways	69	1.8E-07	1.7E-05	17	COX-2 (PTGS2), I-kB, PI3K reg class IA (p85), Pellino 1, IRAK4, IKK-alpha, IL-8, TLR4, MD-2, IRAK2, CREB1, E2N(UBC13), TAB2, AKT(PKB), p90Rsk, Rac1, MEK4(MAP2K4)
15	Apoptosis and survival_NGF/ TrkA PI3K-mediated signaling	77	1.8E-07	1.7E-05	18	MLCP (cat), RAP-1A, MLCP (reg), ROCK, PI3K reg class IA (p85), p70 S6 kinase1, Calcineurin A (catalytic), Calmodulin, FKHR, N-WASP, Destrin, MRLC, Bcl-2, CREB1, CDC42, VAV-3, AKT(PKB), Rac1
16	Inhibition of apoptosis in gastric cancer	42	2.8E-07	2.4E-05	13	c-IAP2, ROCK, Caspase-7, c-IAP1, TGF-beta receptor type II, tBid, XIAP, Caspase-3, Bcl-2, AKT(PKB), Cytochrome c, Mcl-1, Bid
17	Glucocorticoids-mediated inhibition of pro-constrictory and pro-inflammatory signaling in airway smooth muscle cells	49	3E-07	2.4E-05	14	MLCP (cat), COX-2 (PTGS2), MLCP (reg), GCR Beta, PDE4D, GCR, MRLC, IFN-gamma, MKP-1, GCR Alpha, Beta-2 adrenergic receptor, PLA2, NFKBIA, Histone H4
18	Apoptosis and survival_HTR1A signaling	50	4E-07	3E-05	14	I-kB, JAK2, Calmodulin, IKK-alpha, Adenylate cyclase, XIAP, Caspase-3, Bcl-2, G-protein beta/gamma, PP2A regulatory, PP2A catalytic, AKT(PKB), Cytochrome c, PKA-cat (cAMP-dependent)
19	Oxidative stress_ROS-induced cellular signaling	108	5E-07	3.5E-05	21	TfR1, COX-2 (PTGS2), PKC-beta, p70 S6 kinase1, PKC, PTEN, MEKK1(MAP3K1), HIF1A, IKK-alpha, IL-8, ATM, DLC1 (Dynein LC8a), IRP2, GRP75, SENP1, NOXA, SAE2, AKT(PKB), Glutaredoxin 1, Cytochrome c, NFKBIA
20	Signal transduction_Non-canonical WNT5A signaling	82	5E-07	3.5E-05	18	MLCP (cat), MLCP (reg), ROCK, cPKC (conventional), Calcineurin A (catalytic), Calmodulin, CCDC88C, PKC-lambda/iota, IL-8, Lef-1, Beta-catenin, G-protein beta/gamma, CDC42, TAB2, RYK, NF-AT, Rac1, IP3 receptor

Supplementary Table S4. (Continued)

#	Maps	Total	p-value	FDR	In Data	Network Objects from Active Data
21	Signal transduction_Angiotensin II/ AGTR1 signaling via p38, ERK and PI3K	100	5.9E-07	3.8E-05	20	COX-2 (PTGS2), JAK2, p70 S6 kinases, RECK, PI3K reg class IA (p85-alpha), Calmodulin, FKHR, CalDAG-GEFII, TLR4, eIF4E, MEF2C, SP3, PDGF-C, CREB1, G-protein beta/gamma, MEF2A, HDAC4, AKT(PKB), PKA-cat (cAMP-dependent), p90Rsk
22	Immune response_IL-11 signaling pathway via MEK/ERK and PI3K/AKT cascades	67	6.5E-07	4.1E-05	16	I-kB, JAK2, p70 S6 kinases, PI3K reg class IA (p85), Rb protein, IKK-alpha, IL-8, RPS6, ATF-1, IFN-gamma, Caspase-3, CREB1, AKT(PKB), p27KIP1, p90Rsk, NFKBIA
23	Immune response_ICOS signaling pathway in T-helper cell	60	7.7E-07	4.2E-05	15	ROCK, PI3K reg class IA (p85), p70 S6 kinase1, Calcineurin A (catalytic), PI3K reg class IA (p85-alpha), Calmodulin, PI3K reg class IA, FKHR, IFN-gamma, CD28, CDC42, AKT(PKB), ICOS, Rac1, IP3 receptor
24	Development_Positive regulation of WNT/Beta-catenin signaling in the cytoplasm	76	7.9E-07	4.2E-05	17	COX-2 (PTGS2), GSKIP, BIG1, BIG2, USP25, Tcf(Lef), UBE2B, PP1-cat, 14-3-3, Beta-catenin, HSP105, TBLR1, PP2A catalytic, AKT(PKB), PKA-cat (cAMP-dependent), Rac1, DOCK4
25	Development_VEGF signaling via VEGFR2 - generic cascades	93	7.9E-07	4.2E-05	19	COX-2 (PTGS2), I-kB, PKC-beta, Calcineurin A (catalytic), Calmodulin, PKC, NCK1, PI3K reg class IA, MEKK1(MAP3K1), ROCK1, eIF4E, CREB1, Beta-catenin, CDC42, AKT(PKB), HSP90, p90Rsk, Rac1, IP3 receptor
26	Signal transduction_mTORC2 downstream signaling	68	8E-07	4.2E-05	16	Rictor, cPKC (conventional), PKC, FKHR, TLR4, Rab-10, Beta-catenin, SGK1, CDC42, AKT(PKB), p27KIP1, c-Myc, Mcl-1, PKA-cat (cAMP-dependent), PKC-beta2, Rac1
27	Immune response_IL-3 signaling via ERK and PI3K	102	8.2E-07	4.2E-05	20	RAP-1A, JAK2, p70 S6 kinases, PI3K reg class IA (p85), cPKC (conventional), Calcineurin A (catalytic), Calmodulin, RPS6, Bcl-2, CREB1, CDC42, AKT(PKB), PDE4, p27KIP1, Mcl-1, PKA-cat (cAMP-dependent), p90Rsk, LPCAT2, Rac1, IP3 receptor
28	IL-2 as a growth factor for T cells in multiple sclerosis	33	1E-06	5.1E-05	11	PCNA, PI3K reg class IA (p85), p70 S6 kinase1, Rb protein, FKHR, Bcl-2, c-Myb, CREB1, AKT(PKB), p27KIP1, c-Myc
29	Development_PTHR1 in bone and cartilage development	78	1.2E-06	5.4E-05	17	G-protein alpha-12 family, PKC-beta1, PKC, PP1-cat, Adenylate cyclase, Bcl-2, MKP-1, MEF2C, ATF-4, CREB1, Beta-catenin, MEF2A, PP2A regulatory, HDAC4, PP2A catalytic, PKA-cat (cAMP-dependent), G-protein alpha-13
30	Apoptosis and survival_Ceramides signaling pathway	40	1.2E-06	5.4E-05	12	PI3K reg class IA (p85), PI3K reg class IA (p85-alpha), MEKK1(MAP3K1), tBid, Caspase-3, Bcl-2, FasR(CD95), PP2A catalytic, AKT(PKB), Cytochrome c, MEK4(MAP2K4), Bid

Supplementary Table S4. (Continued)

#	Maps	Total	p-value	FDR	In Data	Network Objects from Active Data
31	Signal transduction_PDGF signaling via PI3K/AKT and NFkB pathways	70	1.2E-06	5.4E-05	16	PDK1, PI3K reg class IA (p85), Calmodulin, PTEN, FKHR, HXK2, HIF1A, PDGF-C, Beta-catenin, SGK1, AKT(PKB), DDX5, p27KIP1, c-Myc, NFKBIA, Rac1
32	Apoptosis and survival_BAD phosphorylation	42	2.1E-06	9.2E-05	12	p70 S6 kinase1, Calcineurin A (catalytic), PI3K reg class IA, Bcl-2, 14-3-3, G-protein beta/gamma, PP2C, PP2A catalytic, AKT(PKB), Cytochrome c, PKA-cat (cAMP-dependent), p90Rsk
33	Signal transduction_AKT signaling	43	2.8E-06	0.00011	12	PCNA, I-kB, p70 S6 kinase1, PI3K reg class IA, PTEN, IKK-alpha, RPS6, PP2A catalytic, AKT(PKB), HSP90, p27KIP1, c-Myc
34	Immune response_B cell antigen receptor (BCR) pathway	110	2.8E-06	0.00011	20	PKC-beta, PI3K reg class IA (p85), p70 S6 kinase1, Rb protein, Calcineurin A (catalytic), Calmodulin, NCK1, MEKK1(MAP3K1), FKHR, IKK-alpha, CalDAG-GEFII, CDC42, PP2A catalytic, AKT(PKB), DAPP1, PKC-beta2, NFKBIA, Rac1, IP3 receptor, CDK6
35	Role of CNTF and LIF in regulation of oligodendrocyte development in multiple sclerosis	30	3.2E-06	0.00012	10	JAK2, c-IAP2, IMPA1, PI3K reg class IA, IKK-alpha, IFN-gamma, Caspase-3, 14-3-3, AKT(PKB), NFKBIA
36	DNA damage_Brca1 as a transcription regulator	30	3.2E-06	0.00012	10	PCNA, Rb protein, MSH2, ATM, ATF-1, SP3, ATR, RBBP8 (CtIP), p27KIP1, c-Myc
37	Signal transduction_PKA signaling	51	3.3E-06	0.00012	13	G-protein alpha-12 family, AKAP11, PDE4D, PDE3B, Adenylate cyclase, CREB1, PP2A regulatory, AKAP7 gamma, PKI, PKA-cat (cAMP-dependent), AKAP8, NFKBIA, G-protein alpha-13
38	Leptin signaling in colorectal cancer	44	3.7E-06	0.00013	12	JAK2, p70 S6 kinase1, PI3K reg class IA, PTEN, IKK-alpha, IL-8, Beta-catenin, CDC42, AKT(PKB), c-Myc, NFKBIA, Rac1
39	G-protein signaling_G-Protein alpha-12 signaling pathway	38	4.9E-06	0.00017	11	RAP-1A, ROCK, PI3K reg class IA (p85), G-protein alpha-12 family, MEKK1(MAP3K1), TC21, G-protein beta/gamma, CDC42, PKA-cat (cAMP-dependent), Rac1, MEK4(MAP2K4)
40	Immune response_HMGB1/RAGE signaling pathway	53	5.3E-06	0.00018	13	I-kB, PI3K reg class IA (p85), PI3K reg class IA (p85-alpha), IL-8, TLR4, MEF2C, CREB1, MEF2A, CDC42, p90RSK2(RPS6KA3), AKT(PKB), NFKBIA, Rac1
41	Immune response_IL-16 signaling pathway	54	6.6E-06	0.00022	13	MLCP (cat), IL-15, MLCP (reg), cPKC (conventional), PI3K reg class IA (p85-alpha), PKC, FKHR, Caspase-3, Skp2/TrCP/FBXW, AKT(PKB), p27KIP1, CXCR4, IP3 receptor
42	Immune response_HSP60 and HSP70/ TLR signaling pathway	54	6.6E-06	0.00022	13	CD69, I-kB, IRAK4, IKK-alpha, IL-8, TLR4, MD-2, IRAK1/2, E2N(UBC13), TAB2, HSP60, HSP70, MEK4(MAP2K4)

Supplementary Table S4. (Continued)

#	Maps	Total	p-value	FDR	In Data	Network Objects from Active Data
43	Signal transduction_Adenosine A2B receptor signaling pathway	71	7.4E-06	0.00024	15	PDK1, RAP-1A, PI3K reg class IA (p85), Calcineurin A (catalytic), PKC, HIF1A, RAP-1B, Adenylate cyclase, PER2, CREB1, JAB1, G-protein beta/gamma, AKT(PKB), PKA-cat (cAMP-dependent), IP3 receptor
44	Development_PIP3 signaling in cardiac myocytes	47	7.8E-06	0.00024	12	G-protein alpha-12 family, p70 S6 kinase1, PI3K reg class IA, PTEN, RPS6, 14-3-3, CREB1, G-protein beta/gamma, CDC42, AKT(PKB), c-Myc, p90Rsk
45	Resistance of pancreatic cancer cells to death receptor signaling	33	8.5E-06	0.00025	10	c-IAP2, Caspase-7, c-IAP1, tBid, XIAP, Caspase-3, FasR(CD95), Cytochrome c, NFKBIA, Bid
46	Development_SLIT-ROBO1 signaling	40	8.5E-06	0.00025	11	Rictor, ROCK, PI3K reg class IA (p85), Calcineurin A (catalytic), Calmodulin, NCK1, CDC42, AKT(PKB), Cytohesin1, CXCR4, Rac1
47	Stem cells_Pancreatic cancer stem cells in tumor metastasis	40	8.5E-06	0.00025	11	MLCP (cat), MLCP (reg), ROCK, Calmodulin, MRLC, G-protein beta/gamma, CDC42, CXCR4, Rac1, IP3 receptor, G-protein alpha-13
48	MAPK-independent proliferation of normal and asthmatic smooth muscle cells	64	9.7E-06	0.00028	14	I-kB, JAK2, PI3K reg class IA (p85), p70 S6 kinase1, Rb protein, PI3K reg class IA (p85-alpha), PI3K reg class IA, IKK-alpha, PDGF-C, G-protein beta/gamma, AKT(PKB), p27KIP1, c-Myc, Rac1
49	Regulation and signaling of HGF receptor (Met) and MSP receptor (RON) in lung cancer	73	1.1E-05	0.0003	15	COX-2 (PTGS2), PI3K reg class IA (p85), p70 S6 kinase1, Rb protein, NCK1, MEKK1(MAP3K1), HIF1A, CREB1, Beta-catenin, CDC42, AKT(PKB), PKC-beta2, p90Rsk, Rac1, Gamma adducin
50	Signal transduction_Erk Interactions: Inhibition of Erk	34	1.1E-05	0.0003	10	MKP-3, Calcineurin A (catalytic), Calmodulin, PKC, MKP-1, PP2A catalytic, PTPR-epsilon, AKT(PKB), GMF, PKA-cat (cAMP-dependent)
51	Role of Apo-2L(TNFSF10) in Prostate Cancer cell apoptosis	34	1.1E-05	0.0003	10	I-kB, c-IAP2, Caspase-7, c-IAP1, tBid, XIAP, Caspase-3, Bcl-2, Cytochrome c, Bid
52	Apoptosis and survival_Cytoplasmic/mitochondrial transport of proapoptotic proteins Bid, Bmf and Bim	34	1.1E-05	0.0003	10	MEKK1(MAP3K1), ASK1 (MAP3K5), tBid, DLC1 (Dynein LC8a), Bcl-2, FasR(CD95), GCKR(MAP4K5), Cytochrome c, MEK4(MAP2K4), Bid
53	Signal transduction_Angiotensin II signaling via Beta-arrestin	57	1.2E-05	0.00032	13	MLCP (cat), MLCP (reg), ROCK, p70 S6 kinases, ASK1 (MAP3K5), MRLC, eIF4E, 14-3-3, PP2A catalytic, AKT(PKB), p27KIP1, p90Rsk, MEK4(MAP2K4)
54	Development_Role of CNTF and LIF in regulation of oligodendrocyte development	28	1.4E-05	0.00035	9	JAK2, c-IAP2, IMPA1, PI3K reg class IA, IKK-alpha, Caspase-3, 14-3-3, AKT(PKB), NFKBIA

Supplementary Table S4. (Continued)

#	Maps	Total	p-value	FDR	In Data	Network Objects from Active Data
55	Inhibition of GTPase prenylation by statins in asthma and COPD	42	1.4E-05	0.00035	11	COX-2 (PTGS2), I-kB, ROCK, PTEN, IKK-alpha, IL-8, HMDH, CDC42, AKT(PKB), p27KIP1, Rac1
56	Apoptosis and survival_Lymphotoxin-beta receptor signaling	42	1.4E-05	0.00035	11	I-kB, Caspase-7, MEKK1(MAP3K1), IKK-alpha, c-IAP1, IL-8, ASK1 (MAP3K5), Caspase-3, Cytochrome c, TRAF5, MEK4(MAP2K4)
57	Altered Ca2+ handling in heart failure	35	1.5E-05	0.00037	10	Calcineurin A (catalytic), Calmodulin, PDE4D, PP1-cat, Adenylate cyclase, Ca-ATPase2, G-protein beta/gamma, Beta-2 adrenergic receptor, PP2A catalytic, PKA-cat (cAMP-dependent)
58	Development_GM-CSF signaling	50	1.5E-05	0.00037	12	I-kB, PKC-beta, JAK2, PI3K reg class IA (p85), PI3K reg class IA (p85-alpha), IKK-alpha, Caspase-3, Bcl-2, CREB1, AKT(PKB), c-Myc, Mcl-1
59	Apoptosis and survival_TNF-alpha-induced Caspase-8 signaling	43	1.8E-05	0.00043	11	Caspase-7, tBid, Caspase-3, CYLD, HSP90 alpha, PP2A regulatory, PP2A catalytic, AKT(PKB), HSP90, Cytochrome c, Bid
60	Statin action on the PI3K/ Akt pathway in COPD	36	2E-05	0.00046	10	ROCK, p70 S6 kinases, p70 S6 kinase1, PI3K reg class IA, FKHR, HMDH, AKT(PKB), HSP90, p27KIP1, NIP3
61	IGF family signaling in colorectal cancer	60	2.2E-05	0.00048	13	COX-2 (PTGS2), I-kB, p70 S6 kinase1, PI3K reg class IA (p85-alpha), PTEN, HIF1A, IKK-alpha, IL-8, c-Myb, eIF4E, Beta-catenin, AKT(PKB), MAT2A
62	DNA damage_p53 activation by DNA damage	60	2.2E-05	0.00048	13	MEKK1(MAP3K1), PCAF, ATM, Bcl-2, 14-3-3, NOXA, PP2A regulatory, PP2A catalytic, ATR, P53DINP1a, 14-3-3 theta, DYRK2, MEK4(MAP2K4)
63	Immune response_LTBR1 signaling	60	2.2E-05	0.00048	13	COX-2 (PTGS2), RAP-1A, I-kB, AUF1, Calmodulin, CRM1, PKC, IKK-alpha, Adenylate cyclase, G-protein beta/gamma, AKT(PKB), Rac1, IP3 receptor
64	Neutrophil resistance to apoptosis in COPD and proresolving impact of lipid mediators	60	2.2E-05	0.00048	13	PI3K reg class IA, c-IAP1, FasR(CD95) soluble, tBid, XIAP, Caspase-3, gp91-phox, FasR(CD95), 14-3-3, AKT(PKB), Cytochrome c, Mcl-1, Bid
65	K-RAS signaling in pancreatic cancer	44	2.3E-05	0.00049	11	PTEN, MEKK1(MAP3K1), IKK-alpha, IL-8, Bcl-2, AKT(PKB), ATR, RalA, c-Myc, NFKBIA, Rac1
66	Apoptosis and survival_Regulation of apoptosis by mitochondrial proteins	106	2.3E-05	0.00049	18	OMA1, Calcineurin A (catalytic), GC1QBP, tBid, ROCK1, Bcl-2, Mitofusin 1, NOXA, PP2C, PP2A catalytic, PARL, Cytochrome c, DNM1L (DRP1), Mcl-1, MFF, APG12, NIP3, Bid

Supplementary Table S4. (Continued)

#	Maps	Total	p-value	FDR	In Data	Network Objects from Active Data
67	Role of microRNAs in cell proliferation in colorectal cancer	69	2.4E-05	0.0005	14	PGE2R4, p70 S6 kinase1, Rb protein, PTEN, FBXW7, PI3K class II (CII-alpha), Beta-catenin, CDC42, Rab-22A, FLI1, SNX1, c-Myc, E2F3, CDK6
68	Signal transduction_Angiotensin II/ AGTR1 signaling via RhoA and JNK	78	2.4E-05	0.0005	15	MLCP (cat), MLCP (reg), JAK2, ROCK, G-protein alpha-12 family, RECK, Calmodulin, NCK1, MEKK1(MAP3K1), TLR4, MRLC, G-protein beta/gamma, Rac1, IP3 receptor, MEK4(MAP2K4)
69	Role of IFN-beta in activation of T cell apoptosis in multiple sclerosis	30	2.6E-05	0.00053	9	c-IAP2, Caspase-7, c-IAP1, XIAP, Caspase-3, FasR(CD95), PP2A catalytic, AKT(PKB), PP2A cat (alpha)
70	Effect of H. pylori infection on apoptosis in gastric epithelial cells	53	2.9E-05	0.00055	12	COX-2 (PTGS2), I-kB, c-IAP2, c-IAP1, tBid, Caspase-3, Bcl-2, FasR(CD95), NOXA, Cytochrome c, Mcl-1, Bid
71	Signal transduction_CXCR4 signaling via MAPKs cascades	53	2.9E-05	0.00055	12	CD69, ROCK, MEKK1(MAP3K1), CalDAG-GEFII, ACKR3, CREB1, G-protein beta/gamma, p90RSK2(RPS6KA3), NFKBIA, CXCR4, Rac1, G-protein alpha-13
72	EGFR signaling pathway in lung cancer	53	2.9E-05	0.00055	12	COX-2 (PTGS2), JAK2, PI3K reg class IA (p85), p70 S6 kinase1, PTEN, HIF1A, IKK-alpha, Bcl-2, AKT(PKB), Mcl-1, NFKBIA, CXCR4
73	Proliferative action of Gastrin in gastric cancer	53	2.9E-05	0.00055	12	PKC-beta, JAK2, PI3K reg class IA (p85), cPKC (conventional), p70 S6 kinase1, PKC, SP3, CREB1, Beta-catenin, c-Myc, p90Rsk, IP3 receptor
74	Development_Negative regulation of WNT/Beta-catenin signaling in the nucleus	89	3.2E-05	0.00059	16	NF-AT5, Calcineurin A (catalytic), NARF, BACH1, Tcf(Lef), Lef-1, 14-3-3, Jade-1, SENP1, Beta-catenin, P15RS, TRIM33, TBLR1, TAB2, PJA2, Kaiso
75	DNA damage_ATM activation by DNA damage	71	3.4E-05	0.00062	14	RCAD, HMG14, Pellino 1, SOSSC, ATM, PP2A regulatory, Rad50, p90RSK2(RPS6KA3), eIF3S6, E2N(UBC13), PP2A catalytic, HP1 beta, HSP90, Histone H4
76	Development_Positive regulation of STK3/4 (Hippo) pathway and negative regulation of YAP/TAZ function	71	3.4E-05	0.00062	14	MOBKL1A, WW45, MPP5, STK3, AMPK beta subunit, Adenylate cyclase, FasR(CD95), 14-3-3, Beta-catenin, Skp2/TrCP/FBXW, Beta-2 adrenergic receptor, PP2A cat (alpha), LRR-1, PKA-cat (cAMP-dependent)
77	Translation_Regulation of EIF4F activity	54	3.5E-05	0.00063	12	p70 S6 kinase1, PI3K reg class IA, MEKK1(MAP3K1), eIF4G2, TGF-beta receptor type II, eIF4A, eIF4E, CDC42, PP2A catalytic, AKT(PKB), Rac1, MEK4(MAP2K4)

Supplementary Table S4. (Continued)

#	Maps	Total	p-value	FDR	In Data	Network Objects from Active Data
78	Signal transduction_CXCR4 signaling via PI3K cascade	46	3.6E-05	0.00063	11	PI3K reg class IA (p85), p70 S6 kinase1, FKHR, Caspase-3, Beta-catenin, G-protein beta/gamma, SGK1, AKT(PKB), NFKBIA, CXCR4, Rac1
79	Role of GIP in pathogenesis of type 2 diabetes	46	3.6E-05	0.00063	11	RAP-1A, FKHR, ASK1 (MAP3K5), Caspase-3, Bcl-2, CREB1, PP2A catalytic, AKT(PKB), Cytochrome c, PKA-cat (cAMP-dependent), MEK4(MAP2K4)
80	T follicular helper cell dysfunction in SLE	90	3.7E-05	0.00063	16	RC3H2, IFNGR1, I-kB, PI3K reg class IA (p85), p70 S6 kinase1, Roquin, PI3K reg class IA (p85-alpha), FKHR, IKK-alpha, IFN-gamma, CD28, SAP, SLAM, AKT(PKB), ICOS, BLIMP1 (PRDI-BF1)
81	Development_PACAP signaling in neural cells	39	4.3E-05	0.00069	10	RAP-1A, cPKC (conventional), ATF-1, Caspase-3, Bcl-2, CREB1, p90RSK2(RPS6KA3), Cytochrome c, PKA-cat (cAMP-dependent), IP3 receptor
82	Immune response_HMGB1 release from the cell	39	4.3E-05	0.00069	10	IFNGR1, PKC-beta, PI3K reg class IA (p85), Calmodulin, CRM1, PI3K reg class IA, PCAF, TLR4, IFN-gamma, IP3 receptor
83	Deregulation of Ca2+-dependent neuronal cell survival in Huntington's disease	39	4.3E-05	0.00069	10	PI3K reg class IA (p85), Calcineurin A (catalytic), Calmodulin, PP1-cat, Lef-1, CREB1, IP3R1, Beta-catenin, Calcipressin 1, AKT(PKB)
84	Immune response_Platelet activating factor/ PTAFR pathway signaling	55	4.3E-05	0.00069	12	JAK2, Calcineurin A (catalytic), PI3K reg class IA, IKK-alpha, ASK1 (MAP3K5), Adenylate cyclase, G-protein beta/gamma, AKT(PKB), PKA-cat (cAMP-dependent), NFKBIA, NF-AT, IP3 receptor
85	PGE2 pathways in cancer	55	4.3E-05	0.00069	12	COX-2 (PTGS2), PGE2R4, HIF1A, Tcf(Lef), Lef-1, Adenylate cyclase, CREB1, Beta-catenin, G-protein beta/gamma, AKT(PKB), c-Myc, PKA-cat (cAMP-dependent)
86	Immune response_CD28 signaling	55	4.3E-05	0.00069	12	I-kB, PI3K reg class IA (p85), Calcineurin A (catalytic), Calmodulin, MEKK1(MAP3K1), IKK-alpha, CD28, AKT(PKB), NF-AT, Rac1, IP3 receptor, MEK4(MAP2K4)
87	Immune response_IL-15 signaling via MAPK and PI3K cascades	56	5.2E-05	0.0008	12	IL-15, p70 S6 kinases, PI3K reg class IA (p85), FKHR, IL-8, XBP1, Bcl-2, eIF4E, NOXA, AKT(PKB), Mcl-1, Bid
88	Regulation of Beta-catenin activity in colorectal cancer	56	5.2E-05	0.0008	12	PGE2R4, PKC-beta, PI3K reg class IA (p85), Calmodulin, PTEN, IKK-alpha, PDCD4, Adenylate cyclase, Beta-catenin, AKT(PKB), PKA-cat (cAMP-dependent), IP3 receptor

Supplementary Table S4. (Continued)

#	Maps	Total	p-value	FDR	In Data	Network Objects from Active Data
89	Apoptotic pathways and resistance to apoptosis in lung cancer cells	56	5.2E-05	0.0008	12	c-IAP2, c-IAP1, tBid, XIAP, Caspase-3, Bcl-2, FasR(CD95), CYLD, Cytochrome c, p27KIP1, Mcl-1, Bid
90	Mitogenic action of ErbB2 in breast cancer	56	5.2E-05	0.0008	12	I-kB, PI3K reg class IA (p85), p70 S6 kinase1, FKHR, IKK-alpha, JAB1, Beta-catenin, AKT(PKB), Cullin 3, p27KIP1, c-Myc, Rac1
91	Signal transduction_HTR2A signaling outside the nervous system	74	5.5E-05	0.00081	14	MLCP (cat), COX-2 (PTGS2), MLCP (reg), PKC-beta, JAK2, ROCK, PI3K reg class IA (p85), cPKC (conventional), p70 S6 kinase1, Rb protein, Calmodulin, PKC, Adenylate cyclase, AKT(PKB)
92	TNF-alpha and IL-1 beta-mediated regulation of contraction and secretion of inflammatory factors in normal and asthmatic airway smooth muscle	65	5.5E-05	0.00081	13	COX-2 (PTGS2), PI3K reg class IA (p85), Calmodulin, PCAF, IL-8, gp91-phox, Ca-ATPase2, HDAC4, AKT(PKB), PLA2, PKC-beta2, NFKBIA, Histone H4
93	Ovarian cancer (main signaling cascades)	65	5.5E-05	0.00081	13	I-kB, NCOA4 (ARA70), PI3K reg class IA, PTEN, MEKK1(MAP3K1), IKK-alpha, Tcf(Lef), CREB1, Beta-catenin, G-protein beta/gamma, AKT(PKB), c-Myc, PKA-cat (cAMP-dependent)
94	Signal transduction_Adenosine A3 receptor signaling pathway	48	5.5E-05	0.00081	11	PI3K reg class IA (p85), PKC, HIF1A, Adenylate cyclase, Bcl-2, CREB1, G-protein beta/gamma, Rho GTPase, AKT(PKB), NFKBIA, IP3 receptor
95	SDF-1 axis in endothelial progenitor cell recruitment in healing myocardial infarction	33	6.1E-05	0.00088	9	p70 S6 kinase1, FKHR, HIF1A, RPS6, ACKR3, G-protein beta/gamma, AKT(PKB), CXCR4, Rac1
96	Development_Non-genomic action of Retinoic acid in cell differentiation	57	6.3E-05	0.00088	12	COX-2 (PTGS2), PI3K reg class IA (p85), Rb protein, PI3K reg class IA (p85-alpha), HIF1A, CREB1, p90RSK2(RPS6KA3), AKT(PKB), PKA-cat (cAMP-dependent), p90Rsk, Rac1, IP3 receptor
97	Immune response_Role of PKR in stress-induced antiviral cell response	57	6.3E-05	0.00088	12	PACT, I-kB, Caspase-7, IKK-alpha, IL-8, TLR4, IFN-gamma, Caspase-3, TAB2, c-Myc, NFKBIA, MEK4(MAP2K4)

Supplementary Table S4. (Continued)

#	Maps	Total	p-value	FDR	In Data	Network Objects from Active Data
98	Immune response_IFN-alpha/beta signaling via PI3K and NF-kB pathways	94	6.4E-05	0.00088	16	PCNA, I-kB, p70 S6 kinases, PI3K reg class IA (p85), Rb protein, IKK-alpha, PDCD4, RPS6, NMI, eIF4A, eIF4E, IFNAR2, CREB1, AKT(PKB), p27KIP1, c-Myc
99	Immune response_IL-4 signaling pathway	94	6.4E-05	0.00088	16	JAK2, p70 S6 kinase1, PI3K reg class IA (p85-alpha), PKC, MEKK1(MAP3K1), FKHR, PDE3B, IKK-alpha, Bcl-2, CREB1, CDC42, AKT(PKB), PDE4, NFKBIA, Rac1, IP3 receptor
100	EGFR family signaling in pancreatic cancer	75	6.4E-05	0.00088	14	COX-2 (PTGS2), I-kB, JAK2, PI3K reg class IA (p85), p70 S6 kinase1, Rb protein, IKK-alpha, Bcl-2, gp91-phox, AKT(PKB), p27KIP1, c-Myc, Rac1, MEK4(MAP2K4)

Supplementary Table S5. Enrichment by Pathway Maps of 1093 upregulated genes (Pattern 2).

#	Maps	Total	p-value	FDR	In Data	Network Objects from Active Data
1	Immune response_Platelet activating factor/ PTAFR pathway signaling	55	9.312E-11	1.237E-07	16	STAT3, STAT5, PKA-reg (cAMP-dependent), PI3K cat class IA, NF-kB, PLC-beta, NF-kB p50/p65, Beta-arrestin1, NF-AT1(NFATC2), c-Src, Calcineurin B (regulatory), p38 MAPK, G-protein alpha-i family, Adenylate cyclase, PKA-cat (cAMP-dependent), NF-AT
2	Cytoskeleton remodeling_Regulation of actin cytoskeleton organization by the kinase effectors of Rho GTPases	58	2.029E-08	1.347E-05	14	ARPC1B, Cortactin, DMPK, Alpha-actinin, Rac1-related, Cdc42 subfamily, Vinculin, TC10, Rac3, Spectrin, MLCK, MRLC, MRCK, PRK1
3	Development_Thromboxane A2 signaling pathway	50	2.256E-07	8.173E-05	12	GSK3 alpha/beta, MSK1/2 (RPS6KA5/4), PKA-reg (cAMP-dependent), PI3K cat class IA, G-protein alpha-s, PLC-beta, TBXA2R, c-Src, p38 MAPK, G-protein alpha-i family, Adenylate cyclase, PKA-cat (cAMP-dependent)
4	Blood coagulation_GPCRs in platelet aggregation	71	3.077E-07	8.173E-05	14	PKA-reg (cAMP-dependent), G-protein alpha-z, G-protein alpha-s, Prostacyclin receptor, alpha-IIb/beta-3 integrin, ITGA2B, TBXA2R, ITGB3, G-protein alpha-i family, Adenylate cyclase, PKA-cat (cAMP-dependent), MRLC, P2Y1, PLC-beta2
5	Signal transduction_Adenosine A2B receptor signaling pathway	71	3.077E-07	8.173E-05	14	VEGF-A, PKA-reg (cAMP-dependent), PI3K cat class IA, GSK3 beta, G-protein alpha-s, NF-kB1 (p105), PLC-beta, NF-kB p50/p65, PDK (PDPK1), Calcineurin B (regulatory), p38 MAPK, Adenylate cyclase, PKA-cat (cAMP-dependent), eNOS
6	Regulation of CFTR activity (normal and CF)	62	3.853E-07	8.527E-05	13	Casein kinase II, alpha chains, LPAR2, Casein kinase II, beta chain (Phosvitin), PP2A regulatory, PKA-reg type II (cAMP-dependent), PKA-reg (cAMP-dependent), PP2C, G-protein alpha-s, COMMD1 (MURR1), G-protein alpha-i family, Adenylate cyclase, PKA-cat (cAMP-dependent), Tubulin (in microtubules)
7	Development_Thrombospondin 1 signaling	36	5.338E-07	9.128E-05	10	VEGF-A, PKA-reg (cAMP-dependent), Caspase-8, Thrombospondin 1, ITGB3, p38 MAPK, G-protein alpha-i family, Adenylate cyclase, PKA-cat (cAMP-dependent), eNOS
8	Neurophysiological process_Constitutive and regulated NMDA receptor trafficking	65	6.870E-07	9.128E-05	13	Casein kinase II, alpha chains, PKA-reg (cAMP-dependent), G-protein alpha-s, PLC-beta, c-Src, Calcineurin B (regulatory), NR2A, NR2, PP1-cat, G-protein alpha-i family, Adenylate cyclase, PKA-cat (cAMP-dependent), MALS

Supplementary Table S5. (Continued)

#	Maps	Total	p-value	FDR	In Data	Network Objects from Active Data
9	PGE2 pathways in cancer	55	6.874E-07	9.128E-05	12	COX-1 (PTGS1), VEGF-A, PKA-reg (cAMP-dependent), GSK3 beta, G-protein alpha-s, Beta-arrestin1, PDK (PDPK1), c-Src, Axin, G-protein alpha-i family, Adenylate cyclase, PKA-cat (cAMP-dependent)
10	Development_WNT/Beta-catenin signaling in the cytoplasm	55	6.874E-07	9.128E-05	12	Casein kinase II, alpha chains, GSK3 alpha/beta, Axin2, Axin1, Casein kinase I delta, GSK3 beta, Hck, G-protein alpha-s, c-Src, WNT, Axin, Casein kinase II
11	Platelet activation as a result of endothelial dysfunction after stenting	56	8.454E-07	1.021E-04	12	PKA-reg (cAMP-dependent), PDE2A, G-protein alpha-s, Prostacyclin receptor, alpha-IIb/beta-3 integrin, TBXA2R, G-protein alpha-i family, Adenylate cyclase, PKA-cat (cAMP-dependent), P2Y1, Glycoprotein VI, PLC-beta2
12	Signal transduction_Adenosine A3 receptor signaling pathway	48	1.193E-06	1.320E-04	11	STAT3, Rho GTPase, VEGF-A, PI3K cat class IA, GSK3 beta, RelA (p65 NF-kB subunit), PLC-beta, PDK (PDPK1), p38 MAPK, G-protein alpha-i family, Adenylate cyclase
13	PDE4 regulation of cyto/chemokine expression in inflammatory skin diseases	50	1.843E-06	1.849E-04	11	NF-kB p50/p50, PKA-reg (cAMP-dependent), RelA (p65 NF-kB subunit), NF-kB1 (p50), NF-kB p50/p65, NF-AT1(NFATC2), PKA-cat alpha, p38 MAPK, G-protein alpha-i family, Adenylate cyclase, PKA-cat (cAMP-dependent)
14	Apoptosis and survival_Phosphorylation in TNF-alpha-induced NF-kB signaling	41	1.989E-06	1.849E-04	10	PKA-reg (cAMP-dependent), PI3K cat class IA, RelA (p65 NF-kB subunit), NF-kB p50/p65, PDK (PDPK1), PKA-cat alpha, c-Src, Adenylate cyclase, Casein kinase II, alpha chain (CSNK2A1), NF-kB p65/p65
15	Signal transduction_MIF signaling pathway	61	2.224E-06	1.849E-04	12	PI3K cat class IA, GSK3 beta, RelA (p65 NF-kB subunit), NF-kB, CD74, PLC-beta, Beta-arrestin1, PDK (PDPK1), CD74-ICD, SFK, c-Src, G-protein alpha-i family
16	Signal transduction_PKA signaling	51	2.271E-06	1.849E-04	11	GSK3 alpha/beta, PP2A regulatory, PKA-reg type II (cAMP-dependent), PKA-reg (cAMP-dependent), p90RSK1, G-protein alpha-s, PDK (PDPK1), PKA-cat alpha, G-protein alpha-i family, Adenylate cyclase, PKA-cat (cAMP-dependent)
17	FAK1 signaling in melanoma	42	2.524E-06	1.849E-04	10	VEGF-A, RelA (p65 NF-kB subunit), NF-kB, CAS-L, NF-kB1 (p50), NF-kB p50/p65, ITGA5, c-Src, ITGB3, PLAU (UPA)
18	Signal transduction_Adenosine A1 receptor signaling pathway	62	2.665E-06	1.849E-04	12	STAT3, PKA-reg (cAMP-dependent), NF-kB, PLC-beta, PDK (PDPK1), SFK, c-Src, p38 MAPK, G-protein alpha-i family, Adenylate cyclase, PKA-cat (cAMP-dependent), PLC-beta2

Supplementary Table S5. (Continued)

#	Maps	Total	p-value	FDR	In Data	Network Objects from Active Data
19	Signal transduction_Additional pathways of NF-kB activation (in the cytoplasm)	52	2.784E-06	1.849E-04	11	PKA-reg (cAMP-dependent), PI3K cat class IA, RelA (p65 NF-kB subunit), p90RSK1, NF-kB1 (p50), NF-kB p50/p65, PDK (PDPK1), PKA-cat alpha, c-Src, Adenylate cyclase, Casein kinase II, alpha chain (CSNK2A1)
20	Signal transduction_Adenosine A2A receptor signaling pathway	52	2.784E-06	1.849E-04	11	PKA-reg (cAMP-dependent), GSK3 beta, RelA (p65 NF-kB subunit), G-protein alpha-s, NF-kB1 (p50), SFK, c-Src, p38 MAPK, Adenylate cyclase, PKA-cat (cAMP-dependent), eNOS
21	Development_Role of HDAC and calcium/calmodulin-dependent kinase (CaMK) in control of skeletal myogenesis	53	3.396E-06	2.066E-04	11	CARM1, PI3K cat class IA, HDAC7, MEF2, p38beta (MAPK11), HDAC5, NF-AT1(NFATC2), IGF-1, PDK (PDPK1), Calcineurin B (regulatory), CaMKK
22	Immune response_ETV3 affect on CSF1-promoted macrophage differentiation	19	3.423E-06	2.066E-04	7	MSK1/2 (RPS6KA5/4), N-CoR, HDAC5, Sin3A, SMRT, p38 MAPK, PLAU (UPA)
23	Development_Transcriptional regulation of megakaryopoiesis	35	4.056E-06	2.342E-04	9	c-MPL, von Willebrand factor, alpha-IIb/beta-3 integrin, ITGA2B, TAL1, GATA-2, THAS, GP-IX, Glycoprotein VI
24	Tau pathology in Alzheimer disease	55	4.981E-06	2.756E-04	11	GSK3 alpha/beta, Casein kinase I delta, GSK3 beta, MARK2, PP2C, p38beta (MAPK11), Caspase-8, NR2, p38 MAPK, MAP1LC3A, Tubulin (in microtubules)
25	High shear stress-induced platelet activation	46	6.109E-06	3.187E-04	10	Ephrin-B1, Alpha-actinin, von Willebrand factor, alpha-IIb/beta-3 integrin, TBXA2R, Vinculin, ITGB3, P-selectin, GP-IX, G-protein alpha-i family
26	Development_Thrombopoietin signaling via ERK1/2 and PI3K	67	6.240E-06	3.187E-04	12	c-MPL, VEGF-A, PI3K cat class IA, GSK3 beta, RelA (p65 NF-kB subunit), ITGA2B, PDK (PDPK1), TAL1, PP1-cat, GP-IX, p38 MAPK, Glycoprotein VI
27	CHDI_Correlations from Replication data_Causal network (positive correlations)	79	6.837E-06	3.363E-04	13	PSMC2, MSK1/2 (RPS6KA5/4), PI3K cat class IA, IRAK1/2, HDAC7, MEF2, NF-kB, NF-AT1(NFATC2), HIP1, Calcineurin B (regulatory), NR2A, NR2, p38 MAPK
28	Development_VEGF signaling via VEGFR2 - generic cascades	93	8.903E-06	4.222E-04	14	COX-1 (PTGS1), VEGF-A, PI3K cat class IA, GSK3 beta, NF-kB p50/p65, Vinculin, PDK (PDPK1), c-Src, Calcineurin B (regulatory), p38 MAPK, p90Rsk, PLAU (UPA), MLCK, eNOS
29	Reproduction_Progesterone-mediated oocyte maturation	40	1.329E-05	5.956E-04	9	PKA-reg (cAMP-dependent), GSK3 beta, p90RSK1, PKA-cat alpha, c-Src, G-protein alpha-i family, Adenylate cyclase, PKA-cat (cAMP-dependent), p90Rsk

Supplementary Table S5. (Continued)

#	Maps	Total	p-value	FDR	In Data	Network Objects from Active Data
30	Apoptosis and survival_HTR1A signaling	50	1.345E-05	5.956E-04	10	STAT3, PP2A regulatory, PKA-reg (cAMP-dependent), NF-kB, NF-kB p50/p65, PDK (PDPK1), c-Src, G-protein alpha-i family, Adenylate cyclase, PKA-cat (cAMP-dependent)
31	CHDI_Correlations from Discovery data_Causal network	73	1.559E-05	6.512E-04	12	PDGF-A, PI3K cat class IA, GSK3 beta, MEF2, HDAC5, Sin3A, PLC-beta, c-Src, WNT, Axin, G-protein alpha-i family, p90Rsk
32	Cytoskeleton remodeling_Substance P mediated membrane blebbing	16	1.576E-05	6.512E-04	6	Dynamin-1, Tubulin alpha, Dynamin, MLCK, MRLC, Tubulin (in microtubules)
33	Immune response_Histamine signaling in dendritic cells	51	1.618E-05	6.512E-04	10	PKA-reg (cAMP-dependent), RelA (p65 NF-kB subunit), G-protein alpha-s, PLC-beta, NF-kB p50/p65, NF-AT1(NFATC2), PKA-cat alpha, G-protein alpha-i family, Adenylate cyclase, PKA-cat (cAMP-dependent)
34	Cytoskeleton remodeling_Reverse signaling by Ephrin-B	32	1.766E-05	6.897E-04	8	GSK3 beta, Tubulin alpha, c-Src, Ephrin-B, Axin, G-protein alpha-i family, WIRE, Tubulin (in microtubules)
35	Development_PEDF signaling	99	1.857E-05	7.045E-04	14	STAT3, NF-kB p50/p50, N-CoR, PI3K cat class IA, GSK3 beta, RelA (p65 NF-kB subunit), NF-kB, NF-kB1 (p50), NF-kB p50/p65, PDK (PDPK1), Fra-2, p38 MAPK, PKA-cat (cAMP-dependent), Cathepsin D
36	Neuroprotective action of lithium	63	1.955E-05	7.212E-04	11	VEGF-A, GSK3 beta, MKK7 (MAP2K7), c-Src, Calcineurin B (regulatory), NR2A, WNT, NR2, PP1-cat, p38 MAPK, Axin
37	Cell cycle_Influence of Ras and Rho proteins on G1/S Transition	53	2.309E-05	8.286E-04	10	STAT3, PI3K cat class IA, GSK3 beta, RelA (p65 NF-kB subunit), NF-kB p50/p65, PDK (PDPK1), DIA1, Tob1, MLCK, MRLC
38	Nociception_Nociceptin receptor signaling	76	2.376E-05	8.304E-04	12	STAT3, PKA-reg (cAMP-dependent), G-protein alpha-z, PLC-beta, NF-kB p50/p65, Beta-arrestin1, c-Src, p38 MAPK, G-protein alpha-i family, Adenylate cyclase, PKA-cat (cAMP-dependent), p90Rsk
39	Ovarian cancer (main signaling cascades)	65	2.656E-05	8.817E-04	11	LPAR2, PKA-reg (cAMP-dependent), PI3K cat class IA, GSK3 beta, NF-kB, K-RAS, Endothelin-1, c-Src, G-protein alpha-i family, PKA-cat (cAMP-dependent), PLAU (UPA)
40	Cell adhesion_PLAU signaling	65	2.656E-05	8.817E-04	11	Casein kinase II, alpha chains, STAT3, Casein kinase II, beta chain (Phosvitin), PI3K cat class IA, NF-kB, MYLK1, c-Src, G-protein alpha-i family, PLAU (UPA), MLCK, MRLC
41	K-RAS signaling in pancreatic cancer	44	3.015E-05	9.765E-04	9	GSK3 alpha/beta, MIRK, VEGF-A, RelA (p65 NF-kB subunit), NF-kB p50/p65, K-RAS, Thrombospondin 1, PDK (PDPK1), PLAU (UPA)

Supplementary Table S5. (Continued)

#	Maps	Total	p-value	FDR	In Data	Network Objects from Active Data
42	Development_Fetal brown fat cell differentiation	55	3.238E-05	1.024E-03	10	STAT5A, PKA-reg (cAMP-dependent), PI3K cat class IA, GSK3 beta, G-protein alpha-s, IGF-1, PDK (PDPK1), FASN, Adenylate cyclase, PKA-cat (cAMP-dependent)
43	IL-6 signaling pathway in lung cancer	35	3.578E-05	1.100E-03	8	STAT3, VEGF-A, PI3K cat class IA, NF-kB, PDK (PDPK1), c-Src, p38 MAPK, IL6RA
44	Development_Negative regulation of WNT/Beta-catenin signaling at the receptor level	45	3.645E-05	1.100E-03	9	GSK3 alpha/beta, SFRP, AP complex 2 medium (mu) chain, c-Src, WNT, Axin, SNX27, LRP1, Casein kinase II
45	Regulation of Beta-catenin activity in colorectal cancer	56	3.811E-05	1.125E-03	10	LPAR2, PKA-reg (cAMP-dependent), PI3K cat class IA, GSK3 beta, G-protein alpha-s, PLC-beta, Axin, MAML1, Adenylate cyclase, PKA-cat (cAMP-dependent)
46	Immune response_IL-3 signaling via JAK/STAT, p38, JNK and NF-kB	93	4.168E-05	1.203E-03	13	Ephrin-B1, STAT3, STAT5A, STAT5, PI3K cat class IA, NF-kB, NF-kB p50/p65, MKK7 (MAP2K7), c-Src, CSF2RB, P-selectin, p38 MAPK, SPECC1
47	Apoptosis and survival_IL-17-induced CIKS-independent signaling pathways	46	4.383E-05	1.211E-03	9	STAT3, PI3K cat class IA, GSK3 beta, NF-kB, NF-kB p50/p65, PDK (PDPK1), p38 MAPK, p90Rsk, C/EBPdelta
48	Development_Beta adrenergic receptors in brown adipocyte differentiation	36	4.451E-05	1.211E-03	8	PKA-reg (cAMP-dependent), G-protein alpha-s, FASN, p38 MAPK, Adenylate cyclase, PKA-cat (cAMP-dependent), PRKAR1A, C/EBPdelta
49	Development_Cytokine-mediated regulation of megakaryopoiesis	57	4.470E-05	1.211E-03	10	STAT3, c-MPL, STAT5, PI3K cat class IA, GSK3 beta, NF-kB, alpha-IIb/beta-3 integrin, ITGA2B, sIL6-RA, PP1-cat
50	Oxidative stress_ROS-induced cellular signaling	108	5.016E-05	1.314E-03	14	Casein kinase II, alpha chains, PLK3 (CNK), GADD45 alpha, VEGF-A, PKA-reg (cAMP-dependent), GSK3 beta, RelA (p65 NF-kB subunit), NF-kB, KEAP1, NF-kB p50/p65, PKA-cat alpha, FASN, c-Src, p38 MAPK
51	Neurophysiological process_Kappa-type opioid receptor signaling in the central nervous system	82	5.175E-05	1.314E-03	12	MSK1/2 (RPS6KA5/4), PKA-reg (cAMP-dependent), G-protein alpha-z, PLC-beta, Beta-arrestin1, PDK (PDPK1), SERT, c-Src, G-protein alpha-i family, Adenylate cyclase, PKA-cat (cAMP-dependent), p90Rsk
52	Immune response_IL-1 signaling pathway	82	5.175E-05	1.314E-03	12	PI3K cat class IA, RelA (p65 NF-kB subunit), NF-kB, NF-kB1 (p105), NF-kB1 (p50), ECSIT, NF-kB p50/p65, PDK (PDPK1), MYLK1, PLAU (UPA), MEK4/7, IRAK1

Supplementary Table S5. (Continued)

#	Maps	Total	p-value	FDR	In Data	Network Objects from Active Data
53	Canonical Leptin pathways in breast cancer	47	5.243E-05	1.314E-03	9	STAT3, VEGF-A, PI3K cat class IA, GSK3 beta, RelA (p65 NF-kB subunit), NF-kB, NF-kB p50/p65, PDK (PDPK1), Axin
54	Development_Negative feedback regulation of WNT/Beta-catenin signaling	37	5.495E-05	1.327E-03	8	GSK3 alpha/beta, Axin2, c-Src, WNT, HUWE1, Axin, G-protein alpha-i family, Casein kinase II
55	Role of red blood cell adhesion to endothelium in vaso-occlusion in Sickle cell disease	37	5.495E-05	1.327E-03	8	PKA-reg (cAMP-dependent), von Willebrand factor, alpha-IIb/beta-3 integrin, Thrombospondin 1, c-Src, P-selectin, G-protein alpha-i family, PKA-cat (cAMP-dependent)
56	Muscle contraction_GPCRs in the regulation of smooth muscle tone	83	5.848E-05	1.387E-03	12	PKA-reg (cAMP-dependent), G-protein alpha-s, Prostacyclin receptor, PLC-beta, TBXA2R, Endothelin-1, G-protein alpha-i family, Adenylate cyclase, PKA-cat (cAMP-dependent), MLCK, MRLC, Telokin
57	Inhibition of apoptosis in pancreatic cancer	59	6.081E-05	1.405E-03	10	STAT3, STAT5, VEGF-A, PI3K cat class IA, RelA (p65 NF-kB subunit), ALOX12, K-RAS, IGF-1, G-protein alpha-i family, Casein kinase II, alpha chain (CSNK2A1)
58	Immune response_B cell antigen receptor (BCR) pathway	110	6.159E-05	1.405E-03	14	GSK3 alpha/beta, GSK3 beta, RelA (p65 NF-kB subunit), NF-kB, CD79A, NF-kB1 (p50), NF-kB p50/p65, K-RAS, PDK (PDPK1), Calcineurin B (regulatory), p38 MAPK, PIP5KIII, PI3K cat class IA (p110-delta), CARD11
59	Muscle contraction_Relaxin signaling pathway	48	6.242E-05	1.405E-03	9	VEGF-A, PKA-reg (cAMP-dependent), NF-kB, G-protein alpha-s, Endothelin-1, G-protein alpha-i family, PKA-cat (cAMP-dependent), p90Rsk, eNOS
60	Translation_Regulation of EIF2 activity	38	6.734E-05	1.491E-03	8	Casein kinase II, alpha chains, GSK3 alpha/beta, Casein kinase II, beta chain (Phosvitin), Casein kinase I, PI3K cat class IA, PDK (PDPK1), PP1-cat, PP1-cat alpha
61	Blood coagulation_Platelet microparticle generation	72	7.078E-05	1.541E-03	11	Alpha-fodrin, von Willebrand factor, PLC-beta, alpha-IIb/beta-3 integrin, TBXA2R, MYLK1, P-selectin, GP-IX, PIP5KIII, P2Y1, Glycoprotein VI
62	Transcription_N-CoR/ SMRT complex-mediated epigenetic gene silencing	49	7.396E-05	1.559E-03	9	TBL1X, N-CoR, NF-kB, HDAC5, Sin3A, PBX1, BAF170, SMRT, Histone H2B
63	Transcription_CREB signaling pathway	49	7.396E-05	1.559E-03	9	MSK1/2 (RPS6KA5/4), PKA-reg (cAMP-dependent), PI3K cat class IA, G-protein alpha-s, PDK (PDPK1), PP1-cat, p38 MAPK, PKA-cat (cAMP-dependent), p90Rsk

Supplementary Table S5. (Continued)

#	Maps	Total	p-value	FDR	In Data	Network Objects from Active Data
64	Apoptosis and survival_nAChR in apoptosis inhibition and cell cycle progression	29	7.649E-05	1.587E-03	7	GSK3 alpha/beta, GSK3 alpha, PI3K cat class IA, GSK3 beta, Beta-arrestin1, PDK (PDPK1), c-Src
65	Reproduction_Gonadotropin-releasing hormone (GnRH) signaling	73	8.057E-05	1.646E-03	11	MKP-2, Dynamin-1, PKA-reg (cAMP-dependent), G-protein alpha-s, HDAC5, PLC-beta, MKK7 (MAP2K7), c-Src, Adenylate cyclase, PKA-cat (cAMP-dependent), p90Rsk
66	Development_GM-CSF signaling	50	8.724E-05	1.679E-03	9	STAT3, STAT5A, STAT5, PI3K cat class IA, Hck, GM-CSF receptor, NF-kB, CSF2RB, CSF2RA
67	Angiogenesis in HCC	50	8.724E-05	1.679E-03	9	Ephrin-B1, STAT3, Epo receptor, STAT5, VEGF-A, NF-kB, NF-kB1 (p50), Ephrin-B, Securin
68	Immune response_Function of MEF2 in T lymphocytes	50	8.724E-05	1.679E-03	9	CABIN1, CARM1, HDAC7, MEF2, p38beta (MAPK11), HDAC5, Sin3A, NF-AT1(NFATC2), CaMKK
69	HBV-dependent NF-kB and PI3K/AKT pathways leading to HCC	50	8.724E-05	1.679E-03	9	PI3K cat class IA, GSK3 beta, RelA (p65 NF-kB subunit), NF-kB, NF-kB1 (p105), NF-kB p50/p65, PDK (PDPK1), c-Src, PLAU (UPA)
70	Signal transduction_Additional pathways of NF-kB activation (in the nucleus)	30	9.658E-05	1.832E-03	7	PKA-reg (cAMP-dependent), RelA (p65 NF-kB subunit), p90RSK1, NF-kB1 (p50), NF-kB p50/p65, PKA-cat alpha, Adenylate cyclase
71	Development_Negative regulation of WNT/Beta-catenin signaling in the nucleus	89	1.169E-04	1.980E-03	12	TBL1X, VEGF-A, Casein kinase I delta, GSK3 beta, CHD8, TLE, HIC5, Oct-3/4, WNT, Axin, Histone H1, eNOS
72	G protein-coupled receptors signaling in lung cancer	76	1.172E-04	1.980E-03	11	STAT3, VEGF-A, PKA-reg (cAMP-dependent), RelA (p65 NF-kB subunit), Galpha(q)-specific peptide GPCRs, G-protein alpha-s, Endothelin-1, PDK (PDPK1), c-Src, G-protein alpha-i family, PKA-cat (cAMP-dependent)
73	Signal transduction_Angiotensin II/AGTR1 signaling via Notch, Beta-catenin and NF-kB pathways	76	1.172E-04	1.980E-03	11	Axin2, VEGF-A, GSK3 beta, RelA (p65 NF-kB subunit), NCOA1 (SRC1), p90RSK1, NF-kB, NF-kB p50/p65, PDK (PDPK1), p38 MAPK, PKA-cat (cAMP-dependent)
74	Main growth factor signaling cascades in multiple myeloma cells	41	1.192E-04	1.980E-03	8	STAT3, GSK3 alpha/beta, VEGF-A, PI3K cat class IA, NF-kB, K-RAS, IGF-1, PDK (PDPK1)

Supplementary Table S5. (Continued)

#	Maps	Total	p-value	FDR	In Data	Network Objects from Active Data
75	Immune response_TLR2-induced platelet activation	41	1.192E-04	1.980E-03	8	PI3K cat class IA, PLC-beta, alpha-IIb/beta-3 integrin, TBXA2R, P-selectin, p38 MAPK, P2X1, P2Y1
76	G-protein signaling_Proinsulin C-peptide signaling	52	1.199E-04	1.980E-03	9	PI3K cat class IA, ATP1A1, NF-kB, PLC-beta, NF-kB p50/p65, PDK (PDPK1), c-Src, G-protein alpha-i family, eNOS
77	Signal transduction_Activation of PKC via G-Protein coupled receptor	52	1.199E-04	1.980E-03	9	GSK3 beta, HDAC7, MEF2, NF-kB, PLC-beta, NF-AT1(NFATC2), c-Src, Calcineurin B (regulatory), MLCK
78	Immune response_Lysophosphatidic acid signaling via NF-kB	52	1.199E-04	1.980E-03	9	LPAR2, VEGF-A, RelA (p65 NF-kB subunit), NF-kB, NF-kB p50/p65, MKK7 (MAP2K7), c-Src, p38 MAPK, p90Rsk
79	Glucagon-induced glucose upregulation in type 2 diabetes in liver	52	1.199E-04	1.980E-03	9	PYC, PKA-reg (cAMP-dependent), G-protein alpha-s, PLC-beta, Calcineurin B (regulatory), G-protein alpha-i family, Adenylate cyclase, PKA-cat (cAMP-dependent), Casein kinase II
80	Role of IL-2 in the enhancement of NK cell cytotoxicity in multiple sclerosis	31	1.208E-04	1.980E-03	7	STAT5, PI3K cat class IA, NF-kB, NF-kB1 (p50), NF-kB p50/p65, PDK (PDPK1), CARD11
81	Immune response_IL-23 signaling pathway	31	1.208E-04	1.980E-03	7	STAT3, STAT5, PI3K cat class IA, NF-kB, NF-kB p50/p65, PDK (PDPK1), IL-12RB1
82	ENaC regulation in normal and CF airways	53	1.396E-04	2.150E-03	9	PKA-reg (cAMP-dependent), PI3K cat class IA, G-protein alpha-s, PLC-beta, Endothelin-1, Furin, c-Src, Adenylate cyclase, PKA-cat (cAMP-dependent)
83	Non-genomic signaling of ESR2 (membrane) in lung cancer cells	53	1.396E-04	2.150E-03	9	VEGF-A, PKA-reg (cAMP-dependent), PI3K cat class IA, G-protein alpha-s, PDK (PDPK1), c-Src, G-protein alpha-i family, Adenylate cyclase, PKA-cat (cAMP-dependent)
84	Development_Role of IL-8 in angiogenesis	65	1.417E-04	2.150E-03	10	STAT3, SCAP, VEGF-A, PI3K cat class IA, NF-kB, PDK (PDPK1), FASN, c-Src, G-protein alpha-i family, CARD11
85	Muscle contraction_Regulation of eNOS activity in endothelial cells	65	1.417E-04	2.150E-03	10	VEGF-A, NOSIP, PI3K cat class IA, PLC-beta, NF-kB p50/p65, Endothelin-1, GATA-2, c-Src, G-protein alpha-i family, eNOS
86	NF-AT signaling in cardiac hypertrophy	65	1.417E-04	2.150E-03	10	PI3K cat class IA, GSK3 beta, HDAC7, G-protein alpha-s, HDAC5, Endothelin-1, IGF-1, c-Src, G-protein alpha-i family, CaMKK
87	Apoptosis and survival_BAD phosphorylation	42	1.425E-04	2.150E-03	8	PKA-reg (cAMP-dependent), PI3K cat class IA, PP2C, G-protein alpha-s, PDK (PDPK1), PKA-cat (cAMP-dependent), p90Rsk, PP1-cat alpha

Supplementary Table S5. (Continued)

#	Maps	Total	p-value	FDR	In Data	Network Objects from Active Data
88	TNF-alpha, IL-1 beta induce dyslipidemia and inflammation in obesity and type 2 diabetes in adipocytes	42	1.425E-04	2.150E-03	8	TNF-R2, PKA-reg (cAMP-dependent), APOE, NF-kB, NF-kB1 (p50), Beta-arrestin1, PKA-cat (cAMP-dependent), eNOS
89	Cell cycle_Role of Nek in cell cycle regulation	32	1.496E-04	2.233E-03	7	Tubulin beta, MAD1 (mitotic checkpoint), PI3K cat class IA, Tubulin alpha, PDK (PDPK1), Histone H1, Tubulin (in microtubules)
90	Apoptosis and survival_Beta-2 adrenergic receptor anti-apoptotic action	23	1.588E-04	2.317E-03	6	PKA-reg (cAMP-dependent), FOXO4, G-protein alpha-s, PDK (PDPK1), G-protein alpha-i family, PKA-cat (cAMP-dependent)
91	Transcription_ChREBP regulation pathway	23	1.588E-04	2.317E-03	6	AMPK gamma subunit, PKA-reg (cAMP-dependent), G-protein alpha-s, G-protein alpha-i family, PKA-cat (cAMP-dependent), Acyl-CoA synthetase
92	Canonical WNT signaling pathway in colorectal cancer	66	1.615E-04	2.319E-03	10	Axin2, VEGF-A, Axin1, PI3K cat class IA, GSK3 beta, CAS-L, K-RAS, SFRP2, WNT, Axin
93	Prostaglandins and leukotrienes-mediated induction of expression of mucins in normal and asthmatic epithelium	43	1.694E-04	2.319E-03	8	PKA-reg (cAMP-dependent), p90RSK1, NF-kB, G-protein alpha-s, PLC-beta, G-protein alpha-i family, Adenylate cyclase, PKA-cat (cAMP-dependent)
94	Role of cell adhesion in vaso-occlusion in Sickle cell disease	43	1.694E-04	2.319E-03	8	GLG1, PKA-reg (cAMP-dependent), alpha-IIb/beta-3 integrin, NF-kB p50/p65, Thrombospondin 1, ITGAL, P-selectin, PKA-cat (cAMP-dependent)
95	Role of platelets in the initiation of in-stent restenosis	43	1.694E-04	2.319E-03	8	PDGF-A, von Willebrand factor, alpha-IIb/beta-3 integrin, TBXA2R, P-selectin, GP-IX, P2Y1, Glycoprotein VI
96	Immune response_IL-15 signaling via JAK-STAT and PPAR cascades	43	1.694E-04	2.319E-03	8	STAT3, STAT5A, STAT5, CPT-1A, sIL-15RA, IL-15RA, FASN, ULK1
97	Role of platelets in allograft rejection	43	1.694E-04	2.319E-03	8	COX-1 (PTGS1), von Willebrand factor, alpha-IIb/beta-3 integrin, TBXA2R, P-selectin, GP-IX, P2Y1, Glycoprotein VI
98	Ligand-independent activation of Androgen receptor in Prostate Cancer	67	1.835E-04	2.417E-03	10	STAT3, STAT5A, PP2A regulatory, PI3K cat class IA, GSK3 beta, NCOA1 (SRC1), K-RAS, WNT3A, IGF-1, PDK (PDPK1)
99	B-regulatory cells and tumor cells intercellular interaction	67	1.835E-04	2.417E-03	10	STAT3, TNF-R2, VEGF-A, LTBR(TNFRSF3), OX40(TNFRSF4), RelA (p65 NF-kB subunit), GM-CSF receptor, NF-kB, p38 MAPK, NF-kB p65/p65

Supplementary Table S5. (Continued)

#	Maps	Total	p-value	FDR	In Data	Network Objects from Active Data
100	Deficient alpha-MSH signaling in melanoma	33	1.838E-04	2.417E-03	7	PKA-reg (cAMP-dependent), PI3K cat class IA, GSK3 beta, PDK (PDPK1), PKA-cat alpha, p38 MAPK, PKA-cat (cAMP-dependent)

Supplementary Table S6. Gene set class comparison analysis by cell type (BrB ArrayTools) of 2005 downregulated genes (Pattern 1).

GeneList GeneSets	Number of genes	Genes in data	LS permutation p-value	KS permutation p-value	Efron-Tibshirani's GSA test p-value	Annotation/Reference
GC_B_cell_U133Plus	36	RCBTB2, LNPEP, MYBL1, NFYB, PPP3CB, TIA1, STK17B, ATP8A1, PSIP1, KLF12, RRAS2, HERC4, USP53, ZNF280D, CCDC88A, MS4A7, PLEKHF2, CLIP4, SLTM, LYPLAL1, NCOA7, DENND1B, SGMS1, TRIM59, GPR160, UBE2J1, MTF2, HS2ST1, HOPX, RNF144B, ZNF141, LMO2, HSPB11, ZNF718, KATNAL1, LRMP	0.01569	0.03568	0.005	(Dave et al. NEJM 354:2431 (2006))(https://lymphochip.nih.gov/signaturedb/)
GC_T_helper_up2x_Chtanova	80	CD69, ITGAV, LNPEP, PIK3C2A, PLAG1, PPP1CC, PTPN2, CLIP1, TRAF5, TSPYL1, UBE2D3, ZMYM2, KAT2B, STK17B, HDAC4, TSC22D2, ATP8A1, VAV3, MSL3, KLF12, IKZF2, EFR3A, RNF19A, C11orf54, BAZ2B, ATAD2B, FAM134B, KLHL24, ZNF280D, ATG2B, TMEM55A, ERBB2IP, CLK4, MKL2, MIB1, ARRDC3, ZNF529, YTHDC2, PLEKHA3, GLCCI1, ARAP2, TC2N, JMY, FAM117B, DCP2, IRF8, TP53INP1, BTLA, ICOS, YPEL5, COBLL1, SH3YL1, MAGEH1, LY96, MBOAT1, IFNAR2, RBM5, NR3C1, ZNF226, SMAD1, DZIP3, JAZF1, TRIM13, IFRD1, FAM46A, STK39, SAT1, SH2D1A, RGS1, OMA1, AMPD3, MS4A1, NDUFB2, RAB27A, LRMP, DENND4C, ATP1B3, DUSP1, EIF5, AFF3	0.3691	0.52972	0.025	Genes preferentially expressed in TFH cells compared to all other subsets. The list was generated from genes upregulated in at least 9 out of 12 comparisons to the other T cell subsets (TEM, TCM, Th1a, Th2a, Th1b, Th2b;2samples each)

Supplementary Table S7. Gene set class comparison analysis by cell type (BrB ArrayTools) of 1093 upregulated genes (Pattern 2).

GeneList GeneSets	Number of genes	Genes in data	LS permutation p-value	KS permutation p-value	Efron-Tibshirani's GSA test p-value
HAY_BONE_MARROW_PLATELET	60	RTN2, GAS2L1, TPTEP1, SELP, BEND2, PTGIR, PARD3, TBXA2R, WBP2, TSA, CLU, SENCR, MYLK, NRGN, EMC3, LINC00853, ABLIM3, ITGB3, ZCCHC17, MOB3C, TNNC2, ESAM, DNM3, ABCC3, CTTN, SCGB1C1, GP6, CTDSPL, PDLIM7, TMEM91, SPARC, TREML1, CMTM5, PTGS1, SRC, PCP2, HEXIM2, PDGFA, VIL1, MYL9, CLDN5, SLC6A4, MTURN, GP9, ENDOD1, LY6G6F, PTCRA, PTPN18, ALOX12, KIFC3, SLC24A3, VCL, TMEM40, GNAZ, TUBA8, SPDYC, ACRBP, THEM5, BMP6, MAP3K7CL	0.04466	0.15867	0.145
HAY_BONE_MARROW_CD34_POS_MKP	8	ITGA2B, VWF, RAC3, TGFB1I1, ACTN1, MGLL, GATA2, PBX1	0.06908	0.5971	0.005

Supplementary Figures

Supplementary Figure S1

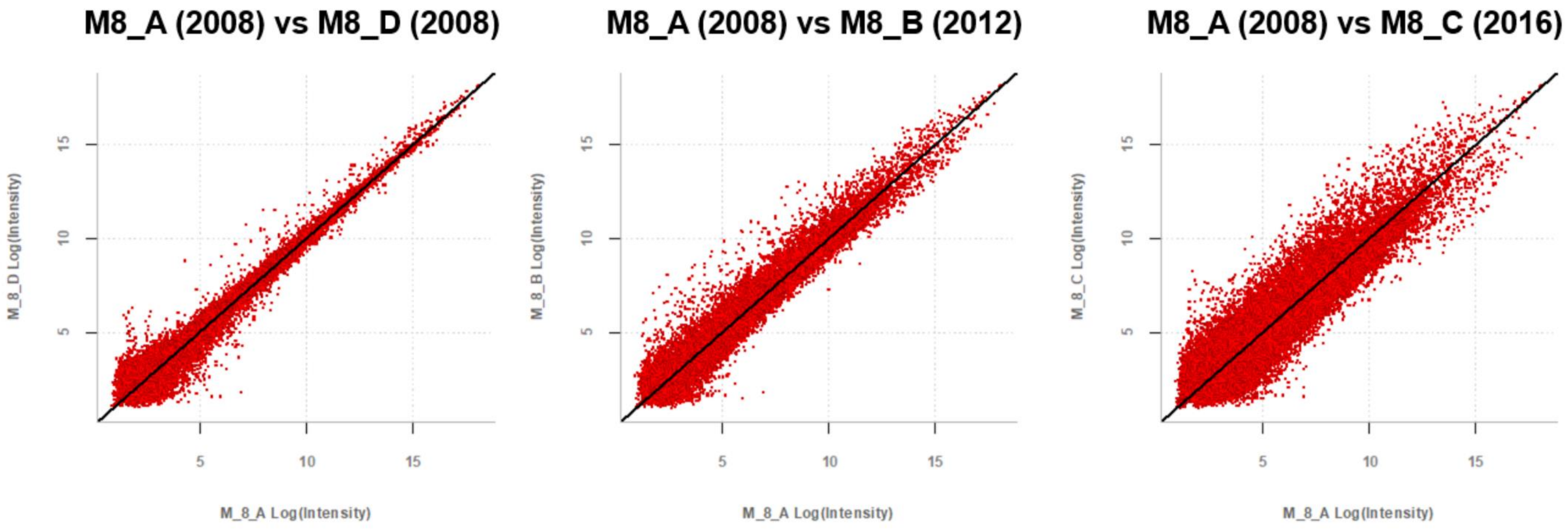

Supplementary Figure S1. Gene expression stability verified by technical replicates. We confirmed the reproducibility and stability of gene expression by DNA microarray analysis. The extracted blood RNA samples for each time point were analyzed twice for the transcribed mRNA; depicted in the figure are scatterplots of the expression data of one representative individual (M8): 1) Technical replicate: Microarray data M8_A vs. M8_D (both data were from two different technical replicates of the M8 sample collected in 2008), 2) experimental comparison for a different year: sample M8_A (sampled in 2008) vs. M8_B (sampled in 2012), and 3) experimental comparison for a different year: sample M8_A (sampled in 2008) vs. M8_C (sampled in 2016). A total of 30,748 genes, prior to filtration, were used to generate the scatterplots.
